# Discovery of acylphloroglucinol-based meroterpenoid enantiomers as KSHV inhibitors from *Hypericum japonicum*†

**DOI:** 10.1039/c8ra04073g

**Published:** 2018-07-02

**Authors:** Linzhen Hu, Yanfei Liu, Yanxing Wang, Zhenzhen Wang, Jinfeng Huang, Yongbo Xue, Junjun Liu, Zhenming Liu, Yong Chen, Yonghui Zhang

**Affiliations:** National & Local Joint Engineering Research Center of High-throughput Drug Screening Technology, Hubei Key Laboratory of Biotechnology of Chinese Traditional Medicine, School of Life Sciences, Hubei University Wuhan 430062 Hubei Province P. R. China cy101610@qq.com; Hubei Key Laboratory of Natural Medicinal Chemistry and Resource Evaluation, School of Pharmacy, Tongji Medical College, Huazhong University of Science and Technology Wuhan 430030 Hubei Province P. R. China zhangyh@mails.tjmu.edu.cn; The Central Hospital of Wuhan, Huazhong University of Science and Technology Wuhan 430014 Hubei Province P. R. China; State Key Laboratory of Natural and Biomimetic Drugs, School of Pharmaceutical Sciences, Peking University Beijing 100191 P. R. China zmliu@bjmu.edu.cn

## Abstract

Kaposi's sarcoma associated herpesvirus (KSHV) has gained considerable attention as a type of carcinogenic pathogen. Recent research suggests that KSHV has participated in the pathogenesis of Kaposi's sarcoma-related malignant neoplastic diseases. Viral lytic infection might be pivotal for the etiopathogenesis of KSHV-induced diseases; however, most clinical KSHV lytic replication inhibitors like ganciclovir, nelfinavir, or cidofovir do not restrain virus replication effectively enough to achieve clinical efficacy. In our continued pharmaceutical studies on Chinese herbal medicines, new acylphloroglucinol-based meroterpenoid enantiomers have been discovered from *Hypericum japonicum*. Most of these metabolites have potential inhibitory activities that target KSHV lytic replication. Amongst these analogues, compounds 1a and 1b possess an unreported ring system cyclopenta[*b*]chromene. Compounds 1a with 4a exhibit stronger inhibitory activities towards the lytic replication of KSHV in Vero cells. In addition, 1a and 4a have IC_50_ values of 8.30 and 4.90 μM and selectivity indexes of 23.49 and 25.70, respectively. Qualitative and quantitative SAR and molecular docking studies for acylphloroglucinol-based meroterpenoids with regard to anti-KSHV activity were conducted. An explanation for the variation in the activity and selectivity indexes was proposed in accordance with the predicted binding pose found with molecular docking to a putative target, thymidylate synthase (kTS). Compounds 1a and 4a have potential for further development and optimization of their anti-KSHV activities which could lead to new candidate drugs.

## Abstract 2

New enantiomers (1a/1b–4a/4b) were discovered from *Hypericum japonicum*. 1a/1b possessed a novel ring system cyclopenta[*b*]chromene. 1a and 4a exhibited promising anti-KSHV activities. QSAR studies for enantiomers on anti-KSHV activity were conducted.

## Graphical abstract

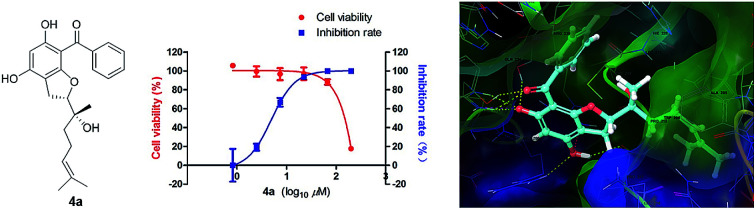

## Introduction

Kaposi's sarcoma-associated herpesvirus (KSHV), containing double-stranded DNA, is in the family of Herpesviridae and within the subfamily of gamma-herpesvirus.^1^ It is thought to participate in the pathogenesis of Kaposi's sarcoma-related malignancies, such as multicentric Castleman's disease, AIDS-related multicentric Castleman's disease, and primary effusion lymphoma.^1*a*,2^ Like other herpesviruses, KSHV has two life cycles of latency and lytic replication, and both contribute to virus-associated disease.^2*a*^ Generally, during the latent infection, KSHV is established as episomes *via* a certain amount of latent genes expressed to support viral latent replication. While in the lytic replication, viral genes, comprised of immediate early, early, and late phases, are expressed, leading to the production of mature progeny virions and ultimate lysis of cells.^2*b*^ Since viral lytic infection might be pivotal for the etiopathogenesis of KSHV-induced diseases, most clinical agents, including ganciclovir, nelfinavir, or cidofovir, target the inhibition of KSHV lytic replication.^3^ However, most of these antiviral drugs do not restrain virus replication effectively enough to achieve clinical efficacy.^3,4^ Therefore, additional antiviral regimens are needed which concentrate on developing candidate drugs or lead compounds which inhibit on KSHV lytic replication.

Natural products continue to arouse the interest of pharmacochemistry scientists due to their therapeutic applications and their use as an arsenal of sources for exploiting lead compounds.^5^ Our on-going course of systematic study on the genus *Hypericum* has led to an enormous amount of phloroglucinol derivatives that exhibit anti-virus activities.^6^ Amongst this genus, *Hypericum japonicum* Thunb (Guttiferae), a perennial or annual herb, is extensively distributed throughout North America, Oceania, and Asia.^7^ Extracts of the entire herb, also named “Tianjihuang” in Chinese, have been manufactured to be used by injection. Some metabolites isolated from *H. japonicum* have been demonstrated to be lead compounds with bioactivities such as anti-cancer, antimalarial, antibacterial, and anti-oxidative stress.^8^ With the intent of finding bioactive lead compounds from Chinese herbal medicine, our research team has investigated acylphloroglucinol-based compounds from *H. japonicum* and has discovered that most compounds exhibit potential anti-KSHV activities.^6*c*^ Our present study of this herb-medicine led to analogue enantiomer pairs 1a/1b–4a/4b, of which compound 1a/1b contains the novel ring system of a cyclopenta[*b*]chromene, and compounds 1a and 4a exhibit stronger inhibitory activities towards the lytic replication of KSHV in Vero cells, with IC_50_ values of 8.30 and 4.90 μM, and selectivity indexes of 23.49 and 25.70, respectively. Furthermore, the first qualitative and quantitative SAR and molecular docking studies for acylphloroglucinol-based meroterpenoids with regard to anti-KSHV activity were conducted. These studies suggest that 4a is able to anchor with kTS by hydrogen bonding to residues Arg199, Arg200, Pro217, Ser240 and Tyr282. An additional salt bridge between 5′-OH and Arg199 and a hydrophobic pocket formed by residues Phe115, Val158, Pro218 and other hydrophobic residues are also suggested.

## Results and discussion

**Fig. 1:**
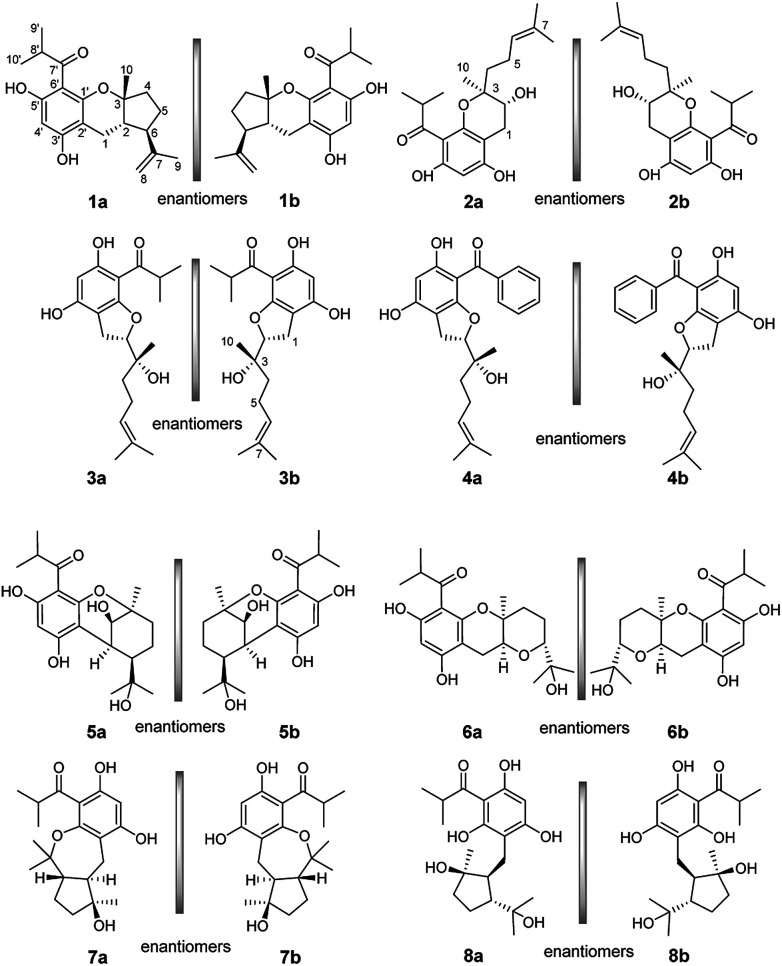
Chemical structures of compounds 1a/1b–8a/8b.

**Fig. 2:**
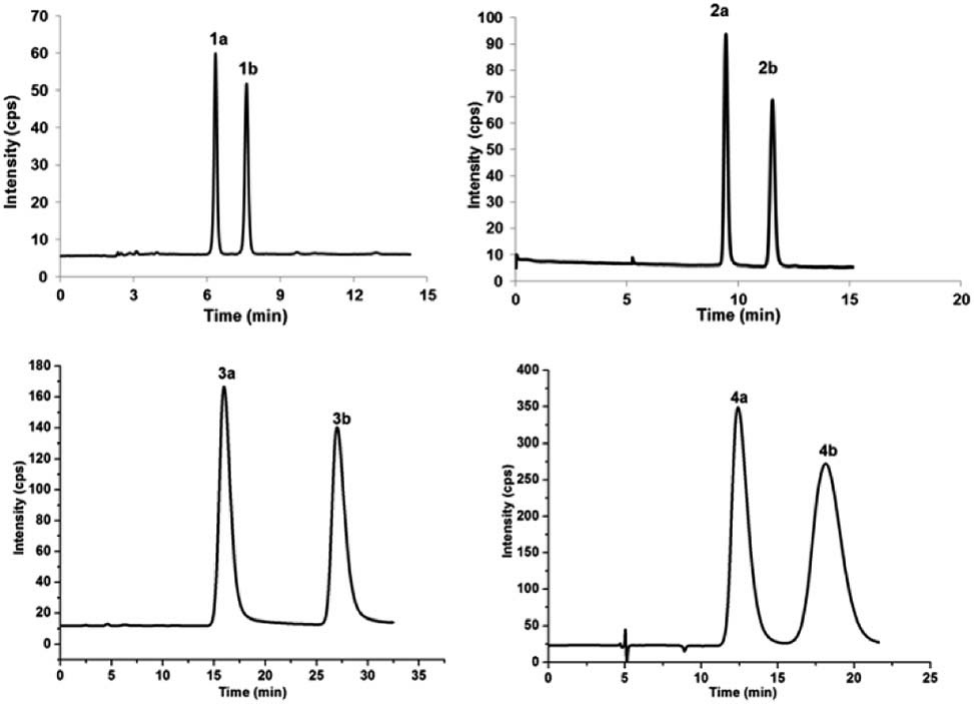
Chromatograms showing the enantioseparation of racemates 1a/1b–4a/4b. HPLC chromatographic specifications were the following: mobile phase of hexane/isopropanol 94/6 (v/v) for 1a/1b, 99/1 (v/v) for 2a/2b, 93.5/6.5 (v/v) for 3a/3b, and 98/2 (v/v) for 4a/4b; flow rate of 3.0 mL min^−1^; column temperature of 25 °C; UV detection at 240 nm; semipreparative chromatographic column, CHIRALPAK®IC column (10 × 250 mm, 5 μm particles, Daicel, China). All mass ratios of 1a/1b–4a/4b were roughly 1 : 1.

Enantiomeric metabolites are recognized by researchers. However, they still are infrequently studied due to the abundance of secondary metabolites.^9^ Often, both enantiomers have been discovered as either a scalemic or racemic mixture.^10^ Interestingly, another four pairs of new phloroglucinol-based terpenoid enantiomers, (±)-japonicols E–H (1a/1b–4a/4b), possessing diverse monoterpenoid skeletons, especially in 1a/1b, with the novel carbon skeleton of a cyclopenta[*b*]chromene, were obtained from *Hypericum japonicum*. The structures and absolute configurations were confirmed *via* extensive NMR spectroscopic data and calculated ECD analyses. Herein, the stereochemistry elucidation, biological activity measurements, as well as the Qualitative and quantitative structure–activity relationship (SAR) and molecular docking studies for these acylphloroglucinol-based meroterpenoids with regard to anti-KSHV activity are presented in detail.

(±)-Japonicols E–H (1a/1b–4a/4b, Fig. 1) were compared (Table 1) with NMR spectroscopic data of (±)-japonicols A–D (5a/5b–8a/8b, Fig. 1),^6*c*^ which indicated that the japonicols E–H, the other four pairs of acylphloroglucinol-based meroterpenoid enantiomers co-occurring in the same herbs, owned the core skeleton, *viz.*, acylphloroglucinol functional groups. These four pairs of new acylphloroglucinol-based meroterpenoid enantiomers, as racemic mixtures, were successfully isolated with almost 100% values for enantiomeric excess (ee) for each pair *via* separation using a CHIRALPAK®IC preparative column (10 × 250 mm, 5 μm particles, Daicel, China) eluted through HPLC. More information is presented in Fig. 2.

**Table 1:** ^1^H NMR (400 MHz) and ^13^C (100 MHz) data for japonicols E–H (1–4) (*δ* in ppm, *J* in Hz)^a^

No.	1		2		3		4	
	*δ*_H_	*δ*_C_	*δ*_H_	*δ*_C_	*δ*_H_	*δ*_C_	*δ*_H_	*δ*_C_
1	2.35 dd (14.1, 10.6), 2.68 dd (14.1, 10.6)	22.3	2.85 dd (16.6, 5.8), 2.45 dd (16.6, 8.1)	26.8	3.02 m	27.7	3.04 m	27.7
2	1.91 dd (10.8, 4.5)	51.7	3.86 dd(8.1, 5.8)	67.2	4.70 dd (9.2, 8.2)	91.9	4.74 t (8.7)	91.9
3		81.4		81.6		74.4		74.4
4	1.76 m	42.4	1.80 m, 1.72 m	39.1	1.52 m	39.5	1.54 m	39.5
5	1.46 d (13.6), 1.82 m	29.5	2.20 m, 2.16 m	23.2	2.11 m	23.1	2.13 m	23.1
6	2.54 dd (19.7, 8.7)	54.9	5.14 t (7.2)	125.1	5.13 m	125.7	5.14 t (7.1)	125.7
7		148.7		132.8		132.6		132.6
8	4.69 s	111.2	1.62 s	17.9	1.63 s	17.8	1.64 s	17.8
	4.80 s							
9	1.72 s	18.9	1.69 s	26.0	1.68 s	26.0	1.69 s	26.0
10	1.00 s	28.7	1.27 s	18.7	1.20 s	22.0	1.23 s	22.1
1′		163.5		157.2		168.2		168.5
2′		108.0		101.5		105.6		106.7
3′		164.1		164.3		161.4		159.2
4′	n.d.	n.d.	n.d.	n.d.	n.d.	n.d.	n.d.	n.d.
5′		162.2		n.d.		165.3		164.0
6′		104.8		n.d.		105.5		105.8
7′		212.1		211.0		212.1		201.0
8′	4.02 sept (6.8)	40.1	3.95 sept (6.7)	40.1	3.98 sept (6.7)	40.1		
9′	1.12 d (6.8)	19.9	1.13 d (6.7)	19.9	1.12 d (6.7)	19.9		
10′	1.14 d (6.8)	20.0	1.15 d (6.7)	20.4	1.14 d (6.7)	19.9		
1′′								143.3
2′′							7.58 d (8.3)	129.4
3′′							7.39 m	128.8
4′′							7.46 m	132.6
5′′							7.37 m	128.8
6′′							7.56 d (8.3)	129.4

a Record in methanol-*d*_4_, n.d. means no detected.

(±)-Japonicol E (1a/1b), an off-white amorphous powder, with the molecular formula of C_20_H_26_O_4_, was inferred *via* HRESIMS data (*m*/*z* 331.1963 [M + H]^+^, calcd 331.1909). The FTIR spectrogram shows that the characteristic absorptions for hydroxy (3279 cm^−1^), carbonyl (1626 cm^−1^), and aromatic ring (1593 and 1438 cm^−1^) groups on the molecule are presented. Analyses of the ^1^H and ^13^C NMR, including HSQC spectrum, suggested the presence of a acylphloroglucinol base along with a carbonyl (*δ*_C_ 212.1), aromatic carbons (*δ*_C_ 163.5, 108.0, 164.1, 162.2, 104.8, and one carbon resonance of C-4′ is not displayed in the one-dimensional NMR spectrum, but the existence of this carbon could be deduced from the HRESIMS data), two methyls (*δ*_H_ 1.12, d, *J* = 6.8 Hz; 1.14, d, *J* = 6.8 Hz), and one methine (*δ*_H_ 4.02, *J* = 6.8 Hz). These analyses, together with the close carbon chemical shifts of (±)-japonicols A–D,^6*c*^ lead to the acylphloroglucinol base of the molecule. The planar structure of its monoterpene moiety was established by HMBC and ^1^H–^1^H COSY NMR spectra analyses (Fig. 3). The occurrence of spin systems of H-2/H-6/H-5/H-4 in the ^1^H–^1^H COSY spectrum, suggested the presence of a cyclopentane unit. Meanwhile, the long-range correlations in the HMBC spectrum from H_3_-10 to C-2, C-3, and C-4, and from H_3_-9 to C-6, C-7, and C-8, determined the location of Me-10 and the linkage between the isoalkyl group and C-6. Ultimately, the connection of monoterpene and acylphloroglucinol motifs was demonstrated by the pivotal correlations of H-1 with C-2, C-3, C-6, C-2′, and C-3′ (HMBC), as well as H-1 with H-2 (^1^H–^1^H COSY), which also established a pyran ring between the aforementioned motifs to suit the seven degrees of unsaturation. Thus, compound 1 with a ring system of a cyclopenta[*b*]chromene was characterized, which is an unreported carbon skeleton discovered for natural product compounds.

**Fig. 3:**
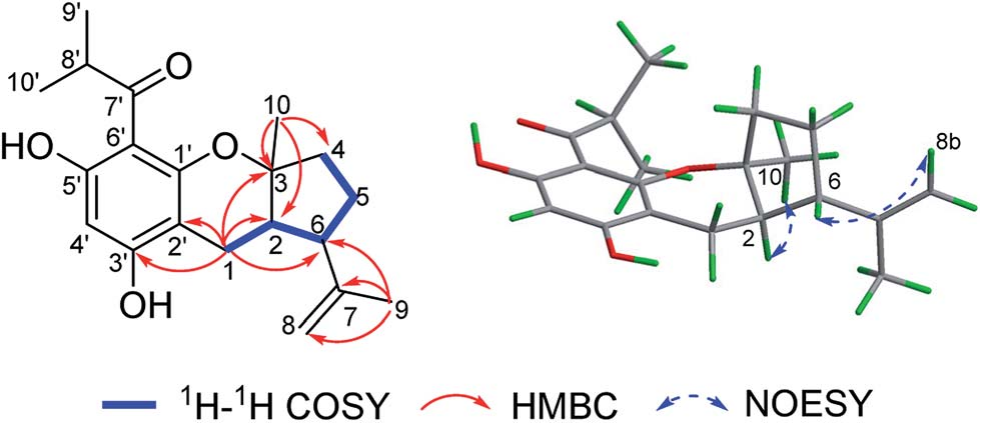
Key 2D NMR correlations of compound 1.

The relative configuration was tackled by performance of NOESY correlation analyses (Fig. 3). The NOE responses of H_3_-10/H-2 assigned these two protons to the same face in an *α*-orientation. Concurrently, a NOE interaction of H-6/H-8b (*δ*_H_ 4.80) was observed, but there was no NOE interaction between H-6 with H-2, suggesting the *β*-orientation of H-6 (Fig. 3).

**Fig. 4:**
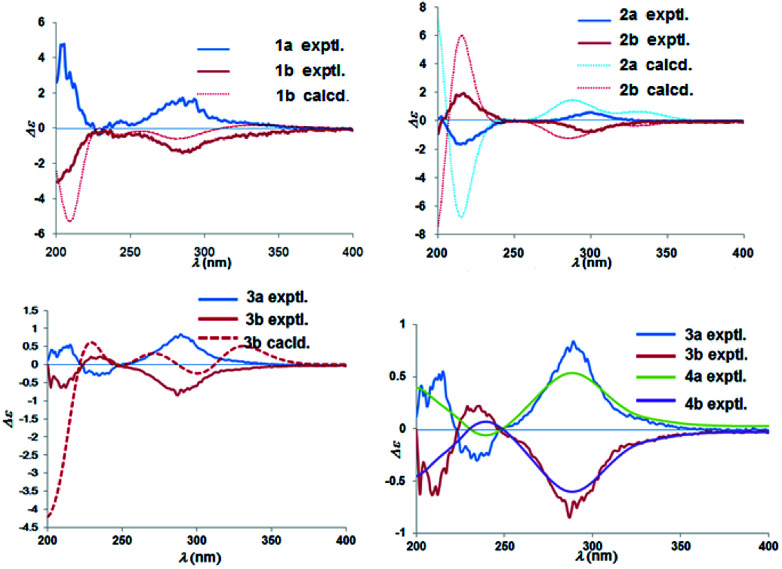
Experimental ECD spectra of 1a/1b–4a/4b and calculated ECD spectra of 1b, 2a/2b, and 3b.

Chiral separation of compound 1 (Fig. 2) gave rise to the pair of enantiomers 1a/1b, and their absolute stereochemistry were resolved unequivocally by means of a calculated ECD spectrum method (Fig. 4) as 2*R*,3*S*,6*S*, and 2*S*,3*R*,6*R*, respectively.

(±)-Japonicol F (2a/2b), a white amorphous powder, with the molecular formula of C_20_H_28_O_5_ (*m*/*z* 371.1834 [M + Na]^+^, calcd 371.1834), was separated into two enantiomers (Fig. 2), *viz.*2a and 2b. Detailed NMR data analyses manifested that the carbon resonances of C-4′, C-5′, and C-6′ were not observed; nevertheless, the presence of these carbons was deduced from the HRESIMS data. The monoterpenoid portion along with its linkage to the acylphloroglucinol group was elucidated based on the data of HMBC and ^1^H–^1^H COSY correlated resonances (ESI, Fig. S1†). HMBC correlations of H_3_-10 with C-2/C-3/C-4, and of H3-8/H3-9 with C-6/C-7, as well as ^1^H–1H COSY cross-peaks of H-4/H-5/H-6 resulted in an unambiguous assignment of the isopropenylmethyl-methyl unit, *viz.* C-3–C-10. The monoterpene entity is incorporated into the acylphloroglucinol unit to form a pyran ring, which was attributable to the HMBC correlations of H-1 with C-1′/C-2′/C-2/C-3 and the ^1^H–^1^H COSY cross-peak of H-1/H-2, accounting for the seven indices of hydrogen deficiency.

The relative configuration of 2 was designated through analyses of NOESY experiments. The NOE interactions of H-6 with H-5b (*δ*_H_ 2.16), H-5b with H-4b (*δ*_H_ 1.80), H-4b with H-1b (*δ*_H_ 2.85), and H-1b with H-2 suggested that these protons could be arbitrarily assigned as a *β* orientation. On the contrary, NOE correlations of H_3_-10 with H-5a (*δ*_H_ 2.20) and no NOE cross-peak between H_3_-10 with H-2 led to the reasonable assignment of the *α*-oriented position for H_3_-10.

**Fig. 5:**
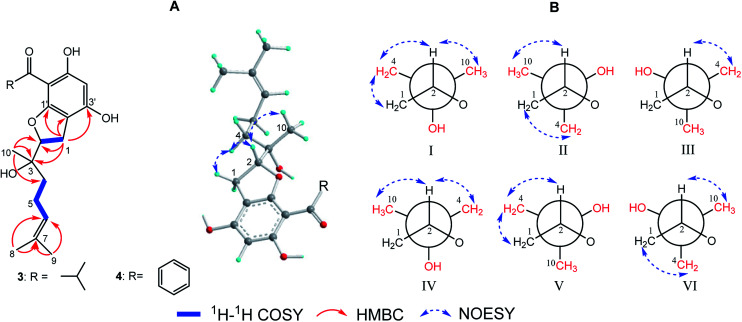
(A) Key 2D NMR correlations for the core structures of 3 and 4; (B) six preferential rotamers in Newman projections for C-2/C-3 of 3.

The absolute configuration of (±)-japonicol F (2a/2b), however, was eventually determined to be 2*R*,3*S* and 2*S*,3*R*, respectively, by the well-matched theoretical ECD Cotton effects curves with the experimental spectra (Fig. 4).

(±)-Japonicol G (3a/3b), separated by the analogue method (Fig. 2), has the identical molecular formula of japonicol F (2), C_20_H_28_O_5_, which was affirmed by the HRESIMS with a pseudomolecular ion peak at *m*/*z* 371.1814 [M + Na]^+^ (calcd 371.1834). Based on the inspection of the HMBC and ^1^H–^1^H COSY spectra, *viz.*, the long-range HMBC correlations of H_3_-10 to C-2/C-3/C-4, H3-8/H3-9 to C-6/C-7, and H-1 to C-2/C-3/C-1′/C-2′/C-3′, as well as the key COSY correlations of H-4/H-5/H-6, and H-1/H-2, the monoterpenoid moiety of 3 was unequivocally established as a geranyl unit, meanwhile accomplished with a furan ring to conjoin with acylphloroglucinol entity (Fig. 5A).

The side chain attached to C-2, *viz.*, the geranyl group, has free rotation and the absence of one stereogenic carbon to C-2. This made it impossible to confirm the relative configurations of C-2 and C-3 merely *via* the NOESY signals, for instance, of harronin I and II.^11^ The relative configurations of the two adjacent carbons C-2 and C-3, presented with six preferential rotamers of 3 are shown in Newman projections (Fig. 5B). The major rotamer of 3 should be I as shown in Fig. 5B, since its NOESY interactions acted in accordance with the results of NOESY experiments, *viz.*, H-2 with H-4/H-10, and H-1 with H-4 (more details showed in ESI†). Thus, the relative chiralities of C-2 and C-3 were deduced to be 2R*,3S*, which is consistent with the same type of stereogenic carbons in bonannione B and bonanniol D.^12^

The absolute stereochemistry of C-2 was established as 2*S* and 2*R* for 3a and 3b respectively, since the ECD spectra of 3a and 3b showed similar Cotton effects with ones of peperobtusin A (negative or positive Cotton effect around 230 nm, and positive or negative Cotton effect around 275 nm, comparatively).^13^ Consequently, the absolute configurations of (±)-japonicol G (3a/3b) were ascertained to be 2*S*,3*R*, and 2*R*,3*S*, respectively, which were also consistent with the matched ECD spectra between experimental and calculated ones (Fig. 4).

(±)-Japonicol H (4a/4b) was isolated as a reddish brown oil. The only structural difference between it and japonicol G was the presence of a phenyl ring instead of an isopropyl group in the latter at C-7′ of the acylphloroglucinol unit, which was inferred from HRESIMS data with a protonated molecular ion at *m*/*z* 383.1876 (calcd 383.1858). The enantiomeric separation procedure and 2D NMR correlations of 4 are presented in Fig. 2 and 5A, respectively. The relative configuration of 4 was confirmed due to its ^13^C NMR chemical shifts at C-2 and C-3 at *δ*_C_ 91.9 and *δ*_C_ 74.4, respectively, which were identical values to those presented of compound 3 (Table 1). In comparison with 3a/3b (Fig. 4), the homologous ECD Cotton curves showed that 4a/4b possessed a consistent intrinsic chirality.

Biological activities of these four pairs of enantiomers 1a/1b–4a/4b towards anti-KSHV activity were carried out by reported methods.^2*a*,6*c*^ Human iSLK.219 cells (derived from iSLK cells) inserted with rKSHV.219 [a sort of recombinant virus encoded with green fluorescent protein (GFP) and red fluorescent protein (RFP)], were utilized to assay the antiviral activity. The cytotoxicity of the compounds was measured by an AlamarBlue® Cell Viability Assay (Invitrogen), applying the protocol of the manufacturer, and was expressed as CC_50_ (the 50% cytotoxic concentration value). The inhibitory effects on KSHV lytic replication, assessed by infectivity assays through the GFP expression per well in Vero cells, were expressed as IC_50_ values (the 50% inhibiting concentration value). Effect of each compound demonstrated that 1a and 4a possessed encouraging inhibitory effects on KSHV lytic replication in Vero cells, with IC_50_ values of 8.30 and 4.90 μM, and selectivity indexes of 23.49 and 25.70, respectively (Table 2).

**Table 2:** Antci-KSHV activities of compounds 1a/1b–8a/8b (μM)

Compounds	CC_50_	IC_50_	Selective index (CC_50_/IC_50_)
Cidofovir^a^	>1.00	0.0081	>123.30
1a	195.00	8.30	23.49
1b	190.20	24.46	7.78
2a	126.00	28.00	4.50
2b	150.20	27.3	5.50
3a	79.50	21.73	3.66
3b	50.10	6.70	7.48
4a	125.90	4.90	25.70
4b	>200.00	29.46	>6.79
5a	>500.00	202.90	>2.46
5b	>500.00	140.90	>3.55
6a	140.60	8.75	16.06
6b	173.70	29.13	5.96
7a	221.10	17.67	12.51
7b	>300.00	39.80	>7.50
8a	>300.00	40.00	>7.50
8b	>300.00	158.50	>1.89

a Positive control.

For subsequent SAR studies of these enantiomers, the foregoing bioactivities data were used (Table 2). With a common scaffold of the acylphloroglucinol moiety, a variety of heterocycles were incorporated into the scaffold in the 1′ and 2′ positions by formation of pyran, furan, or oxepine rings. Generally, with only fused rings, addition of a remote vinyl group (1a/1b–4a/4b) caused a coarse-grained increase of IC_50_ when compared to 6a/6b–8a/8b with the vinyl group replaced by a tertiary hydroxyl group, suggesting that a polar group in that position is not tolerated. The occurrence of a bridged ring (5a/5b) caused a decrease of the IC_50_, which might indicated that the flat conformation of the fused ring plays a role. Notably, given the commonness of 3a, the enhanced activity and selectivity of 4a is likely attributable to the unique phenyl group at the 7′ position. Furthermore, it is noteworthy that the selectivity indices roughly correlate linearly with the IC_50_ values. However, they are entirely uncorrelated with the CC_50_ values.

**Fig. 6:**
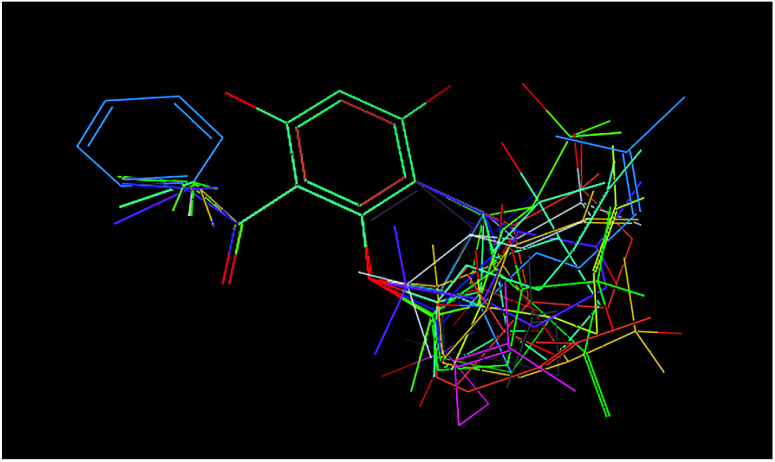
Ligands were aligned through flexible shape-based alignment, which was implemented in Schrödinger Suite.

**Fig. 7:**
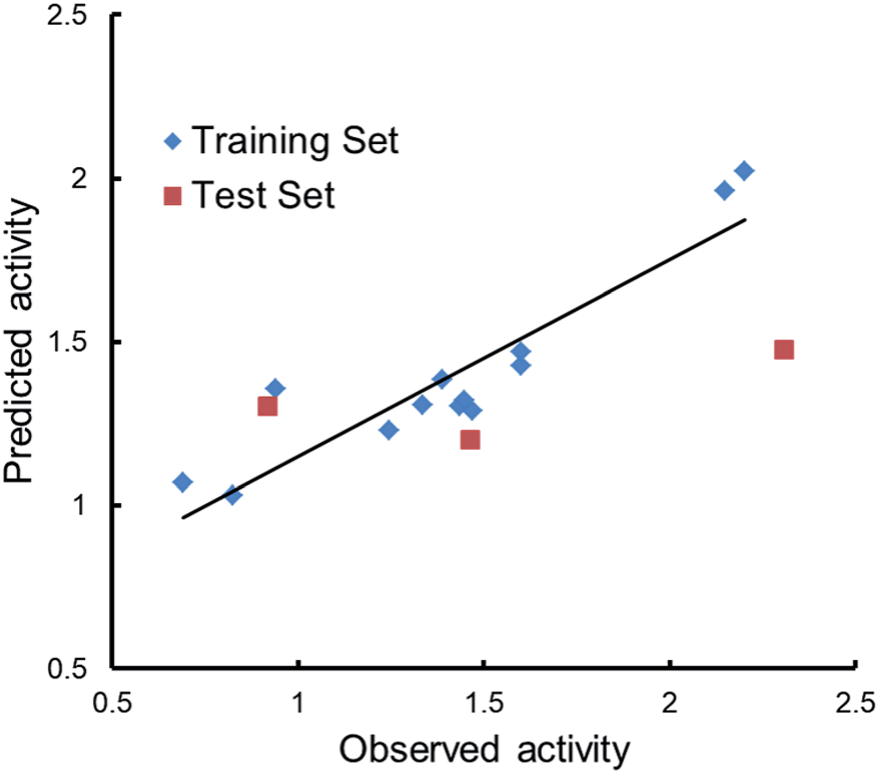
Scatter plots of observed *versus* predicted activities.

Qualitative analysis was followed by quantitative SAR (QSAR) modelling utilizing the Field-Based QSAR Panel implemented in Schrödinger Suite^14^ to set up CoMFA/CoMSIA models from these enantiomers. These enantiomers first superimposed using the flexible shape-based alignment method (Fig. 6). The training set was used to set up the model (Table 3), and the best model (Table 4) showed moderate predictive ability. Experimental activities of additional analogues would likely provide a better model. Moreover, correlation between the observed activities and the predicted activities was plotted in Fig. 7.

**Table 3:** Data set assignment, observed and predicted activity, and their residuals (prediction error) of training and test set

Ligand name	QSAR set	Observed activity	Predicted activity	Prediction error
4a	Training	0.690	1.070	−0.380
4b	Training	1.469	1.289	0.180
3b	Training	0.826	1.030	−0.204
6a	Training	1.247	1.228	0.019
3a	Training	1.337	1.305	0.032
2b	Training	1.436	1.302	0.135
2a	Training	1.447	1.319	0.128
1b	Training	1.388	1.384	0.005
8a	Training	1.602	1.426	0.176
7a	Training	0.942	1.355	−0.413
6b	Training	1.600	1.469	0.130
5b	Training	2.149	1.963	0.186
8b	Training	2.200	2.022	0.178
1a	Test	0.919	1.305	−0.385
7b	Test	1.464	1.201	0.263
5a	Test	2.307	1.477	0.831

**Table 4:** PLS statistics of field-based QSAR model^a^

Factors	SD_train_	*R*_train_^2^	*F*_train_	*p*_train_	RMSE_test_	*Q*_test_^2^	Pearson's *r*_test_
1	0.2861	0.6184	17.8	0.00143	0.54	0.0944	0.771
2^b^	0.1922	0.8434	26.9	9.42 × 10^−5^	0.55	0.072	0.7102

a Factors = number of PLS factors in the model; *R*^2^ = correlation coefficient of experimentally observed and predicted activity; SD = standard deviation of regression; *F* = variance ratio; *p* = statistical significance; *Q*^2^ = value of *Q*^2^; RMSE = root mean square error; Pearson's *r* = correlation coefficient of predicted and experimentally observed activity.

b Best model.

**Fig. 8:**
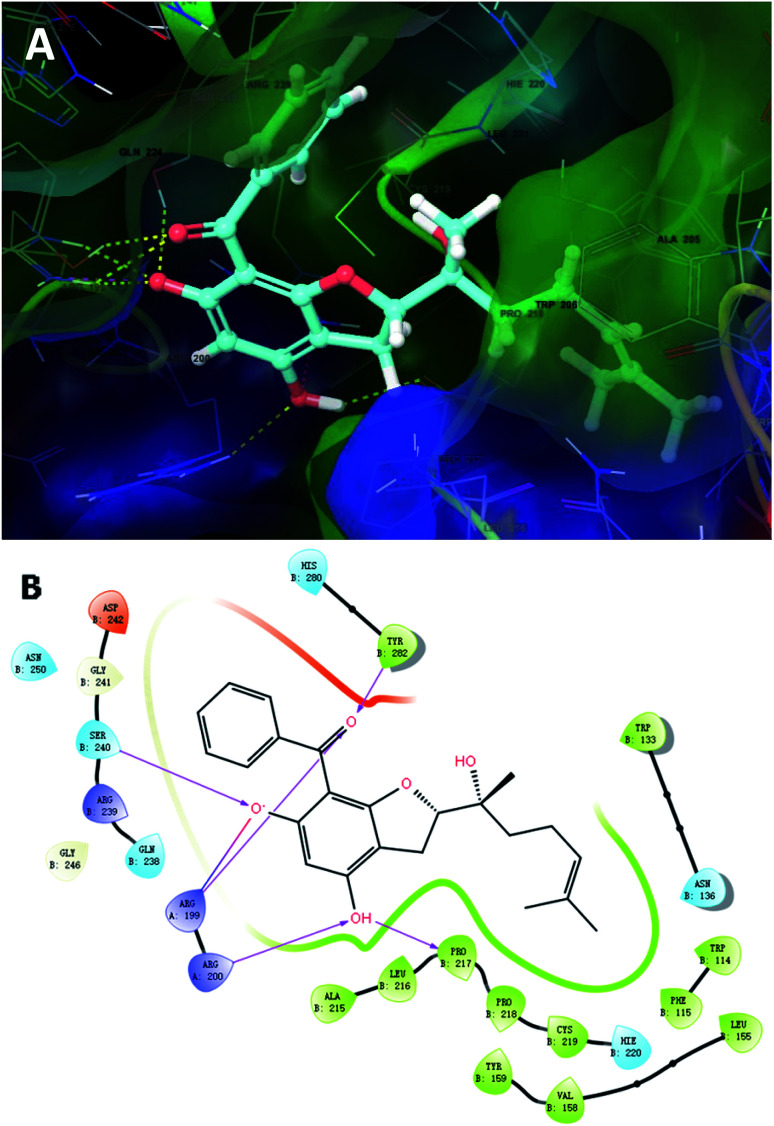
Induced fit docking of 4a to kTS (5H39): (A) binding pose; (B) 2D interaction diagram.

To further unravel the SAR of these enantiomers, a search for all complex assemblies relevant to KSHV lytic replication in the Protein Data Bank^15^ was performed, and two targets were found, namely Thymidylate Synthase (kTS) and protease (kPr). Crystal structures of kTS and kPr complexes (PDB ID: 5H39 (ref. 16) and 5V5D,^17^ respectively) were retrieved and 4a was docked into the pocket of associated reference ligands for each target using Glide. The Glide GScore of 4a docking to kTS was lower than that for kPr (Table 5), and thus, kTS was assumed to be a putative target for further Induced fit docking (additionally implemented in Suite). Induced fit docking allows the receptor to alter its binding site so that it more closely conforms to the shape and binding mode of the ligand, which gave a Glide GScore of −9.326 for 4a docking to kTS. As shown in Fig. 8, the acylphloroglucinol scaffold of 4a was anchored *via* hydrogen bonding to Arg199, Arg200, Pro217, Ser240 and Tyr282 with an additional salt bridge between 5′-OH and Arg199. Moreover, the remote vinyl group was inserted in a hydrophobic pocket formed by Phe115, Val158, Pro218 and other hydrophobic residues. In general, this predictive binding mode agreed roughly with the SAR previously discussed.

**Table 5:** Docking results for 4a to kPr and kTS at SP level of precision

Receptor	Glide GScore (kcal mol^−1^)
kPr (5V5D)	−6.035
kTS (5H39)	−8.033

## Conclusions

KSHV and its viral genes are potentially associated with human malignant neoplastic diseases. Additionally, immunoassays have shown that KSHV-related antibodies remain in individuals with AIDS.^18^ In this research, (±)-japonicols E–H (1a/1b–4a/4b) as racemic mixtures were studied. Compounds 1a/1b, possess an unreported ring system of a cyclopenta[*b*]chromene. Each pair of japonicols E–H was obtained with approximate 100% values of enantiomeric excess (ee) from *Hypericum japonicum*. (±)-Japonicols E–H (1a/1b–4a/4b), as well as (±)-japonicols A–D (5a/5b–8a/8b), were carried out the inhibition assay on KSHV lytic replication using previously developed procedures.^6*c*^ The inhibitory effects revealed that (+)-japonicols E (1a) and H (4a) presented promising anti-KSHV activity *in vitro* along with IC_50_ values of 8.30 and 4.90 μM and selectivity indexes of 23.49 and 25.70, respectively. These aspects might gain insight into an extensive research for developing analogous molecules for the medical treatment of diverse KSHV associated diseases.

These enantiomers contained a variety of heterocycles incorporated into the scaffold in the 1′ and 2′ positions, leading to difficulties in analysis of their structure–activity relationships. Notwithstanding, some general correlations with the variation of IC_50_ and selective indices were determined. Furthermore, to find a putative target and to predict its binding mode were undertaking using molecular docking, which represents the first example of Qualitative and quantitative structure–activity relationship (SAR) and molecular docking studies for acylphloroglucinol-based meroterpenoids as anti-KSHV agents. This study provides a useful starting point for further development and optimization of the anti-KSHV activities of these types of compounds and for the discovery of new therapeutic candidate drugs. Future work will include the development of synthetic routes to afford 4a and its analogues, and the investigation of the molecular mechanisms involved in the inhibitory effects of these types of compounds.

## Experimental section

### General experimental procedures

All isolated metabolites were carefully screened by analytical TLC plates (Merck, Germany) under ultraviolet-visible detector with *λ* 254 nm. Separations were performed by silica gel (200–300 mesh, Yantai Chemical Co., Ltd., China), ODS (YMC Co., Japan), and Sephadex LH-20 (Mitsubishi Chemical Co., Japan) column chromatography. Generally, all target products were purified through an analytical HPLC (Shimadzu LC-10AVP Plus) with a RP-C_18_ column (5 μm, 10 × 250 mm, Welchrom®, China) and a CHIRALPAK®IC column (5 μm, 10 × 250 mm, Daicel, China). HRESIMS data were acquired by a Thermo Scientific LTQ-Orbitrap XL apparatus. IR spectra were measured on a Bruker Vertex 70 spectrophotometer using KBr discs. UV spectra were recorded using a Varian Cary 50 instrument. Circular dichroism chiroptical spectra were performed using a JASCO J-1700 spectrometer. The ^1^H (400 MHz) and ^13^C (100 MHz) NMR spectra were obtained using a Bruker AM-600 spectrometer with tetramethylsilane (TMS) as an internal standard while the chemical shifts were characterized to solvent peaks (CD_3_OD, *δ*_H_ 3.31 ppm; *δ*_C_ 49.15 ppm).

### Plant material

The whole herbs of *H. japonicum* were harvested from the DaBie Mountain area of Hubei Province, P. R. China, in October 2011, and were authenticated by Professor Jianping Wang (School of Pharmacy, Tongji Medical College, Huazhong University of Science and Technology). A voucher specimen (ID 20111011) has been stockpiled in the Hubei Key Laboratory of Biotechnology of Chinese Traditional Medicine, Tongji Medical College, Huazhong University of Science and Technology.

### Extraction and isolation

The detailed procedures of the extraction and isolation are presented in ESI.†

#### (±)-Japonicol E (1a/1b)

UV (CH_3_OH) *λ*_max_ (log *ε*) 234 (3.53), 290 (3.68) nm; IR (KBr) *v*_max_ 3279, 2958, 1626, 1593, 1609, 1438 cm^−1^; ^1^H and ^13^C NMR data, see Table 1; HRESIMS *m*/*z* 331.1963 [M + H]^+^ (calcd for C_20_H_27_O_4_, 331.1909).

##### 1a [(+)-japonicol E]

White amorphous powder, [*α*] +82.5 (*c* 0.04, CH_3_OH); ECD (CH_3_OH) *λ*(Δ*ε*) 285 (+1.71) nm.

##### 1b [(−)-japonicol E]

White amorphous powder, [*α*] −83.3 (*c* 0.06, CH_3_OH); ECD (CH_3_OH) *λ*(Δ*ε*) 285 (−1.40) nm.

#### (±)-Japonicol F (2a/2b)

UV (CH_3_OH) *λ*_max_ (log *ε*) 292 (4.04), 345 (3.62) nm; IR (KBr) *v*_max_ 3318, 2922, 2853, 1618, 1509, 1421 cm^−1^; ^1^H and ^13^C NMR data, see Table 1; HRESIMS *m*/*z* 371.1834 [M + Na]^+^ (calcd for C_20_H_28_O_5_Na, 371.1834).

##### 2a [(+)-japonicol F]

White amorphous powder, [*α*] +14.5 (*c* 0.07, CH_3_OH); ECD (CH_3_OH) *λ*(Δ*ε*) 215 (−1.65), 300 (+0.60) nm.

##### 2b [(−)-japonicol F]

White amorphous powder, [*α*] −14.5 (*c* 0.05, CH_3_OH); ECD (CH_3_OH) *λ*(Δ*ε*) 217 (+1.93), 299 (−0.77) nm.

#### (±)-Japonicol G (3a/3b)

UV (CH_3_OH) *λ*_max_ (log *ε*) 293 (4.32) nm; IR (KBr) *v*_max_ 3295, 2970, 2931, 2872, 1633, 1609, 1601, 1428 cm^−1^; ^1^H and ^13^C NMR data, see Table 1; HRESIMS *m*/*z* 371.1814 [M + Na]^+^ (calcd for C_20_H_28_O_5_Na, 371.1834).

##### 3a [(+)-japonicol G]

White amorphous powder, [*α*] +94.2 (*c* 0.05, CH_3_OH); ECD (CH_3_OH) *λ*(Δ*ε*) 215 (+0.55), 234 (−0.30), 289 (+0.84) nm.

##### 3b [(−)-japonicol G]

White amorphous powder, [*α*] −93.4 (*c* 0.05, CH_3_OH); ECD (CH_3_OH) *λ*(Δ*ε*) 209 (−0.63), 235 (+0.22), 287 (−0.85) nm.

#### (±)-Japonicol H (4a/4b)

UV (CH_3_OH) *λ*_max_ (log *ε*) 293 (4.32) nm; IR (KBr) *v*_max_ 3243, 2970, 2859, 1645, 1566, 1446 cm^−1^; ^1^H and ^13^C NMR data, see Table 1; HRESIMS *m*/*z* 383.1876 [M + H]^+^ (calcd for C_23_H_27_O_5_, 383.1858).

##### 4a [(+)-japonicol H]

Colorless oil, [*α*] +87.0 (*c* 0.05, CH_3_OH). ECD (CH_3_OH) *λ*(Δ*ε*) 239 (−0.06), 288 (+0.54) nm.

##### 4b [(−)-japonicol H]

Colorless oil, [*α*] −89.2 (*c* 0.05, CH_3_OH). ECD (CH_3_OH) *λ*(Δ*ε*) 240 (+0.07), 288 (−0.60) nm.

### Anti-KSHV assay

Human iSLK.219 cells (derived from iSLK cells) and Vero cells were cultivated in Dulbecco's Modified Eagle's Medium (DMEM) supplemented with 10% fetal bovine serum (Invitrogen, Carlsbad, CA, USA) in humidified incubators under 5% CO_2_ at 37 °C. The rKSHV.219 recombinant viruses were inserted in iSLK.219 cells with a doxycycline-inducible RTA expression system.^19^ The green fluorescent protein (GFP) regulated by the EF-1α promoter indicated the existence of the virus, while the red fluorescent protein (RFP) regulated by the virus lytic PAN promoter manifested the occurrence of virus lytic replication.^2*b*^

The CC_50_ for test compounds was executed *via* a previously-developed procedure.^2*b*,19^ The iSLK.219 cells were incubated in 96-well plates (100 μL per well). Test compounds were diluted to gradient concentration using the culture medium with doxycycline (Dox) (Beyotime) and sodium butyrate (NaB) (Sigma). The cells were then treated with the indicated compounds and cultured under 5% CO_2_ at 37 °C for 48 h. Cells without compound treatment were employed as the control. Subsequently, survival cells were assessed *via* AlamarBlue® Cell Viability Assay (Invitrogen). The CC_50_ values of the compounds were evaluated by the formula: cytoactive rate (%) = (*V*_treated_ − *V*_blank_)/(*V*_control_ − *V*_blank_) × 100%. The values were determined by Graphpad5.0 Prism software based on the mean ± standard deviation (*n* = 3) (ESI, Fig. S2†).

The inhibitory effects on KSHV lytic replication of the test compounds were expressed as IC_50_ values, which were assessed by infectivity assays according to a reported method.^2*a*^ In brief, iSLK.219 cells were seeded in the 96-well plates until growth to 80% cell confluency. They were then treated for 48 h using various concentrations of compounds with the inducer (Dox and NaB). Next, the supernatants of iSLK.219-treated or -untreated with the compounds were harvested after centrifugation to infect Vero cells for 48 h. Cells without compound treatment were employed as the control, and the group without the inducer was used as the reference of the virus lytic replication. The GFP expression per well in Vero cells was analyzed through the Operetta High-Content Screening System (HCS) (Perkin Elmer), and the data of the GFP intensity per well was determined by the Harmony 3.5 software (Perkin Elmer). The IC_50_ values were obtained by the formula: inhibition rate (%) = (*V*_control_ − *V*_treated_)/*V*_control_ × 100%. The values were determined by mean ± standard deviation (*n* = 3) (ESI, Fig. S2†).

### Computational ECD methodology

The calculated ECD spectrum were performed for each conformer of compounds 1b, 2a/2b, and 3b using the TDDFT methodology at the B3LYP/6-311++G(d,p)//B3LYP/6-31G(d) level with MeOH as solvent by the IEFPCM solvation model implemented in Gaussian 09 program. More details are showed in ESI.†

### Computational QSAR methods

QSAR studies were conducted with Schrödinger Suite 2017-1.

### Field-based 3D QSAR

A data set (Table 3) was randomly assigned into training and test sets with a ratio of approximately 4/1, and their activities (IC_50_) were converted to the logarithm (log IC_50_). The minimized conformations of these enantiomers were generated utilizing LigPrep and aligned according to the flexible shape-based alignment method. The force field was used and the maximum PLS factors were set as 1/5 of the total number of the training set in order to avoid over-fitting. The parameters that were not mentioned were left as defaults.

### Molecular docking

Binding patterns were characterized through molecular docking implemented by Glide and Induced Fit modules at the SP level of precision. The retrieved crystal structures were prepared by Protein Preparation Wizard for assigning bond orders, adding hydrogens, protonating and restraining minimizing. The best conformation of each ligand was ranked by Glide Emodel. The parameters that were not mentioned were left as defaults.

## Conflicts of interest

The authors declare no competing financial interest.

## Supplementary Material

RA-008-C8RA04073G-s001
