# Supplementary material for: Discovery of acylphloroglucinol-based meroterpenoid enantiomers as KSHV inhibitors from Hypericum japonicum
Source: RSC Adv. 2018 Jul 2;8(43):24101–9. doi: 10.1039/c8ra04073g (PMC9081833; doi:10.1039/c8ra04073g)

Electronic Supplementary Material (ESI) for RSC Advances.
This journal is © The Royal Society of Chemistry 2018

# Discovery of Acylphloroglucinol-based Meroterpenoid Enantiomers as KSHV Inhibitors from *Hypericum japonicum*

Linzhen Hu, †[a,b] Yanfei Liu, †[c] Yanxing Wang,[d] Zhenzhen Wang,[b] Jinfeng Huang,[b] Yongbo Xue,[b] Junjun Liu,[b] Zhenming Liu, *[c] Yong Chen*[a] and Yonghui Zhang*[b]

[a] *National & Local Joint Engineering Research Center of High-throughput Drug Screening Technology, Hubei Key Laboratory of Biotechnology of Chinese Traditional Medicine, School of Life Sciences, Hubei University, Wuhan 430062, Hubei Province, P. R. China*

[b] *Hubei Key Laboratory of Natural Medicinal Chemistry and Resource Evaluation, Tongji Medical College, Huazhong University of Science and Technology, Wuhan 430030, Hubei Province, P. R. China*

[c] *The Central Hospital of Wuhan, Huazhong University of Science and Technology, Wuhan 430014, Hubei Province, P. R. China*

[d] *State Key Laboratory of Natural and Biomimetic Drugs, School of Pharmaceutical Sciences, Peking University, Beijing 100191, P. R. China*

Corresponding Authors

* Y. Zhang: e-mail zhangyh@mails.tjmu.edu.cn; fax 86-27-83692762; tel 86-027-83692892.

* Y. Chen: e-mail cy101610@qq.com; fax 86-27-88663590.

* Z. Liu: e-mail zmliu@bjmu.edu.cn.

† These authors contributed equally to this work.

# CONTENTS

**Figure S1**. Key 2D NMR correlations of **2**. ........................................................................................1

**Figure S2**. Effects on Human iSLK.219 cells viabilities and inhibition on lytic replication of KSHV infecting Vero cells of **1a**/**1b**−**4a**/**4b** were measured using CDV as positive control *in vitro*. ........................................................................................1

**Extraction and Isolation** ........................................................................................2

**Computational ECD details** ........................................................................................3

HRESIMS of compound **1** ........................................................................................45

$^{1}$H NMR of compound **1** (in methanol-$d_4$)........................................................................................46

$^{13}$C NMR of compound **1** (in methanol-$d_4$)........................................................................................47

HSQC of compound **1** (in methanol-$d_4$) ........................................................................................48

HMBC of compound **1** (in methanol-$d_4$) ........................................................................................49

$^{1}$H–$^{1}$H COSY of compound **1** (in methanol-$d_4$)........................................................................................50

NOESY of compound **1** (in methanol-$d_4$)........................................................................................51

UV of compound **1** (in MeOH)........................................................................................52

IR of compound **1** (KBr disc) ........................................................................................53

HRESIMS of compound **2** ........................................................................................54

$^{1}$H NMR of compound **2** (in methanol-$d_4$)........................................................................................55

$^{13}$C NMR of compound **2** (in methanol-$d_4$)........................................................................................56

HSQC of compound **2** (in methanol-$d_4$) ........................................................................................58

HMBC of compound **2** (in methanol-$d_4$) ........................................................................................59

$^{1}$H–$^{1}$H COSY of compound **2** (in methanol-$d_4$)........................................................................................60

NOESY of compound **2** (in methanol-$d_4$)......61

UV of compound **2**(in MeOH)......62

IR of compound **2**(KBr disc)......63

HRESIMS of compound **3**......64

$^1$H NMR of compound **3** (in methanol-$d_4$)......65

$^{13}$C NMR of compound **3** (in methanol-$d_4$)......67

HSQC of compound **3** (in methanol-$d_4$)......68

HMBC of compound **3** (in methanol-$d_4$)......69

$^1$H–$^1$H COSY of compound **3** (in methanol-$d_4$)......70

NOESY of compound **3** (in methanol-$d_4$)......71

UV of compound **3**(in MeOH)......72

IR of compound **3** (KBr disc)......73

HRESIMS of compound **4**......74

$^1$H NMR of compound **4** (in methanol-$d_4$)......75

$^{13}$C NMR of compound **4** (in methanol-$d_4$)......76

HSQC of compound **4** (in methanol-$d_4$)......78

HMBC of compound **4** (in methanol-$d_4$)......79

$^1$H–$^1$H COSY of compound **4** (in methanol-$d_4$)......80

NOESY of compound **4** (in methanol-$d_4$)......81

UV of compound **4** (in MeOH)......82

IR of compound **4** (KBr disc)......83

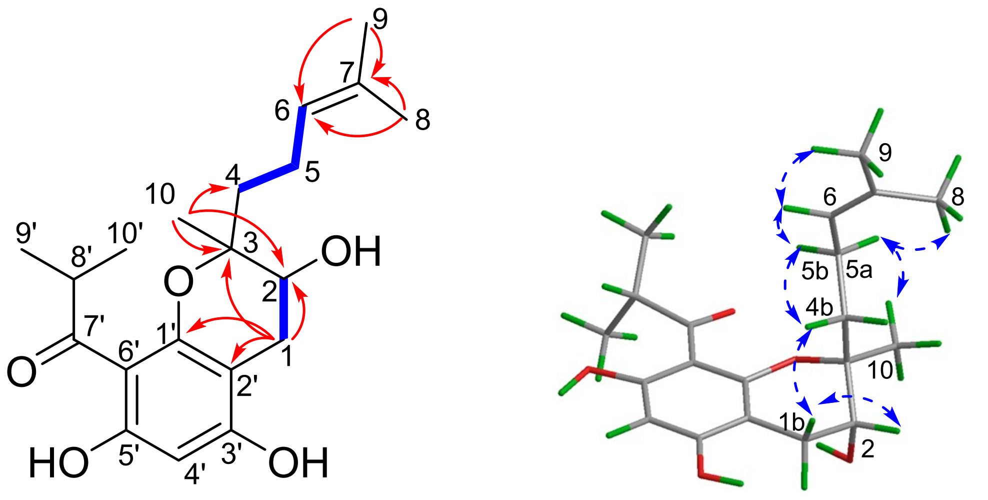


2 2

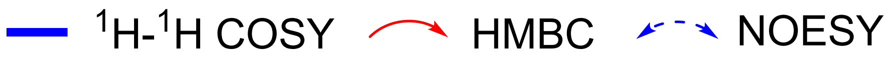


**Figure S1**. Key 2D NMR correlations of **2**.

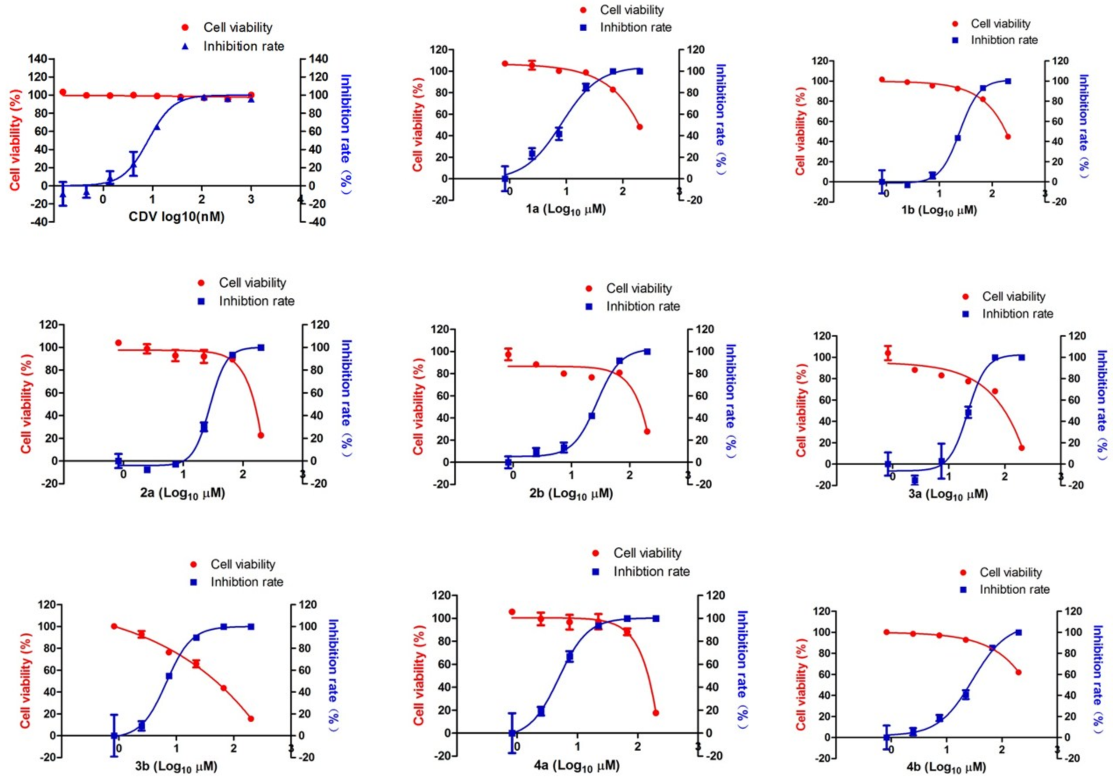


**Figure S2**. Effects on Human iSLK.219 cells viabilities and inhibition on lytic replication of KSHV infecting Vero cells of **1a**/**1b**–**4a**/**4b** were measured using CDV as positive control *in vitro*.

**Extraction and Isolation**

The whole herbs of *H. japonicum* (30 kg) were soaked in 95% EtOH at 25 °C and yielded a crude extract (0.75 kg) after removed the solvent under vacuum. The crude extract was suspended in water and sequentially partitioned with petroleum ether, chloroform, and ethyl acetate. The petroleum ether-soluble extract (400 g) was subsequently partitioned by silica gel column chromatography (3 kg, 20 × 120 cm; petroleum ether-acetone, 50:1→5:1) to afford seven fractions (Fr.1–Fr.7). Fr.6 was repeatedly chromatographed using silica gel CC (0.6 kg, 10 × 100 cm), eluting with a gradient of petroleum ether-EtoAc (30:1→3:1) to afford six subfractions (Fr.6.1–Fr.6.6). Fr.6.4 was subjected to MPLC (ODS, 1.5 × 20 cm, MeOH-$H_2O$, 30–90%) to obtain subfractions Fr.6.4.1–Fr.6.4.6. Fr.6.4.3 was partitioned by Sephadex LH-20 (2 × 150 cm, MeOH) and purified via semi-preparative HPLC ($CH_3CN/H_2O$ 55:45). Finally, a second semi-preparative HPLC (MeOH/$H_2O$ 53:47) was performed to yield three pairs of racemates, namely, **1** (4.00 mg), **2** (7.00 mg), and **3** (11.02 mg). Fr.6.4.2 was repurified by silica gel CC ($CHCl_3$/MeOH 40:1), Sephadex LH-20 (2 × 150 cm, $CH_2Cl_2$-MeOH, 1:1), and semi-preparative HPLC ($CH_3CN/H_2O$ 70:30) to afford the pair of racemates **4** (3.51 mg).

**Computational ECD details**

Conformational analyses were performed whereby both BALLOON and confab programs[1,2] in order to confirm the stereochemistry structure of compounds **1b**, **2a**/**2b**, **and 3b**. The BALLOON program explores conformational spaces with genetic algorithm, and synchronously, the confab program systematically generates diverse low energy conformations that are proposed to be close to crystal structures. The conformations generated by the above programs were assembled together via the removal of duplicated conformations whose root mean square (RMS) distance was less than 0.5 Å. Semi-empirical PM3 quantum mechanical geometry optimizations were fulfilled on conformations through the Gaussian 09 program.[3] Duplicated conformations after geometry optimization were subsequently identified and disposed. Remaining conformations were further optimized at B3LYP/6-31G* level of theory in methanol solvent with IEFPCM3 solvation model using Gaussian 09 program,[4] and duplicated conformations emerging after these calculations were removed according to the same RMS criteria above. Harmonic vibrational frequencies were performed to establish the stability of the finally obtained conformers. Oscillator strengths and rotational strengths of 20 weakest electronic excitations of each conformer were calculated by the TDDFT methodology at the B3LYP/6-311++G** level of theory adopting methanol as solvent by the IEFPCM solvation model carried out in Gaussian 09 program. The ECD spectra data for each conformer were then simulated by using a Gaussian function with a bandwidth $\sigma$ of 0.45 eV. Calculated spectra for each conformation were combined after Boltzmann weighting according to their population contribution.


(1) Vainio, M. J., Johnson, M. S., **2007**. Generating conformer ensembles using a multiobjective genetic algorithm. *J. Chem. Inf. Model*. *47*, 2462–2474.

(2) O′Boyle, N. M., Vandermeersch, T., Flynn, C. J., Maguire, A. R., Hutchison, G. R., **2011**. Confab-systematic generation of diverse low-energy conformers. *J. Cheminformatics 3*, 1–9.

(3) Frisch, M. J., Trucks, G. W., Schlegel, H. B., Scuseria, G. E., Robb, M. A., Cheeseman, J. R., Scalmani, G., Barone, V., Mennucci, B., Petersson, G. A., Nakatsuji, H., Caricato, M., Li, X., Hratchian, H. P., Izmaylov, A. F., Bloino, J., Zheng, G., Sonnenberg, J. L., Hada, M., Ehara, M., Toyota, K., Fukuda, R., Hasegawa, J., Ishida, M., Nakajima, T., Honda, Y., Kitao, O., Nakai, H., Vreven, T., Montgomery, J. A., Peralta, J. E., Ogliaro, F., Bearpark, M., Heyd, J. J., Brothers, E., Kudin, K .N., Staroverov, V. N., Keith, T., Kobayashi, R., Normand, J., Raghavachari, K., Rendell, A., Burant, J. C., Iyengar, S. S., Tomasi, J., Cossi, M., Rega, N., Millam, J. M., Klene, M., Knox, J. E., Cross, J. B., Bakken, V., Adamo, C., Jaramillo, J., Gomperts, R., Stratmann, R. E., Yazyev, O., Austin, A. J., Cammi, R., Pomelli, C., Ochterski, J. W., Martin, R. L., Morokuma, K., Zakrzewski, V. G., Voth, G. A., Salvador, P., Dannenberg, J. J., Dapprich, S., Daniels, A. D., Farkas, O., Foresman, J. B., Ortiz, J. V., Cioslowski, J., Fox, D. J. Gaussian, Inc., Wallingford CT, 2010.

(4) Tomasi, J., Mennucci, B., Cammi, R., 2005. Quantum mechanical continuum solvation models. Chem. Rev. 105, 2999–3094.

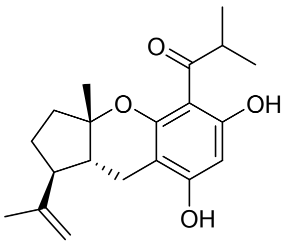

**1b**

Optimized geometries of predominant conformers for compound **1b** at the B3LYP/6-31G (d,p) level in methanol solution.

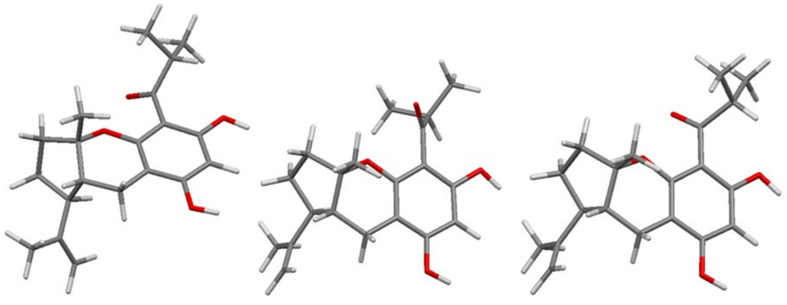

**1b** (1) **1b** (2) **1b** (3)

Important thermodynamic parameters (a.u.) and Boltzmann distributions of the optimized compound **1b** at B3LYP/6-31G(d,p) level in methanol solution.

| Conformations | E+ZPE | G | % |
|---|---|---|---|
| **1b (1)** | -1078.253342 | -1078.307663 | 75.62% |
| **1b (2)** | -1078.252215 | -1078.305855 | 11.15% |
| **1b (3)** | -1078.252391 | -1078.306017 | 13.23% |

E+ZPE, G: total energy with zero point energy (ZPE) and Gibbs free energy in methanol solution at B3LYP/6-31G (d,p) level. %: Boltzmann distributions, using the relative Gibbs free energies as weighting factors.

Optimized Z-matrixes of compound **1b** in methanol solution (Å) at B3LYP/6-31G (d,p) level.

| **1b** (1) | | | | **1b** (2) | | | |
|---|---|---|---|---|---|---|---|
| C | -1.489183 | 2.856428 | 0.113342 | C | -1.578917 | 2.833843 | -0.354931 |
| C | -1.775115 | 0.453837 | -0.235691 | C | -1.844508 | 0.456438 | 0.141581 |
| C | 0.374153 | 1.341035 | 0.593465 | C | 0.356313 | 1.540545 | 0.408164 |
| C | -0.436027 | 0.268815 | 0.196848 | C | -0.473094 | 0.415266 | 0.496404 |
| C | -2.272325 | 1.767968 | -0.272379 | C | -2.37154 | 1.687218 | -0.284564 |
| C | -0.180955 | 2.62989 | 0.543522 | C | -0.228777 | 2.746039 | -0.011826 |
| C | 4.146206 | 0.920343 | -2.13253 | C | 4.814028 | 0.456418 | -0.806019 |
| C | -2.617148 | -0.706232 | -0.669694 | C | -2.714321 | -0.759572 | 0.26191 |
| C | 4.058964 | 0.074203 | -1.09753 | C | 3.82026 | -0.287445 | -1.307265 |
| C | 1.796192 | 1.131681 | 1.06228 | C | 1.828868 | 1.468037 | 0.741541 |
| C | 2.633817 | -2.087147 | -0.875478 | C | 2.516552 | -2.207659 | -0.199881 |
| C | 1.776276 | -2.60081 | 0.311149 | C | 1.81427 | -2.316067 | 1.179452 |

| | | | | | | | |
|---|---|---|---|---|---|---|---|
| C | 2.725196 | -0.535692 | -0.71027 | C | 2.572902 | -0.679411 | -0.537597 |
| C | 2.289647 | -0.284905 | 0.759041 | C | 2.323491 | 0.019167 | 0.817777 |
| C | 1.207321 | -1.350263 | 0.995672 | C | 1.309521 | -0.905626 | 1.513469 |
| C | 5.28279 | -0.292273 | -0.288327 | C | 3.882323 | -0.816443 | -2.722287 |
| C | 0.792141 | -1.552554 | 2.450105 | C | 1.094549 | -0.642588 | 3.000945 |
| C | -3.872313 | -2.054235 | 0.992679 | C | -4.927891 | -1.631077 | -0.608239 |
| C | -5.068208 | -1.191517 | -1.080522 | C | -2.711891 | -2.39487 | -1.601175 |
| C | -3.995245 | -0.898668 | -0.023143 | C | -3.495578 | -1.221629 | -0.973961 |
| O | -2.196941 | -1.535618 | -1.467878 | O | -2.751472 | -1.417933 | 1.294259 |
| O | 0.01261 | -1.015677 | 0.219074 | O | 0.014121 | -0.808471 | 0.839987 |
| O | -3.543582 | 1.945695 | -0.743473 | O | -3.707834 | 1.732003 | -0.572458 |
| O | 0.632092 | 3.652949 | 0.934913 | O | 0.595891 | 3.831514 | -0.067909 |
| H | -1.887937 | 3.866948 | 0.062082 | H | -2.012106 | 3.78526 | -0.65467 |
| H | 5.094021 | 1.360763 | -2.434223 | H | 5.693032 | 0.691762 | -1.401726 |
| H | 3.27292 | 1.20007 | -2.717659 | H | 4.800234 | 0.85789 | 0.202816 |
| H | 2.45682 | 1.865414 | 0.584609 | H | 2.407629 | 2.023017 | -0.004952 |
| H | 1.866202 | 1.329068 | 2.141024 | H | 2.01943 | 1.974467 | 1.698511 |
| H | 3.625671 | -2.549686 | -0.860024 | H | 3.529208 | -2.621515 | -0.147198 |
| H | 2.186612 | -2.337381 | -1.842057 | H | 1.98313 | -2.764495 | -0.975672 |
| H | 0.967242 | -3.270249 | 0.003526 | H | 0.984417 | -3.029137 | 1.188531 |
| H | 2.395662 | -3.145333 | 1.03236 | H | 2.523294 | -2.629782 | 1.953002 |
| H | 1.963759 | -0.08419 | -1.356425 | H | 1.713923 | -0.454113 | -1.185992 |
| H | 3.12672 | -0.524205 | 1.426066 | H | 3.244926 | -0.016856 | 1.411935 |
| H | 5.213839 | 0.085298 | 0.740154 | H | 3.837434 | -1.913448 | -2.740573 |
| H | 5.413662 | -1.379056 | -0.212808 | H | 3.025541 | -0.460563 | -3.310734 |
| H | 6.189796 | 0.126004 | -0.73494 | H | 4.800013 | -0.505736 | -3.230984 |
| H | 0.342191 | -0.644107 | 2.864788 | H | 0.691515 | 0.361202 | 3.172685 |
| H | 1.661276 | -1.814229 | 3.063076 | H | 2.040748 | -0.729384 | 3.545564 |
| H | 0.058309 | -2.362067 | 2.52689 | H | 0.386868 | -1.368094 | 3.415684 |
| H | -3.599201 | -2.985155 | 0.483855 | H | -4.920998 | -2.415907 | 0.154874 |
| H | -4.827765 | -2.208207 | 1.506398 | H | -5.455065 | -2.010981 | -1.490543 |
| H | -3.109753 | -1.844348 | 1.751839 | H | -5.491759 | -0.777477 | -0.215936 |
| H | -4.792434 | -2.068459 | -1.675212 | H | -2.657032 | -3.237369 | -0.902929 |
| H | -5.19214 | -0.341221 | -1.759971 | H | -1.68858 | -2.101854 | -1.862931 |
| H | -6.033999 | -1.388647 | -0.601722 | H | -3.210913 | -2.735533 | -2.515128 |
| H | -4.263243 | 0.011534 | 0.519221 | H | -3.527104 | -0.403135 | -1.697657 |
| H | -3.777516 | 2.886927 | -0.692167 | H | -3.943016 | 2.623684 | -0.877417 |
| H | 0.158752 | 4.496294 | 0.845016 | H | 0.100229 | 4.597709 | -0.400572 |
| **1b** (3) | | | | | | | |
| C | -1.474666 | 2.864786 | -0.055644 | | | | |
| C | -1.755525 | 0.445238 | -0.267217 | | | | |
| C | 0.372415 | 1.380947 | 0.564882 | | | | |
| C | -0.428443 | 0.286966 | 0.21207 | | | | |

| | | | | | | | |
|---|---|---|---|---|---|---|---|
| C | -2.249011 | 1.755337 | -0.396407 | | | | |
| C | -0.178954 | 2.664415 | 0.422647 | | | | |
| C | 4.987548 | 0.548235 | -0.18498 | | | | |
| C | -2.587725 | -0.738865 | -0.650955 | | | | |
| C | 4.102507 | -0.035267 | -1.002425 | | | | |
| C | 1.780694 | 1.20065 | 1.083602 | | | | |
| C | 2.66663 | -2.137004 | -0.630804 | | | | |
| C | 1.77395 | -2.570634 | 0.56117 | | | | |
| C | 2.755045 | -0.574558 | -0.559847 | | | | |
| C | 2.285989 | -0.230174 | 0.871843 | | | | |
| C | 1.194407 | -1.277479 | 1.150324 | | | | |
| C | 4.400283 | -0.211025 | -2.474211 | | | | |
| C | 0.744947 | -1.386058 | 2.604453 | | | | |
| C | -3.921218 | -1.986452 | 1.029985 | | | | |
| C | -5.02943 | -1.213568 | -1.125442 | | | | |
| C | -3.990601 | -0.881813 | -0.045277 | | | | |
| O | -2.143887 | -1.62374 | -1.373623 | | | | |
| O | 0.018465 | -0.992939 | 0.325855 | | | | |
| O | -3.50619 | 1.905693 | -0.912424 | | | | |
| O | 0.624322 | 3.709537 | 0.773717 | | | | |
| H | -1.869726 | 3.870577 | -0.178362 | | | | |
| H | 5.945666 | 0.903021 | -0.557731 | | | | |
| H | 4.803028 | 0.697642 | 0.874614 | | | | |
| H | 2.452778 | 1.911967 | 0.590787 | | | | |
| H | 1.818379 | 1.452699 | 2.152851 | | | | |
| H | 3.664872 | -2.578986 | -0.543235 | | | | |
| H | 2.253849 | -2.464843 | -1.589098 | | | | |
| H | 0.96873 | -3.253415 | 0.273629 | | | | |
| H | 2.369754 | -3.07284 | 1.330973 | | | | |
| H | 2.006692 | -0.171664 | -1.257369 | | | | |
| H | 3.105041 | -0.431897 | 1.573072 | | | | |
| H | 4.373749 | -1.270096 | -2.762535 | | | | |
| H | 3.645057 | 0.297264 | -3.089233 | | | | |
| H | 5.383769 | 0.189053 | -2.739207 | | | | |
| H | 0.287784 | -0.452904 | 2.950504 | | | | |
| H | 1.59903 | -1.609542 | 3.252703 | | | | |
| H | 0.007363 | -2.188179 | 2.715334 | | | | |
| H | -3.641829 | -2.944361 | 0.578092 | | | | |
| H | -4.897003 | -2.103775 | 1.514409 | | | | |
| H | -3.184499 | -1.747784 | 1.806017 | | | | |
| H | -4.747386 | -2.124537 | -1.663432 | | | | |
| H | -5.114714 | -0.398071 | -1.851792 | | | | |
| H | -6.014829 | -1.371414 | -0.672712 | | | | |

| | | | | | | | |
|---|---|---|---|---|---|---|---|
| H | -4.265647 | 0.057566 | 0.440636 | | | | |
| H | -3.738806 | 2.848537 | -0.924181 | | | | |
| H | 0.154171 | 4.5455 | 0.620466 | | | | |

Key transitions, oscillator strengths, and rotatory strengths in the ECD of conformers **1a (1)** at B3LYP /6-311++G(d,p) level.

| Species | Excited State | $\Delta E(eV)^a$ | $\lambda(nm)^b$ | $f^c$ | $R_{vel}^d$ |
|---|---|---|---|---|---|
| **1b (1)** | 88 -> 90 | 4.0199 | 308.43 | 0.017 | -59.9879 |
| | 86 -> 90 | 4.0793 | 303.93 | 0.0317 | 19.7563 |
| | 89 -> 90 | 4.3257 | 286.62 | 0.1875 | 55.8722 |
| | 86 -> 90 | 5.0648 | 244.79 | 0.0005 | -1.0552 |
| | 89 -> 94 | 5.152 | 240.65 | 0.0046 | 4.9622 |
| | 89 -> 92 | 5.2619 | 235.63 | 0.0336 | -6.8968 |
| | 88 -> 94 | 5.2929 | 234.24 | 0.0044 | -2.5732 |
| | 89 -> 97 | 5.4901 | 225.83 | 0.0039 | 2.329 |
| | 89 -> 93 | 5.5531 | 223.27 | 0.0326 | -13.8888 |
| | 89 -> 94 | 5.5813 | 222.14 | 0.0527 | -6.6385 |
| | 88 -> 92 | 5.6373 | 219.93 | 0.0116 | 0.0207 |
| | 89 -> 96 | 5.6975 | 217.61 | 0.0223 | -2.2737 |
| | 88 -> 93 | 5.724 | 216.6 | 0.0078 | 7.6239 |
| | 88 -> 97 | 5.7909 | 214.1 | 0.0013 | 3.5698 |
| | 88 -> 93 | 5.8259 | 212.81 | 0.0066 | 4.6942 |
| | 89 -> 98 | 5.8607 | 211.55 | 0.031 | 11.1585 |
| | 85 -> 90 | 5.9204 | 209.42 | 0.0045 | 21.5932 |
| | 88 -> 97 | 5.9321 | 209.01 | 0.0014 | 6.0943 |
| | 88 -> 97 | 5.9329 | 208.98 | 0.0293 | -31.7962 |
| | 88 -> 97 | 5.9731 | 207.57 | 0.0017 | 3.7673 |
| | 88 -> 98 | 6.0005 | 206.62 | 0.0629 | -25.1477 |
| | 86 -> 93 | 6.0342 | 205.47 | 0.0053 | -1.7579 |
| | 89 -> 99 | 6.0538 | 204.8 | 0.0154 | 6.932 |
| | 88 -> 92 | 6.0926 | 203.5 | 0.0595 | -12.2155 |
| | 89 -> 99 | 6.161 | 201.24 | 0.1501 | 87.088 |
| | 86 -> 92 | 6.1811 | 200.59 | 0.0271 | 9.9999 |
| | 87 -> 92 | 6.1844 | 200.48 | 0.0571 | -19.4122 |
| | 84 -> 9 | 6.2138 | 199.53 | 0.0577 | -115.5774 |
| | 88 ->100 | 6.2225 | 199.25 | 0.1151 | -25.2682 |
| | 87 -> 91 | 6.2384 | 198.74 | 0.0899 | 4.1021 |
| | 89 ->101 | 6.2698 | 197.75 | 0.0387 | 9.2185 |
| | 89 -> 99 | 6.306 | 196.61 | 0.0558 | -16.1767 |
| | 88 -> 99 | 6.323 | 196.09 | 0.0747 | -12.4851 |
| | 89 ->101 | 6.3314 | 195.82 | 0.1566 | 45.7312 |
| | 87 -> 97 | 6.3943 | 193.9 | 0.0126 | 8.0745 |
| | 86 -> 98 | 6.4053 | 193.57 | 0.0202 | -5.7307 |

| | | | | | |
|---|---|---|---|---|---|
| | 86 -> 94 | 6.4164 | 193.23 | 0.0032 | 0.2214 |
| | 86 -> 97 | 6.4219 | 193.07 | 0.0028 | -1.9326 |
| | 88 ->101 | 6.4359 | 192.64 | 0.0363 | -11.3067 |
| | 86 -> 93 | 6.5418 | 189.53 | 0.0005 | 1.455 |

[a]Excitation energy. [b]Wavelength. [c]Oscillator strength. [d]Rotatory strength in velocity form ($10^{-40}$cgs.).

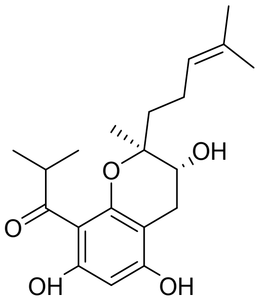


**2a**

Optimized geometries of predominant conformers for compound **2a** at the B3LYP/6-31G (d,p) level in methanol solution.

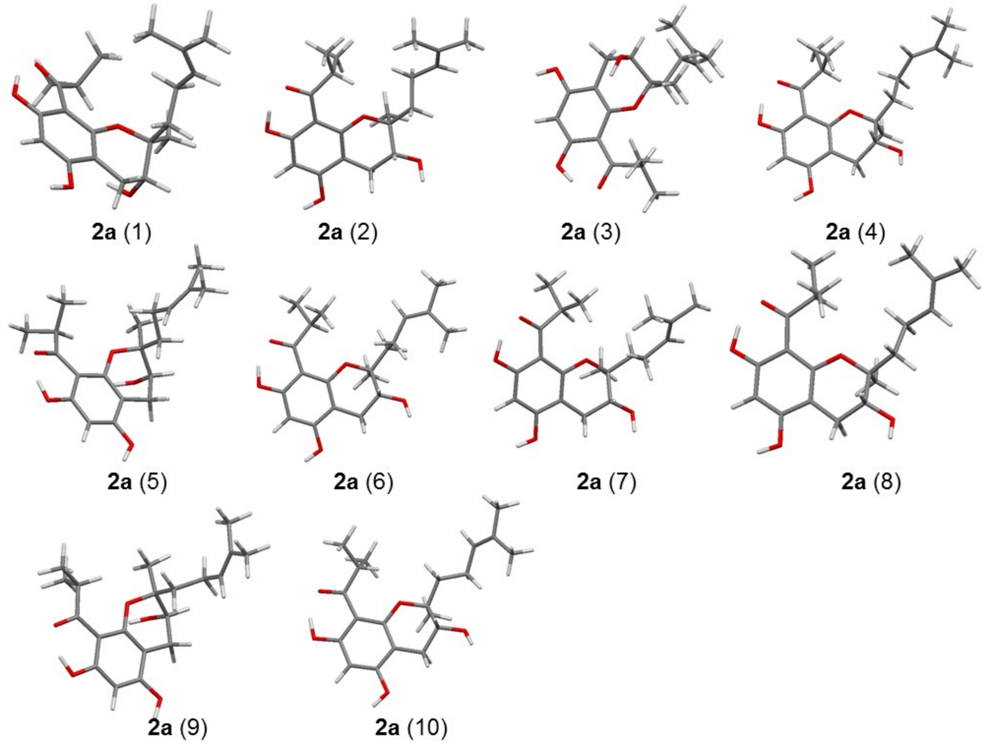


Important thermodynamic parameters (a.u.) and Boltzmann distributions of the optimized compound **2a** at B3LYP/6-31G(d,p) level in methanol solution.

| Conformations | E+ZPE | G | % |
|---|---|---|---|
| **2a** (1) | -1154.665845 | -1154.723759 | 18.10% |
| **2a** (2) | -1154.664538 | -1154.723920 | 1.20% |
| **2a** (3) | -1154.666434 | -1154.721195 | 21.47% |
| **2a** (4) | -1154.665194 | -1154.721751 | 2.16% |
| **2a** (5) | -1154.665818 | -1154.723254 | 10.61% |

| | | | |
|---|---|---|---|
| **2a** (6) | -1154.666594 | -1154.723604 | 15.36% |
| **2a** (7) | -1154.666080 | -1154.722340 | 4.03% |
| **2a** (8) | -1154.664929 | -1154.721973 | 2.73% |
| **2a** (9) | -1154.666307 | -1154.723578 | 14.95% |
| **2a** (10) | -1154.665295 | -1154.723139 | 9.39% |

E+ZPE, G: total energy with zero point energy (ZPE) and Gibbs free energy in methanol solution at B3LYP/6-31G (d,p) level. %: Boltzmann distributions, using the relative Gibbs free energies as weighting factors.

Optimized Z-matrixes of compound **2a** in methanol solution (Å) at B3LYP/6-31G (d,p) level.

| **2a** (1) | | | | **2a** (2) | | | |
|---|---|---|---|---|---|---|---|
| C | -2.093885 | 1.096032 | -2.510522 | C | 4.257628 | 0.165307 | -0.34937 |
| C | -1.65391 | -0.493694 | -0.674791 | C | 2.000141 | 1.154788 | -0.118603 |
| C | -0.840294 | 1.846515 | -0.544124 | C | 2.3121 | -1.307269 | -0.190852 |
| C | -0.922854 | 0.554789 | -0.022652 | C | 1.48255 | -0.189501 | -0.079197 |
| C | -2.187088 | -0.187822 | -1.970364 | C | 3.42553 | 1.281755 | -0.267139 |
| C | -1.445558 | 2.094038 | -1.793402 | C | 3.700415 | -1.104729 | -0.313101 |
| C | 3.961034 | -0.402223 | 0.056486 | C | -4.287022 | -0.745182 | 0.233821 |
| C | -1.958698 | -1.809993 | -0.101174 | C | 1.214698 | 2.395091 | -0.02744 |
| C | 4.212539 | -1.075631 | -1.077963 | C | -5.07221 | 0.129163 | -0.415849 |
| C | -0.126038 | 2.960556 | 0.18874 | C | 1.759451 | -2.710722 | -0.212404 |
| C | 0.175043 | 2.566455 | 1.637327 | C | 0.259137 | -2.705936 | -0.482257 |
| C | 0.731254 | 1.125291 | 1.69683 | C | -0.454985 | -1.640585 | 0.375709 |
| C | 5.549073 | -0.944902 | -1.771405 | C | -6.491487 | -0.237115 | -0.785202 |
| C | 3.242161 | -2.003924 | -1.76821 | C | -4.65935 | 1.518756 | -0.836853 |
| C | 0.906814 | 0.657017 | 3.139024 | C | -0.28174 | -1.888634 | 1.876183 |
| C | -0.64421 | -3.079743 | 1.615989 | C | -0.901283 | 3.744017 | -0.32612 |
| C | -3.153028 | -2.790155 | 1.873693 | C | -0.529443 | 2.324982 | 1.744269 |
| C | 2.703209 | -0.392891 | 0.886531 | C | -2.856935 | -0.582904 | 0.685052 |
| C | 2.027583 | 0.994235 | 0.868789 | C | -1.929397 | -1.562275 | -0.061583 |
| C | -1.844848 | -2.140544 | 1.387805 | C | -0.289823 | 2.447423 | 0.222177 |
| O | -2.409136 | -2.719652 | -0.836337 | O | 1.793724 | 3.505155 | -0.110985 |
| O | -0.297655 | 0.232314 | 1.145095 | O | 0.13382 | -0.337999 | 0.042463 |
| O | -2.818778 | -1.110884 | -2.711996 | O | 4.033249 | 2.475759 | -0.325947 |
| O | -1.335376 | 3.362119 | -2.265812 | O | 4.462602 | -2.22556 | -0.401033 |
| O | -0.98019 | 2.715567 | 2.465002 | O | -0.332655 | -3.969461 | -0.189381 |
| H | -2.526179 | 1.288894 | -3.487556 | H | 5.328182 | 0.315032 | -0.448213 |
| H | 4.755335 | 0.24134 | 0.441037 | H | -4.717642 | -1.721545 | 0.467361 |
| H | 0.800555 | 3.236922 | -0.331097 | H | 2.268992 | -3.29787 | -0.985661 |
| H | -0.746459 | 3.862927 | 0.199982 | H | 1.949819 | -3.229119 | 0.737079 |
| H | 0.917035 | 3.247575 | 2.063776 | H | 0.082738 | -2.43913 | -1.535577 |
| H | 6.04745 | -1.921011 | -1.855473 | H | -7.210301 | 0.462149 | -0.33502 |
| H | 5.426582 | -0.572006 | -2.798119 | H | -6.644178 | -0.178773 | -1.87219 |
| H | 6.221465 | -0.264095 | -1.239578 | H | -6.751067 | -1.249136 | -0.458438 |

| | | | | | | | |
|---|---|---|---|---|---|---|---|
| H | 3.049257 | -1.663253 | -2.794963 | H | -4.77499 | 1.642169 | -1.922574 |
| H | 3.667643 | -3.013484 | -1.85386 | H | -5.307307 | 2.27399 | -0.37043 |
| H | 2.280142 | -2.087312 | -1.257447 | H | -3.625086 | 1.759407 | -0.581266 |
| H | 1.130411 | -0.411753 | 3.177922 | H | -0.776095 | -1.107228 | 2.458116 |
| H | -0.002518 | 0.849378 | 3.711388 | H | 0.774783 | -1.896508 | 2.158124 |
| H | 1.734017 | 1.201112 | 3.607423 | H | -0.716395 | -2.855203 | 2.145966 |
| H | -0.765682 | -4.005354 | 1.042344 | H | -0.486293 | 4.62493 | 0.17139 |
| H | -0.569745 | -3.341067 | 2.677447 | H | -1.984821 | 3.735277 | -0.163141 |
| H | 0.297006 | -2.608358 | 1.316302 | H | -0.720859 | 3.848341 | -1.401603 |
| H | -3.355534 | -3.721045 | 1.336376 | H | -0.048388 | 3.155357 | 2.274338 |
| H | -4.009223 | -2.120795 | 1.730757 | H | -0.135639 | 1.385587 | 2.142144 |
| H | -3.076502 | -3.015776 | 2.94311 | H | -1.602904 | 2.362332 | 1.959283 |
| H | 1.989657 | -1.14528 | 0.53869 | H | -2.507404 | 0.443014 | 0.546904 |
| H | 2.962862 | -0.659358 | 1.919642 | H | -2.811598 | -0.786293 | 1.763578 |
| H | 1.819011 | 1.255524 | -0.173357 | H | -1.945788 | -1.318882 | -1.131642 |
| H | 2.73413 | 1.747951 | 1.242204 | H | -2.331932 | -2.576953 | 0.031103 |
| H | -1.677725 | -1.223864 | 1.950867 | H | -0.756874 | 1.594636 | -0.268852 |
| H | -2.757235 | -1.964706 | -2.189495 | H | 3.293503 | 3.154961 | -0.258982 |
| H | -1.788579 | 3.430043 | -3.122865 | H | 5.396028 | -1.974849 | -0.503997 |
| H | -1.657899 | 2.100713 | 2.137291 | H | 0.087734 | -4.627419 | -0.76617 |
| **2a** (3) | | | | **2a** (4) | | | |
| C | -2.064135 | 2.813502 | -0.895382 | C | 4.35329 | -0.401055 | -0.016062 |
| C | -2.250542 | 0.375195 | -0.527324 | C | 2.288008 | 0.962151 | 0.003715 |
| C | -0.385638 | 1.677933 | 0.474758 | C | 2.178271 | -1.517197 | -0.079595 |
| C | -1.054848 | 0.471397 | 0.262896 | C | 1.548031 | -0.272173 | -0.037258 |
| C | -2.73887 | 1.606533 | -1.084883 | C | 3.72123 | 0.842459 | -0.001073 |
| C | -0.906089 | 2.839508 | -0.129477 | C | 3.585559 | -1.556901 | -0.052672 |
| C | 4.020641 | -1.236553 | -0.767192 | C | -4.090438 | 0.435688 | -0.409974 |
| C | -2.987439 | -0.860248 | -0.835731 | C | 1.717823 | 2.316057 | 0.070942 |
| C | 4.947689 | -0.466378 | -1.359329 | C | -5.380766 | 0.258652 | -0.082529 |
| C | 0.850512 | 1.763981 | 1.342089 | C | 1.394642 | -2.802804 | -0.181822 |
| C | 1.01069 | 0.516595 | 2.214431 | C | -0.050532 | -2.540961 | -0.595482 |
| C | 0.76302 | -0.753581 | 1.375283 | C | -0.646296 | -1.359019 | 0.19635 |
| C | 5.651227 | -0.935573 | -2.612063 | C | -6.481314 | 0.710875 | -1.014583 |
| C | 5.386826 | 0.893783 | -0.872898 | C | -5.871778 | -0.379172 | 1.19489 |
| C | 0.760162 | -2.01406 | 2.236662 | C | -0.660728 | -1.610621 | 1.706193 |
| C | -3.023316 | -3.324194 | -1.386368 | C | -0.320934 | 2.64179 | -1.332653 |
| C | -3.158162 | -2.524168 | 1.01809 | C | -0.055574 | 3.949743 | 0.825538 |
| C | 3.212806 | -0.952725 | 0.4735 | C | -2.858151 | 0.054329 | 0.36987 |
| C | 1.701811 | -0.890867 | 0.157597 | C | -2.034548 | -1.016048 | -0.373912 |
| C | -2.56384 | -2.256674 | -0.38289 | C | 0.222766 | 2.618884 | 0.11363 |
| O | -4.04731 | -0.796797 | -1.503375 | O | 2.480385 | 3.312482 | 0.07437 |
| O | -0.615485 | -0.670211 | 0.865256 | O | 0.189892 | -0.184994 | -0.079688 |

| | | | | | | | |
|---|---|---|---|---|---|---|---|
| O | -3.855742 | 1.658946 | -1.825333 | O | 4.526125 | 1.915145 | 0.020768 |
| O | -0.216111 | 3.987709 | 0.09208 | O | 4.148803 | -2.792601 | -0.077467 |
| O | 0.143593 | 0.560337 | 3.349419 | O | -0.876741 | -3.680293 | -0.367912 |
| H | -2.465024 | 3.714386 | -1.349498 | H | 5.438287 | -0.438218 | -0.005482 |
| H | 3.798408 | -2.198924 | -1.232962 | H | -3.881003 | 0.906923 | -1.372879 |
| H | 1.745951 | 1.909135 | 0.722478 | H | 1.869767 | -3.470131 | -0.910735 |
| H | 0.792339 | 2.638332 | 1.998316 | H | 1.397228 | -3.345598 | 0.773087 |
| H | 2.019299 | 0.481943 | 2.634365 | H | -0.077419 | -2.258981 | -1.659308 |
| H | 5.491245 | -0.232093 | -3.441258 | H | -7.123784 | -0.132277 | -1.305565 |
| H | 6.738231 | -0.988422 | -2.457784 | H | -7.137767 | 1.443621 | -0.524251 |
| H | 5.304428 | -1.923506 | -2.931565 | H | -6.08379 | 1.167019 | -1.926911 |
| H | 6.462495 | 0.894472 | -0.648166 | H | -6.5023 | 0.321783 | 1.759565 |
| H | 5.235499 | 1.648791 | -1.656636 | H | -6.503914 | -1.249863 | 0.971701 |
| H | 4.857681 | 1.229141 | 0.022029 | H | -5.066234 | -0.711162 | 1.853978 |
| H | 0.523107 | -2.891496 | 1.627031 | H | -1.05285 | -0.739435 | 2.236763 |
| H | 0.025212 | -1.926258 | 3.038757 | H | 0.344373 | -1.8106 | 2.087601 |
| H | 1.743368 | -2.164716 | 2.692163 | H | -1.292283 | -2.474079 | 1.933678 |
| H | -4.113513 | -3.357586 | -1.466156 | H | 0.174954 | 3.426943 | -1.915554 |
| H | -2.670718 | -4.308402 | -1.058324 | H | -1.395739 | 2.854658 | -1.322567 |
| H | -2.617808 | -3.134949 | -2.386507 | H | -0.169086 | 1.683541 | -1.836613 |
| H | -4.253147 | -2.481428 | 0.98406 | H | 0.398971 | 4.79103 | 0.295139 |
| H | -2.803092 | -1.796528 | 1.753023 | H | 0.335195 | 3.946291 | 1.849035 |
| H | -2.867145 | -3.523433 | 1.360621 | H | -1.137355 | 4.116907 | 0.875669 |
| H | 3.397246 | -1.753404 | 1.203393 | H | -2.240374 | 0.950747 | 0.513418 |
| H | 3.537995 | -0.021734 | 0.947569 | H | -3.122035 | -0.305308 | 1.368453 |
| H | 1.408639 | -1.806807 | -0.371169 | H | -1.889059 | -0.703798 | -1.416279 |
| H | 1.520695 | -0.064848 | -0.538603 | H | -2.608106 | -1.94797 | -0.405125 |
| H | -1.477998 | -2.287947 | -0.300782 | H | -0.278156 | 1.815077 | 0.651714 |
| H | -4.199857 | 0.714468 | -1.85613 | H | 3.910423 | 2.710866 | 0.034447 |
| H | -0.670417 | 4.729189 | -0.342093 | H | 5.11689 | -2.707238 | -0.078053 |
| H | -0.769705 | 0.522313 | 3.018843 | H | -0.523913 | -4.406738 | -0.906579 |
| **2a** (5) | | | | **2a** (6) | | | |
| C | 2.771371 | -1.74893 | -1.747691 | C | 4.027156 | -1.058338 | -0.776186 |
| C | 2.005914 | 0.331636 | -0.662659 | C | 2.373991 | 0.695153 | -0.222587 |
| C | 1.108554 | -1.869958 | 0.047027 | C | 1.767871 | -1.711275 | -0.102621 |
| C | 1.119821 | -0.476582 | 0.125866 | C | 1.432698 | -0.361977 | 0.028141 |
| C | 2.791039 | -0.356833 | -1.645505 | C | 3.681031 | 0.288366 | -0.661657 |
| C | 1.954223 | -2.485122 | -0.898837 | C | 3.082225 | -2.03554 | -0.490847 |
| C | -3.549769 | 0.264346 | -1.177913 | C | -3.971826 | 0.770963 | -0.416383 |
| C | 2.224858 | 1.77368 | -0.497598 | C | 2.13455 | 2.132349 | -0.025373 |
| C | -4.880703 | 0.424055 | -1.260609 | C | -5.253135 | 0.471002 | -0.684404 |
| C | 0.22522 | -2.714158 | 0.938641 | C | 0.755971 | -2.808663 | 0.132317 |
| C | -0.340261 | -1.885533 | 2.094553 | C | -0.665007 | -2.250048 | 0.131492 |

| | | | | | | | |
|---|---|---|---|---|---|---|---|
| C | -0.862241 | -0.527042 | 1.571987 | C | -0.742694 | -0.965976 | 0.975247 |
| C | -5.622731 | 0.062768 | -2.526818 | C | -6.331838 | 1.523495 | -0.569357 |
| C | -5.760092 | 0.960466 | -0.156764 | C | -5.75318 | -0.886904 | -1.115623 |
| C | -1.305942 | 0.375082 | 2.720996 | C | -0.317661 | -1.182884 | 2.432109 |
| C | 0.604156 | 3.470674 | 0.382169 | C | -0.115121 | 3.05232 | -0.583551 |
| C | 2.989026 | 3.405335 | 1.246741 | C | 1.170042 | 3.97854 | 1.399183 |
| C | -2.63564 | 0.558274 | -0.016473 | C | -2.762624 | -0.126705 | -0.466844 |
| C | -1.967781 | -0.72742 | 0.513393 | C | -2.130636 | -0.304628 | 0.928505 |
| C | 1.801447 | 2.567907 | 0.738883 | C | 0.857856 | 2.723918 | 0.569978 |
| O | 2.854784 | 2.411308 | -1.373961 | O | 3.017746 | 2.959189 | -0.358037 |
| O | 0.273653 | 0.182546 | 0.966408 | O | 0.164516 | 0.004414 | 0.35861 |
| O | 3.596451 | 0.299567 | -2.494763 | O | 4.638723 | 1.177611 | -0.963547 |
| O | 1.904425 | -3.841099 | -0.946795 | O | 3.367108 | -3.35995 | -0.58828 |
| O | 0.627834 | -1.712606 | 3.130648 | O | -1.612031 | -3.172381 | 0.663126 |
| H | 3.394478 | -2.230905 | -2.494682 | H | 5.032844 | -1.315645 | -1.093622 |
| H | -3.041291 | -0.14191 | -2.055079 | H | -3.754147 | 1.795778 | -0.107198 |
| H | -0.590818 | -3.169383 | 0.362688 | H | 0.850809 | -3.575654 | -0.645618 |
| H | 0.80001 | -3.546141 | 1.358691 | H | 0.940258 | -3.318391 | 1.087806 |
| H | -1.168552 | -2.419566 | 2.569122 | H | -0.93477 | -1.980733 | -0.898927 |
| H | -6.387831 | -0.701814 | -2.33125 | H | -6.852251 | 1.662513 | -1.52758 |
| H | -6.154778 | 0.93379 | -2.934881 | H | -7.099849 | 1.223957 | 0.15775 |
| H | -4.949753 | -0.31853 | -3.301465 | H | -5.927502 | 2.492107 | -0.258221 |
| H | -6.277082 | 1.873527 | -0.483436 | H | -6.520342 | -1.252848 | -0.419219 |
| H | -6.546021 | 0.234892 | 0.094496 | H | -6.235982 | -0.826715 | -2.101051 |
| H | -5.214081 | 1.193926 | 0.760108 | H | -4.965838 | -1.641922 | -1.173325 |
| H | -1.506678 | 1.389212 | 2.366818 | H | -0.465973 | -0.263046 | 3.006649 |
| H | -0.532133 | 0.415973 | 3.489917 | H | 0.735842 | -1.46623 | 2.508648 |
| H | -2.222238 | -0.022264 | 3.170835 | H | -0.921115 | -1.976832 | 2.880811 |
| H | 0.873113 | 4.163177 | -0.423211 | H | 0.32709 | 3.794421 | -1.258508 |
| H | 0.305053 | 4.059659 | 1.256269 | H | -1.045108 | 3.468631 | -0.18061 |
| H | -0.259832 | 2.8825 | 0.057624 | H | -0.364971 | 2.160344 | -1.165175 |
| H | 3.325353 | 4.114467 | 0.485186 | H | 1.620373 | 4.762569 | 0.784293 |
| H | 3.839044 | 2.769605 | 1.520286 | H | 1.860377 | 3.754321 | 2.220249 |
| H | 2.68813 | 3.96809 | 2.137398 | H | 0.243195 | 4.369125 | 1.833969 |
| H | -1.847349 | 1.247771 | -0.349603 | H | -3.01657 | -1.106242 | -0.881927 |
| H | -3.172884 | 1.063779 | 0.790402 | H | -2.015672 | 0.318748 | -1.138943 |
| H | -1.550123 | -1.275128 | -0.338528 | H | -2.803912 | -0.901203 | 1.553123 |
| H | -2.736744 | -1.377368 | 0.951196 | H | -2.037833 | 0.674578 | 1.415521 |
| H | 1.494619 | 1.880082 | 1.524962 | H | 0.3859 | 1.981185 | 1.211175 |
| H | 3.454831 | 1.273682 | -2.301394 | H | 4.217697 | 2.080291 | -0.824028 |
| H | 2.522299 | -4.165545 | -1.623326 | H | 4.287409 | -3.473871 | -0.879526 |
| H | 1.381277 | -1.229185 | 2.752212 | H | -1.586476 | -3.969296 | 0.109277 |
| **2a** (7) | | | | **2a** (8) | | | |

| | | | | | | | |
|---|---|---|---|---|---|---|---|
| C | 4.015677 | -0.229195 | -0.95459 | C | 4.285672 | 0.25253 | -0.114948 |
| C | 2.024166 | 1.015393 | -0.189965 | C | 1.998849 | 1.188404 | 0.025122 |
| C | 2.036137 | -1.466711 | -0.218429 | C | 2.365477 | -1.261047 | -0.143006 |
| C | 1.384779 | -0.253841 | 0.014547 | C | 1.509728 | -0.16463 | -0.023085 |
| C | 3.352656 | 0.978432 | -0.734334 | C | 3.427406 | 1.348063 | -0.017473 |
| C | 3.364516 | -1.42706 | -0.685542 | C | 3.753658 | -1.027657 | -0.179786 |
| C | -4.207405 | -0.669961 | -0.271512 | C | -4.259079 | -0.885475 | 0.209614 |
| C | 1.46738 | 2.326892 | 0.167694 | C | 1.179248 | 2.407062 | 0.113008 |
| C | -4.880828 | 0.318464 | -0.8821 | C | -5.12288 | 0.088531 | -0.118635 |
| C | 1.338663 | -2.792076 | -0.018973 | C | 1.842627 | -2.672476 | -0.250693 |
| C | -0.175726 | -2.60311 | 0.029391 | C | 0.344246 | -2.695096 | -0.538163 |
| C | -0.549064 | -1.428656 | 0.950965 | C | -0.402141 | -1.665287 | 0.335177 |
| C | -6.349584 | 0.538715 | -0.600972 | C | -6.500674 | -0.248265 | -0.641225 |
| C | -4.289545 | 1.280127 | -1.884519 | C | -4.847397 | 1.568094 | -0.003175 |
| C | -0.050348 | -1.619388 | 2.38809 | C | -0.232194 | -1.932707 | 1.832591 |
| C | -0.982899 | 2.802576 | 0.074102 | C | -0.766157 | 2.293857 | -1.445015 |
| C | 0.40885 | 3.684642 | 2.005208 | C | -0.922502 | 3.698368 | 0.663863 |
| C | -2.757172 | -1.055915 | -0.413484 | C | -2.852586 | -0.772741 | 0.742544 |
| C | -2.058144 | -1.128933 | 0.958064 | C | -1.875247 | -1.606071 | -0.109574 |
| C | 0.210113 | 2.527418 | 1.013164 | C | -0.344703 | 2.422873 | 0.0369 |
| O | 2.054861 | 3.372002 | -0.200622 | O | 1.738576 | 3.526062 | 0.205587 |
| O | 0.077596 | -0.22798 | 0.392358 | O | 0.160361 | -0.346519 | 0.022366 |
| O | 4.03065 | 2.098168 | -1.029149 | O | 4.012057 | 2.554195 | 0.035949 |
| O | 3.963991 | -2.629606 | -0.884281 | O | 4.541353 | -2.129223 | -0.285249 |
| O | -0.853815 | -3.759565 | 0.512218 | O | -0.218729 | -3.97897 | -0.277702 |
| H | 5.029586 | -0.20831 | -1.341851 | H | 5.357066 | 0.426204 | -0.139243 |
| H | -4.768154 | -1.296092 | 0.426402 | H | -4.601139 | -1.915242 | 0.082263 |
| H | 1.598409 | -3.477101 | -0.834571 | H | 2.377147 | -3.20653 | -1.045563 |
| H | 1.671317 | -3.278775 | 0.907976 | H | 2.033305 | -3.235198 | 0.673173 |
| H | -0.526951 | -2.351637 | -0.980751 | H | 0.170151 | -2.409964 | -1.587025 |
| H | -6.529614 | 1.550983 | -0.212109 | H | -7.283112 | 0.173151 | 0.005655 |
| H | -6.945614 | 0.451595 | -1.520489 | H | -6.660594 | 0.183598 | -1.639402 |
| H | -6.739878 | -0.179994 | 0.126772 | H | -6.660342 | -1.329209 | -0.708444 |
| H | -4.807203 | 1.195985 | -2.850362 | H | -4.954086 | 2.060423 | -0.979907 |
| H | -4.426007 | 2.318359 | -1.55156 | H | -5.579645 | 2.045036 | 0.663183 |
| H | -3.223699 | 1.120965 | -2.062301 | H | -3.850766 | 1.794594 | 0.382197 |
| H | -0.416665 | -0.806657 | 3.023303 | H | -0.720949 | -1.151705 | 2.42013 |
| H | 1.042205 | -1.625515 | 2.439171 | H | 0.823106 | -1.953611 | 2.117519 |
| H | -0.419144 | -2.568181 | 2.787715 | H | -0.676603 | -2.897917 | 2.091641 |
| H | -0.80654 | 3.711237 | -0.512935 | H | -0.376743 | 3.136142 | -2.029112 |
| H | -1.896734 | 2.945845 | 0.661388 | H | -1.858793 | 2.304511 | -1.523376 |
| H | -1.149291 | 1.970287 | -0.615838 | H | -0.403075 | 1.36363 | -1.890057 |
| H | 0.600055 | 4.627055 | 1.484692 | H | -0.595409 | 4.593355 | 0.127017 |

| | | | | | | | |
|---|---|---|---|---|---|---|---|
| H | 1.250837 | 3.492626 | 2.679989 | H | -0.617368 | 3.804517 | 1.710499 |
| H | -0.493366 | 3.802422 | 2.61567 | H | -2.017074 | 3.658612 | 0.631205 |
| H | -2.702752 | -2.046165 | -0.887875 | H | -2.524044 | 0.269564 | 0.789318 |
| H | -2.218686 | -0.363169 | -1.067227 | H | -2.839894 | -1.150426 | 1.774818 |
| H | -2.541688 | -1.901807 | 1.566081 | H | -1.892303 | -1.23896 | -1.143436 |
| H | -2.197948 | -0.179453 | 1.489299 | H | -2.230732 | -2.6419 | -0.141232 |
| H | 0.003744 | 1.614482 | 1.569678 | H | -0.726596 | 1.554288 | 0.572318 |
| H | 3.402865 | 2.85389 | -0.819894 | H | 3.256861 | 3.212953 | 0.12391 |
| H | 4.860584 | -2.492417 | -1.233505 | H | 5.473924 | -1.858393 | -0.323831 |
| H | -0.649266 | -4.491504 | -0.0919 | H | 0.220654 | -4.613846 | -0.866075 |
| **2a** (9) | | | | **2a** (10) | | | |
| C | 2.88147 | -2.116748 | -1.338818 | C | 4.14518 | -1.357368 | -0.337597 |
| C | 2.317204 | 0.149053 | -0.523819 | C | 2.611227 | 0.572466 | -0.131972 |
| C | 0.939736 | -1.818567 | 0.118622 | C | 1.740828 | -1.75305 | -0.132301 |
| C | 1.207231 | -0.449165 | 0.164016 | C | 1.534394 | -0.376687 | -0.040188 |
| C | 3.15553 | -0.749951 | -1.268537 | C | 3.931266 | 0.019794 | -0.265844 |
| C | 1.788869 | -2.633931 | -0.655335 | C | 3.060251 | -2.222456 | -0.277551 |
| C | -4.082793 | -0.377086 | -0.93871 | C | -4.510862 | 0.666712 | 0.373275 |
| C | 2.647286 | 1.582804 | -0.553247 | C | 2.475943 | 2.036132 | -0.133044 |
| C | -5.123217 | 0.41432 | -1.24595 | C | -5.611537 | 0.490576 | -0.375733 |
| C | -0.208027 | -2.434474 | 0.88837 | C | 0.587942 | -2.724425 | -0.089576 |
| C | -0.745237 | -1.474645 | 1.951917 | C | -0.748085 | -2.017355 | -0.310648 |
| C | -0.906193 | -0.061015 | 1.355749 | C | -0.837469 | -0.735667 | 0.540842 |
| C | -5.780634 | 0.328117 | -2.604149 | C | -6.582726 | 1.626999 | -0.599552 |
| C | -5.738756 | 1.436789 | -0.321289 | C | -6.001129 | -0.802404 | -1.051119 |
| C | -1.307402 | 0.961207 | 2.416316 | C | -0.717908 | -1.005664 | 2.042809 |
| C | 1.961454 | 4.013622 | -0.589855 | C | 1.24146 | 4.107197 | -0.880789 |
| C | 2.318139 | 2.793835 | 1.606617 | C | 0.73132 | 3.022826 | 1.355214 |
| C | -3.293057 | -0.43622 | 0.34411 | C | -3.433591 | -0.331812 | 0.711327 |
| C | -1.828625 | 0.002568 | 0.119997 | C | -2.058759 | 0.138879 | 0.19178 |
| C | 1.833212 | 2.671473 | 0.144291 | C | 1.141212 | 2.780167 | -0.114084 |
| O | 3.669345 | 1.968732 | -1.169143 | O | 3.504931 | 2.753966 | -0.147706 |
| O | 0.434662 | 0.373667 | 0.930171 | O | 0.283438 | 0.123522 | 0.133152 |
| O | 4.233089 | -0.323598 | -1.943397 | O | 5.023276 | 0.796114 | -0.333149 |
| O | 1.482694 | -3.956298 | -0.684726 | O | 3.209936 | -3.570403 | -0.349874 |
| O | 0.098107 | -1.449961 | 3.105419 | O | -1.840249 | -2.866923 | 0.032536 |
| H | 3.53625 | -2.752814 | -1.926645 | H | 5.161361 | -1.725587 | -0.43942 |
| H | -3.735602 | -1.068288 | -1.709479 | H | -4.352522 | 1.660389 | 0.799065 |
| H | -1.016238 | -2.729152 | 0.204997 | H | 0.723392 | -3.496331 | -0.856947 |
| H | 0.119451 | -3.353513 | 1.384887 | H | 0.549669 | -3.254842 | 0.871711 |
| H | -1.711785 | -1.827974 | 2.320195 | H | -0.825082 | -1.706552 | -1.363687 |
| H | -6.846799 | 0.077585 | -2.511417 | H | -6.693477 | 1.846671 | -1.670918 |
| H | -5.734997 | 1.294336 | -3.126063 | H | -7.58618 | 1.367714 | -0.233003 |

| | | | | | | | |
|---|---|---|---|---|---|---|---|
| H | -5.307479 | -0.426056 | -3.24108 | H | -6.263209 | 2.544574 | -0.095026 |
| H | -5.71143 | 2.434592 | -0.780499 | H | -6.974985 | -1.1539 | -0.68215 |
| H | -6.798927 | 1.208515 | -0.143874 | H | -6.118929 | -0.65112 | -2.133135 |
| H | -5.242665 | 1.501715 | 0.649796 | H | -5.276088 | -1.605455 | -0.901 |
| H | -1.347897 | 1.965221 | 1.982678 | H | -0.871954 | -0.080974 | 2.60798 |
| H | -0.594298 | 0.956776 | 3.242706 | H | 0.270503 | -1.399413 | 2.296548 |
| H | -2.295337 | 0.718642 | 2.819184 | H | -1.466401 | -1.737385 | 2.357743 |
| H | 2.994059 | 4.373782 | -0.591428 | H | 1.976087 | 4.777706 | -0.426467 |
| H | 1.335626 | 4.762475 | -0.091667 | H | 0.266275 | 4.6067 | -0.874418 |
| H | 1.630776 | 3.934038 | -1.631349 | H | 1.532085 | 3.945459 | -1.924895 |
| H | 3.377083 | 3.075976 | 1.637262 | H | 1.479306 | 3.641495 | 1.864796 |
| H | 2.190838 | 1.856209 | 2.154505 | H | 0.62527 | 2.083185 | 1.904441 |
| H | 1.744128 | 3.570549 | 2.124003 | H | -0.228664 | 3.55 | 1.391887 |
| H | -3.753991 | 0.181839 | 1.119831 | H | -3.384036 | -0.43667 | 1.804744 |
| H | -3.311769 | -1.469794 | 0.718421 | H | -3.655376 | -1.323093 | 0.31422 |
| H | -1.820887 | 1.039741 | -0.236364 | H | -1.853023 | 1.138618 | 0.595302 |
| H | -1.395241 | -0.600713 | -0.685837 | H | -2.101189 | 0.24641 | -0.899527 |
| H | 0.785753 | 2.372356 | 0.160519 | H | 0.379669 | 2.156874 | -0.580867 |
| H | 4.27117 | 0.668923 | -1.785727 | H | 4.685083 | 1.739847 | -0.253096 |
| H | 2.138039 | -4.429536 | -1.224332 | H | 4.150842 | -3.786932 | -0.461012 |
| H | 0.941843 | -1.04768 | 2.838417 | H | -1.792818 | -3.64676 | -0.544038 |

Key transitions, oscillator strengths, and rotatory strengths in the ECD of conformers **2a** (3) at B3LYP /6-311++G(d,p) level.

| Species | Excited State | $\Delta E(eV)^a$ | $\lambda(nm)^b$ | $f^c$ | $R_{vel}^d$ |
|---|---|---|---|---|---|
| **2a** (3) | 92 -> 97 | 3.7469 | 330.9 | 0.0712 | 11.6899 |
| | 91 ->106 | 4.1588 | 298.12 | 0.0014 | 3.0508 |
| | 94 -> 97 | 4.316 | 287.27 | 0.0513 | 16.2671 |
| | 93 -> 95 | 4.4985 | 275.61 | 0.3459 | 8.166 |
| | 94 -> 99 | 5.2052 | 238.19 | 0.0058 | 0.0829 |
| | 93 -> 97 | 5.2535 | 236 | 0.0032 | 4.7223 |
| | 93 -> 97 | 5.4987 | 225.48 | 0.1074 | -9.7329 |
| | 93 ->100 | 5.5233 | 224.47 | 0.0101 | 5.7499 |
| | 94 -> 99 | 5.5875 | 221.9 | 0.0272 | -13.6235 |
| | 92 -> 97 | 5.6153 | 220.8 | 0.0354 | -19.1975 |
| | 93 -> 96 | 5.6392 | 219.86 | 0.0003 | 1.0407 |
| | 89 -> 95 | 5.7168 | 216.88 | 0.0095 | 1.6304 |
| | 94 -> 99 | 5.7663 | 215.01 | 0.0193 | 1.2329 |
| | 93 -> 97 | 5.8327 | 212.57 | 0.1117 | -110.0444 |
| | 93 -> 96 | 5.9076 | 209.87 | 0.0131 | -14.4686 |
| | 93 -> 99 | 5.9297 | 209.09 | 0.0473 | -1.0213 |
| | 93 -> 98 | 5.9471 | 208.48 | 0.0451 | 39.8988 |
| | 94 -> 99 | 5.9861 | 207.12 | 0.0117 | 8.8114 |

| | | | | | |
|---|---|---|---|---|---|
| | 94 ->101 | 6.0176 | 206.04 | 0.0114 | 10.1219 |
| | 93 ->100 | 6.034 | 205.48 | 0.0002 | 0.0822 |
| | 92 -> 97 | 6.0734 | 204.14 | 0.0077 | 3.5274 |
| | 92 -> 98 | 6.0761 | 204.05 | 0.0059 | 8.5484 |
| | 93 ->100 | 6.1107 | 202.9 | 0.0032 | -0.4824 |
| | 93 ->101 | 6.1488 | 201.64 | 0.0035 | -6.4037 |
| | 94 ->103 | 6.2123 | 199.58 | 0.0137 | 9.378 |
| | 94 ->105 | 6.2245 | 199.19 | 0.0359 | 40.7268 |
| | 94 ->102 | 6.2422 | 198.62 | 0.0098 | 10.5549 |
| | 91 -> 99 | 6.2706 | 197.72 | 0.0007 | -2.1583 |
| | 93 -> 96 | 6.3124 | 196.41 | 0.2346 | 204.466 |
| | 92 -> 98 | 6.3457 | 195.38 | 0.0583 | 17.0626 |
| | 93 ->101 | 6.3749 | 194.49 | 0.1572 | -139.9957 |
| | 92 ->100 | 6.4185 | 193.17 | 0.0082 | -0.957 |
| | 93 ->103 | 6.4277 | 192.89 | 0.0554 | -13.8562 |
| | 93 ->103 | 6.4408 | 192.5 | 0.0133 | 21.335 |
| | 94 ->110 | 6.4684 | 191.68 | 0.0971 | 70.286 |
| | 93 ->103 | 6.4772 | 191.42 | 0.0278 | -57.4108 |
| | 84 -> 95 | 6.5113 | 190.42 | 0.0234 | -16.8602 |
| | 93 ->105 | 6.5568 | 189.09 | 0.0147 | -14.5178 |
| | 93 ->104 | 6.6013 | 187.82 | 0.012 | -15.2854 |
| | 94 ->111 | 6.6096 | 187.58 | 0.0023 | -3.2513 |

[a]Excitation energy. [b]Wavelength. [c]Oscillator strength. [d]Rotatory strength in velocity form ($10^{-40}$cgs.).

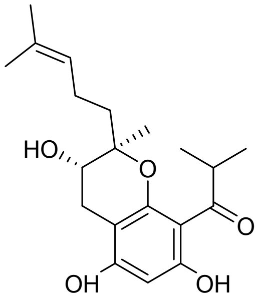


**2b**

Optimized geometries of predominant conformers for compound **2b** at the B3LYP/6-31G (d,p) level in methanol solution.

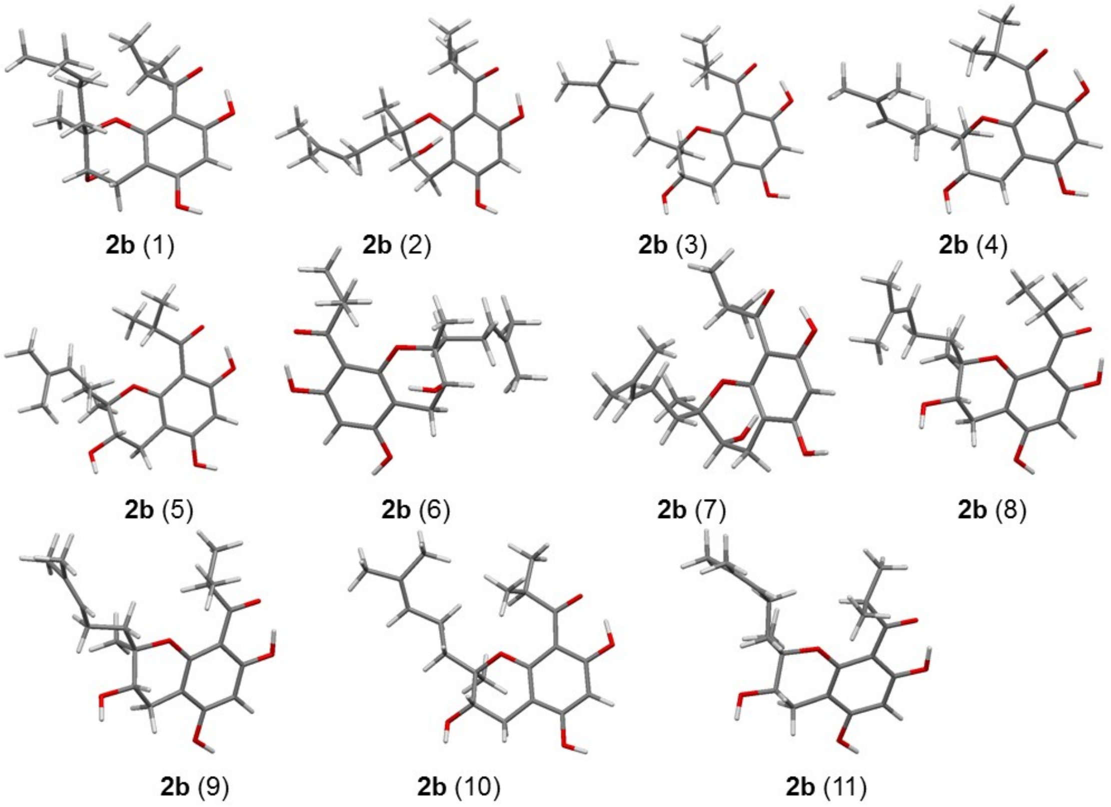


Important thermodynamic parameters (a.u.) and Boltzmann distributions of the optimized compound **2b** at B3LYP/6-31G(d,p) level in methanol solution.

| Conformations | E+ZPE | G | % |
|---|---|---|---|
| **2b** (1) | -1154.665820 | -1154.723263 | 9.26% |
| **2b** (2) | -1154.666309 | -1154.723587 | 13.05% |
| **2b** (3) | -1154.664541 | -1154.721202 | 1.04% |
| **2b** (4) | -1154.666078 | -1154.722336 | 3.47% |
| **2b** (5) | -1154.666733 | -1154.724088 | 22.19% |
| **2b** (6) | -1154.666434 | -1154.723920 | 18.57% |
| **2b** (7) | -1154.666457 | -1154.723650 | 13.95% |
| **2b** (8) | -1154.665296 | -1154.723141 | 8.14% |
| **2b** (9) | -1154.665294 | -1154.722958 | 6.71% |
| **2b** (10) | -1154.664872 | -1154.721693 | 1.76% |
| **2b** (11) | -1154.665193 | -1154.721746 | 1.86% |

E+ZPE, G: total energy with zero point energy (ZPE) and Gibbs free energy in methanol solution at B3LYP/6-31G (d,p) level. %: Boltzmann distributions, using the relative Gibbs free energies as weighting factors.

Optimized Z-matrixes of compound **2b** in methanol solution (Å) at B3LYP/6-31G (d,p) level.

| **2b** (1) | | | | **2b** (2) | | | |
|---|---|---|---|---|---|---|---|
| C | -2.773941 | -1.747107 | -1.747337 | C | -2.881704 | -2.116066 | -1.33936 |
| C | -2.006461 | 0.332773 | -0.662341 | C | -2.317028 | 0.149453 | -0.523838 |
| C | -1.110105 | -1.869586 | 0.046351 | C | -0.94006 | -1.818641 | 0.118343 |
| C | -1.120311 | -0.476214 | 0.125482 | C | -1.207245 | -0.449176 | 0.163993 |

| | | | | | | | |
|---|---|---|---|---|---|---|---|
| C | -2.792653 | -0.355012 | -1.644804 | C | -3.155443 | -0.749224 | -1.268869 |
| C | -1.956773 | -2.483989 | -0.899131 | C | -1.789308 | -2.633651 | -0.655842 |
| C | 3.551354 | 0.261698 | -1.176998 | C | 4.082786 | -0.37728 | -0.938873 |
| C | -2.224353 | 1.774903 | -0.496894 | C | -2.6469 | 1.583277 | -0.55288 |
| C | 4.882106 | 0.422692 | -1.260224 | C | 5.122936 | 0.414538 | -1.245981 |
| C | -0.226847 | -2.714562 | 0.937294 | C | 0.207554 | -2.434957 | 0.887963 |
| C | 0.338924 | -1.886603 | 2.093514 | C | 0.745131 | -1.47547 | 1.95165 |
| C | 0.861967 | -0.528374 | 1.571459 | C | 0.906344 | -0.061766 | 1.355806 |
| C | 5.624107 | 0.060969 | -2.526324 | C | 5.780156 | 0.329058 | -2.604321 |
| C | 5.761319 | 0.96106 | -0.15719 | C | 5.738288 | 1.436901 | -0.321085 |
| C | 1.305835 | 0.373211 | 2.720799 | C | 1.307686 | 0.960218 | 2.416525 |
| C | -2.985052 | 3.407656 | 1.248021 | C | -2.318015 | 2.793649 | 1.607232 |
| C | -0.60093 | 3.470107 | 0.381249 | C | -1.960546 | 4.013981 | -0.588775 |
| C | 2.637107 | 0.555961 | -0.015738 | C | 3.293125 | -0.436933 | 0.343965 |
| C | 1.967746 | -0.729313 | 0.513203 | C | 1.828683 | 0.001932 | 0.119987 |
| C | -1.798947 | 2.568797 | 0.739161 | C | -1.832728 | 2.67161 | 0.144973 |
| O | -2.854836 | 2.413013 | -1.372522 | O | -3.668886 | 1.969487 | -1.168699 |
| O | -0.273108 | 0.182257 | 0.96536 | O | -0.434567 | 0.37326 | 0.930422 |
| O | -3.598202 | 0.302068 | -2.493408 | O | -4.232809 | -0.322517 | -1.943901 |
| O | -1.907901 | -3.839998 | -0.947342 | O | -1.483467 | -3.956101 | -0.685433 |
| O | -0.629313 | -1.713393 | 3.129458 | O | -0.098004 | -1.450868 | 3.105289 |
| H | -3.397836 | -2.228479 | -2.494055 | H | -3.536545 | -2.751879 | -1.927394 |
| H | 3.043065 | -0.14587 | -2.05367 | H | 3.735709 | -1.068301 | -1.709854 |
| H | -0.801788 | -3.546589 | 1.357036 | H | -0.120118 | -3.353993 | 1.384356 |
| H | 0.588984 | -3.169722 | 0.361003 | H | 1.015659 | -2.729764 | 0.204521 |
| H | 1.166806 | -2.421243 | 2.568112 | H | 1.711667 | -1.829112 | 2.319656 |
| H | 6.155237 | 0.932121 | -2.935311 | H | 5.734255 | 1.295521 | -3.12577 |
| H | 6.389981 | -0.702725 | -2.330306 | H | 6.846386 | 0.078688 | -2.511908 |
| H | 4.95127 | -0.321633 | -3.300449 | H | 5.307032 | -0.424883 | -3.241548 |
| H | 6.548484 | 0.236819 | 0.09404 | H | 6.798893 | 1.209655 | -0.144978 |
| H | 6.276747 | 1.874714 | -0.484677 | H | 5.709379 | 2.435075 | -0.77941 |
| H | 5.215498 | 1.194265 | 0.759855 | H | 5.243153 | 1.500458 | 0.650579 |
| H | 1.507481 | 1.387255 | 2.366899 | H | 1.348456 | 1.964282 | 1.983036 |
| H | 2.221647 | -0.024894 | 3.170955 | H | 2.295503 | 0.717366 | 2.819504 |
| H | 0.531738 | 0.414554 | 3.489407 | H | 0.594469 | 0.955831 | 3.242819 |
| H | -3.321141 | 4.117225 | 0.486773 | H | -3.377011 | 3.075612 | 1.637696 |
| H | -2.682733 | 3.970028 | 2.138443 | H | -1.744275 | 3.570385 | 2.124903 |
| H | -3.835643 | 2.772999 | 1.522278 | H | -2.19066 | 1.855979 | 2.155026 |
| H | -0.869693 | 4.162665 | -0.424154 | H | -2.993033 | 4.374506 | -0.590269 |
| H | 0.262153 | 2.880848 | 0.056276 | H | -1.629894 | 3.934691 | -1.630302 |
| H | -0.300504 | 4.059089 | 1.254902 | H | -1.334477 | 4.76245 | -0.090319 |
| H | 3.174578 | 1.06045 | 0.791615 | H | 3.31182 | -1.47063 | 0.717939 |
| H | 1.849655 | 1.246495 | -0.348732 | H | 3.754065 | 0.18087 | 1.119884 |

| | | | | | | | |
|---|---|---|---|---|---|---|---|
| H | 2.735889 | -1.380233 | 0.951014 | H | 1.395254 | -0.60124 | -0.68589 |
| H | 1.549862 | -1.276219 | -0.339123 | H | 1.82098 | 1.039144 | -0.23625 |
| H | -1.492181 | 1.880644 | 1.524979 | H | -0.785352 | 2.372243 | 0.161443 |
| H | -3.456014 | 1.276046 | -2.299719 | H | -4.270996 | 0.669916 | -1.785862 |
| H | -2.526208 | -4.163914 | -1.623738 | H | -2.13894 | -4.429097 | -1.225107 |
| H | -1.383016 | -1.230663 | 2.750647 | H | -0.941784 | -1.048573 | 2.838417 |
| **2b** (3) | | | | **2b** (4) | | | |
| C | -4.257667 | 0.16543 | -0.349073 | C | -4.015691 | -0.228885 | -0.954441 |
| C | -2.000118 | 1.154829 | -0.118642 | C | -2.024015 | 1.015549 | -0.189971 |
| C | -2.312164 | -1.307219 | -0.190788 | C | -2.036161 | -1.466581 | -0.218677 |
| C | -1.482583 | -0.189465 | -0.079276 | C | -1.384744 | -0.25377 | 0.01449 |
| C | -3.425516 | 1.281852 | -0.267068 | C | -3.352597 | 0.978671 | -0.734045 |
| C | -3.700494 | -1.104624 | -0.312794 | C | -3.364552 | -1.426829 | -0.685698 |
| C | 4.287051 | -0.745496 | 0.233603 | C | 4.207226 | -0.669911 | -0.271194 |
| C | -1.214602 | 2.39509 | -0.027649 | C | -1.46709 | 2.327025 | 0.167798 |
| C | 5.072216 | 0.129144 | -0.415705 | C | 4.880631 | 0.318305 | -0.882113 |
| C | -1.759565 | -2.710688 | -0.212507 | C | -1.338771 | -2.792012 | -0.019271 |
| C | -0.259244 | -2.705991 | -0.482377 | C | 0.175613 | -2.603167 | 0.029279 |
| C | 0.454928 | -1.640569 | 0.37549 | C | 0.548892 | -1.428873 | 0.95107 |
| C | 6.491402 | -0.237055 | -0.785457 | C | 6.349347 | 0.538816 | -0.600949 |
| C | 4.659355 | 1.519045 | -0.83575 | C | 4.289331 | 1.279497 | -1.884961 |
| C | 0.28171 | -1.888533 | 1.875973 | C | 0.049978 | -1.619854 | 2.388072 |
| C | 0.529463 | 2.324843 | 1.744213 | C | -0.408035 | 3.684785 | 2.005026 |
| C | 0.901461 | 3.743999 | -0.326096 | C | 0.983243 | 2.802448 | 0.07372 |
| C | 2.857045 | -0.583422 | 0.685163 | C | 2.757029 | -1.055954 | -0.413149 |
| C | 1.92934 | -1.562333 | -0.061868 | C | 2.057981 | -1.129228 | 0.958373 |
| C | 0.289942 | 2.447391 | 0.222127 | C | -0.209655 | 2.527531 | 1.012979 |
| O | -1.793539 | 3.505169 | -0.111479 | O | -2.054555 | 3.372179 | -0.200409 |
| O | -0.133816 | -0.338019 | 0.042126 | O | -0.07766 | -0.22806 | 0.392601 |
| O | -4.033179 | 2.475886 | -0.325937 | O | -4.030691 | 2.098452 | -1.028599 |
| O | -4.462743 | -2.225432 | -0.400527 | O | -3.96411 | -2.629324 | -0.884494 |
| O | 0.33244 | -3.969534 | -0.189337 | O | 0.853587 | -3.759704 | 0.512061 |
| H | -5.32823 | 0.315185 | -0.447783 | H | -5.029688 | -0.207843 | -1.341455 |
| H | 4.717629 | -1.722024 | 0.466539 | H | 4.767971 | -1.295765 | 0.42697 |
| H | -1.949916 | -3.229178 | 0.736924 | H | -1.671537 | -3.278801 | 0.907585 |
| H | -2.269179 | -3.29774 | -0.985787 | H | -1.59847 | -3.47693 | -0.834971 |
| H | -0.082805 | -2.439309 | -1.535711 | H | 0.526967 | -2.351583 | -0.98078 |
| H | 6.644009 | -0.177942 | -1.872415 | H | 6.945377 | 0.4523 | -1.520523 |
| H | 7.210358 | 0.461783 | -0.334834 | H | 6.52914 | 1.550952 | -0.211618 |
| H | 6.750875 | -1.249348 | -0.459452 | H | 6.73988 | -0.180113 | 0.12645 |
| H | 5.30719 | 2.273972 | -0.368657 | H | 4.426474 | 2.317914 | -1.552866 |
| H | 4.775104 | 1.643287 | -1.921353 | H | 4.806498 | 1.194327 | -2.850979 |
| H | 3.625054 | 1.759427 | -0.580071 | H | 3.223316 | 1.120715 | -2.06211 |

| | | | | | | | |
|---|---|---|---|---|---|---|---|
| H | 0.775872 | -1.106955 | 2.457839 | H | 0.416288 | -0.807319 | 3.023535 |
| H | 0.71662 | -2.854961 | 2.145865 | H | 0.418634 | -2.568783 | 2.7875 |
| H | -0.774806 | -1.896662 | 2.157924 | H | -1.042581 | -1.62589 | 2.439 |
| H | 0.048099 | 3.155011 | 2.274336 | H | -0.598293 | 4.627472 | 1.484652 |
| H | 1.60289 | 2.362528 | 1.959346 | H | 0.494023 | 3.801822 | 2.615869 |
| H | 0.135909 | 1.385289 | 2.141958 | H | -1.250443 | 3.493346 | 2.679444 |
| H | 0.486461 | 4.62492 | 0.17138 | H | 0.806701 | 3.710789 | -0.513764 |
| H | 0.721091 | 3.848321 | -1.401591 | H | 1.149776 | 1.969848 | -0.615802 |
| H | 1.984987 | 3.735221 | -0.163084 | H | 1.897089 | 2.946245 | 0.660862 |
| H | 2.811904 | -0.787538 | 1.763563 | H | 2.218482 | -0.363201 | -1.066847 |
| H | 2.507501 | 0.442592 | 0.547759 | H | 2.702683 | -2.046165 | -0.887639 |
| H | 2.331721 | -2.577106 | 0.030398 | H | 2.197778 | -0.179907 | 1.489882 |
| H | 1.945666 | -1.318501 | -1.131825 | H | 2.54143 | -1.902289 | 1.566224 |
| H | 0.756965 | 1.59461 | -0.268953 | H | -0.003289 | 1.614619 | 1.569542 |
| H | -3.293353 | 3.155049 | -0.259275 | H | -3.402962 | 2.854181 | -0.819568 |
| H | -5.396164 | -1.974691 | -0.503433 | H | -4.860707 | -2.492092 | -1.2337 |
| H | -0.086518 | -4.627187 | -0.767505 | H | 0.649143 | -4.49156 | -0.092199 |
| **2b** (5) | | | | **2b** (6) | | | |
| C | -4.068577 | -0.948209 | -0.729478 | C | 2.064134 | 2.813503 | -0.895379 |
| C | -2.351088 | 0.7465 | -0.186027 | C | 2.250541 | 0.375195 | -0.527325 |
| C | -1.802318 | -1.676894 | -0.166075 | C | 0.385638 | 1.677932 | 0.47476 |
| C | -1.435857 | -0.342854 | 0.024524 | C | 1.054848 | 0.471396 | 0.262896 |
| C | -3.691434 | 0.384159 | -0.557179 | C | 2.738868 | 1.606534 | -1.084884 |
| C | -3.131419 | -1.955322 | -0.539193 | C | 0.906089 | 2.839508 | -0.129473 |
| C | 3.963572 | 0.743228 | -0.34535 | C | -4.020643 | -1.236552 | -0.76719 |
| C | -2.030939 | 2.177635 | -0.079447 | C | 2.987439 | -0.860247 | -0.835735 |
| C | 5.227684 | 0.441369 | -0.683813 | C | -4.947689 | -0.466376 | -1.35933 |
| C | -0.811377 | -2.804636 | 0.005183 | C | -0.850512 | 1.763978 | 1.342091 |
| C | 0.617659 | -2.268903 | 0.023469 | C | -1.010688 | 0.516591 | 2.214433 |
| C | 0.722667 | -1.046837 | 0.951525 | C | -0.76302 | -0.753583 | 1.375282 |
| C | 6.337694 | 1.449785 | -0.495086 | C | -5.651229 | -0.935574 | -2.612062 |
| C | 5.67757 | -0.875182 | -1.270682 | C | -5.386821 | 0.893788 | -0.872904 |
| C | 0.308649 | -1.358565 | 2.39452 | C | -0.760162 | -2.014064 | 2.236659 |
| C | -0.460084 | 2.8521 | 1.740312 | C | 3.158158 | -2.524162 | 1.018091 |
| C | -0.434411 | 4.094161 | -0.472245 | C | 3.023323 | -3.324195 | -1.386365 |
| C | 2.729014 | -0.114493 | -0.450568 | C | -3.212807 | -0.952723 | 0.473501 |
| C | 2.119379 | -0.402601 | 0.93605 | C | -1.701812 | -0.890867 | 0.157597 |
| C | -0.636903 | 2.733448 | 0.210364 | C | 2.563841 | -2.256673 | -0.382892 |
| O | -2.941569 | 3.028454 | -0.224202 | O | 4.047307 | -0.796795 | -1.503382 |
| O | -0.174989 | -0.02106 | 0.417932 | O | 0.615485 | -0.670213 | 0.865253 |
| O | -4.646636 | 1.303915 | -0.759768 | O | 3.855739 | 1.658948 | -1.825336 |
| O | -3.444398 | -3.267465 | -0.697501 | O | 0.216111 | 3.987709 | 0.092087 |
| O | 1.556964 | -3.238434 | 0.478686 | O | -0.14359 | 0.560333 | 3.34942 |

| | | | | | | | |
|---|---|---|---|---|---|---|---|
| H | -5.093631 | -1.170495 | -1.009339 | H | 2.465023 | 3.714388 | -1.349494 |
| H | 3.783855 | 1.735478 | 0.074402 | H | -3.798413 | -2.198926 | -1.232956 |
| H | -0.998887 | -3.363825 | 0.931751 | H | -0.792339 | 2.638329 | 1.99832 |
| H | -0.92382 | -3.525115 | -0.813964 | H | -1.745951 | 1.909133 | 0.722481 |
| H | 0.878402 | -1.934666 | -0.990331 | H | -2.019297 | 0.481938 | 2.634368 |
| H | 7.121018 | 1.055471 | 0.167617 | H | -6.738233 | -0.988418 | -2.457783 |
| H | 6.829559 | 1.679685 | -1.450869 | H | -5.491242 | -0.232099 | -3.44126 |
| H | 5.970171 | 2.388164 | -0.06748 | H | -5.304433 | -1.92351 | -2.931558 |
| H | 6.137014 | -0.721687 | -2.257164 | H | -5.235493 | 1.648792 | -1.656645 |
| H | 6.451188 | -1.333425 | -0.639049 | H | -6.46249 | 0.894481 | -0.648171 |
| H | 4.867807 | -1.599103 | -1.386835 | H | -4.857674 | 1.229148 | 0.022021 |
| H | 0.482605 | -0.48582 | 3.031678 | H | -0.523103 | -2.891498 | 1.627026 |
| H | 0.900472 | -2.194455 | 2.777959 | H | -1.743368 | -2.164723 | 2.692157 |
| H | -0.749642 | -1.625158 | 2.463378 | H | -0.025212 | -1.926263 | 3.038756 |
| H | -1.202198 | 3.541299 | 2.160034 | H | 4.253143 | -2.481425 | 0.984063 |
| H | 0.537009 | 3.244004 | 1.970478 | H | 2.867138 | -3.523425 | 1.360625 |
| H | -0.567343 | 1.882704 | 2.234851 | H | 2.803088 | -1.796518 | 1.75302 |
| H | -1.144358 | 4.838771 | -0.101883 | H | 4.11352 | -3.357585 | -1.466149 |
| H | -0.559491 | 4.019489 | -1.558287 | H | 2.617818 | -3.134954 | -2.386506 |
| H | 0.580812 | 4.454542 | -0.272289 | H | 2.670725 | -4.308403 | -1.058319 |
| H | 1.981721 | 0.409214 | -1.063945 | H | -3.537994 | -0.021729 | 0.947568 |
| H | 2.949097 | -1.057576 | -0.958854 | H | -3.397248 | -1.753399 | 1.203396 |
| H | 2.048039 | 0.533278 | 1.504873 | H | -1.520695 | -0.064847 | -0.538602 |
| H | 2.794091 | -1.058815 | 1.495971 | H | -1.408642 | -1.806807 | -0.37117 |
| H | 0.109824 | 2.034475 | -0.164031 | H | 1.477999 | -2.287947 | -0.300788 |
| H | -4.206605 | 2.189866 | -0.578262 | H | 4.199854 | 0.71447 | -1.856135 |
| H | -4.376129 | -3.349659 | -0.961544 | H | 0.670417 | 4.729189 | -0.342085 |
| H | 1.507563 | -3.997247 | -0.124886 | H | 0.769707 | 0.522307 | 3.018842 |
| **2b** (7) | | | | **2b** (8) | | | |
| C | -2.431552 | -0.514445 | -2.44276 | C | -4.145228 | -1.35719 | -0.337525 |
| C | -1.578953 | 0.795766 | -0.526172 | C | -2.611158 | 0.572573 | -0.13205 |
| C | -1.154419 | -1.648786 | -0.691834 | C | -1.740891 | -1.752989 | -0.132379 |
| C | -1.004353 | -0.426261 | -0.033993 | C | -1.534368 | -0.376641 | -0.040296 |
| C | -2.294679 | 0.700043 | -1.768493 | C | -3.931218 | 0.019968 | -0.26584 |
| C | -1.872095 | -1.665193 | -1.904358 | C | -3.060351 | -2.222334 | -0.277514 |
| C | 3.909913 | -0.145749 | -0.131419 | C | 4.510902 | 0.666444 | 0.373522 |
| C | -1.498669 | 2.115423 | 0.122303 | C | -2.475788 | 2.036249 | -0.133139 |
| C | 4.188879 | 0.673165 | -1.15869 | C | 5.611471 | 0.490548 | -0.3757 |
| C | -0.587586 | -2.936047 | -0.133674 | C | -0.588076 | -2.724438 | -0.089699 |
| C | -0.172067 | -2.775523 | 1.330482 | C | 0.748031 | -2.017476 | -0.310666 |
| C | 0.592488 | -1.446277 | 1.526191 | C | 0.837435 | -0.735779 | 0.540799 |
| C | 5.441895 | 0.478073 | -1.981114 | C | 6.582659 | 1.627029 | -0.599235 |
| C | 3.332205 | 1.836544 | -1.597741 | C | 6.000932 | -0.802178 | -1.05165 |

| | | | | | | | |
|---|---|---|---|---|---|---|---|
| C | 0.89191 | -1.192509 | 3.001678 | C | 0.717746 | -1.005734 | 2.042762 |
| C | -1.902913 | 1.951975 | 2.582145 | C | -0.731225 | 3.022641 | 1.355321 |
| C | -0.462185 | 3.829374 | 1.666677 | C | -1.241118 | 4.107327 | -0.880592 |
| C | 2.723267 | -0.118659 | 0.797213 | C | 3.433609 | -0.332149 | 0.711317 |
| C | 1.85734 | -1.387289 | 0.644948 | C | 2.058809 | 0.138686 | 0.191832 |
| C | -0.880895 | 2.363116 | 1.497476 | C | -1.141004 | 2.780203 | -0.114048 |
| O | -2.004178 | 3.113148 | -0.444451 | O | -3.504722 | 2.754134 | -0.147877 |
| O | -0.324377 | -0.361876 | 1.146515 | O | -0.283364 | 0.123477 | 0.132961 |
| O | -2.865913 | 1.767197 | -2.345907 | O | -5.023218 | 0.796326 | -0.333128 |
| O | -1.985092 | -2.872855 | -2.514495 | O | -3.210091 | -3.570282 | -0.349778 |
| O | -1.303051 | -2.857367 | 2.199653 | O | 1.840098 | -2.867125 | 0.032625 |
| H | -2.97888 | -0.534405 | -3.380039 | H | -5.161443 | -1.725341 | -0.439261 |
| H | 4.618442 | -0.952082 | 0.070343 | H | 4.352661 | 1.659967 | 0.799706 |
| H | -1.334511 | -3.734414 | -0.193188 | H | -0.549891 | -3.254951 | 0.871541 |
| H | 0.266417 | -3.275048 | -0.734613 | H | -0.72355 | -3.496266 | -0.857152 |
| H | 0.475687 | -3.604715 | 1.62947 | H | 0.82514 | -1.706709 | -1.363713 |
| H | 5.197179 | 0.307532 | -3.039043 | H | 7.58618 | 1.36755 | -0.233009 |
| H | 6.07701 | 1.374827 | -1.952486 | H | 6.69319 | 1.847151 | -1.670533 |
| H | 6.035989 | -0.370734 | -1.627574 | H | 6.263278 | 2.544404 | -0.094258 |
| H | 2.417906 | 1.950037 | -1.010883 | H | 6.118337 | -0.650531 | -2.133659 |
| H | 3.895288 | 2.777956 | -1.531525 | H | 6.974946 | -1.153708 | -0.683137 |
| H | 3.04347 | 1.724327 | -2.651909 | H | 5.276012 | -1.605339 | -0.90154 |
| H | 1.266597 | -0.177971 | 3.15793 | H | 0.871746 | -0.081021 | 2.607909 |
| H | 1.652674 | -1.898904 | 3.350903 | H | 1.466221 | -1.737442 | 2.357763 |
| H | -0.010209 | -1.33231 | 3.599983 | H | -0.270676 | -1.39949 | 2.296448 |
| H | -2.816163 | 2.552848 | 2.497434 | H | -1.479259 | 3.641212 | 1.86495 |
| H | -1.475922 | 2.122346 | 3.576626 | H | 0.228751 | 3.549824 | 1.392165 |
| H | -2.173379 | 0.895402 | 2.505946 | H | -0.625193 | 2.08291 | 1.904399 |
| H | -1.322378 | 4.502898 | 1.614985 | H | -1.975746 | 4.777822 | -0.426254 |
| H | 0.250195 | 4.134869 | 0.892646 | H | -1.531664 | 3.945728 | -1.924743 |
| H | 0.01919 | 3.95962 | 2.642337 | H | -0.265906 | 4.606773 | -0.874084 |
| H | 3.088555 | -0.049305 | 1.830578 | H | 3.655396 | -1.323342 | 0.31399 |
| H | 2.103308 | 0.766032 | 0.624522 | H | 3.38403 | -0.437274 | 1.804708 |
| H | 2.46419 | -2.272883 | 0.877175 | H | 2.10124 | 0.246303 | -0.899468 |
| H | 1.568577 | -1.477441 | -0.407047 | H | 1.853147 | 1.138412 | 0.59543 |
| H | -0.003686 | 1.726237 | 1.607345 | H | -0.379469 | 2.156906 | -0.58084 |
| H | -2.654828 | 2.542646 | -1.741661 | H | -4.685 | 1.74003 | -0.253151 |
| H | -2.511937 | -2.778742 | -3.325848 | H | -4.151007 | -3.786782 | -0.460889 |
| H | -1.883079 | -2.105051 | 1.993875 | H | 1.791889 | -3.647582 | -0.54305 |
| **2b** (9) | | | | **2b** (10) | | | |
| C | -4.323037 | -0.88575 | -0.038442 | C | 4.14518 | -1.357368 | -0.337597 |
| C | -2.501581 | 0.786584 | -0.061061 | C | 2.611227 | 0.572466 | -0.131972 |
| C | -1.998387 | -1.644704 | -0.030535 | C | 1.740828 | -1.75305 | -0.132301 |

| | | | | | | | |
|---|---|---|---|---|---|---|---|
| C | -1.578345 | -0.314955 | -0.051148 | C | 1.534394 | -0.376687 | -0.040188 |
| C | -3.896925 | 0.442154 | -0.083807 | C | 3.931266 | 0.019794 | -0.265844 |
| C | -3.38152 | -1.906735 | -0.00567 | C | 3.060251 | -2.222456 | -0.277551 |
| C | 4.545606 | -0.300993 | -0.655179 | C | -4.510862 | 0.666712 | 0.373275 |
| C | -2.14696 | 2.212414 | -0.011303 | C | 2.475943 | 2.036132 | -0.133044 |
| C | 5.568396 | 0.37218 | -0.103269 | C | -5.611537 | 0.490576 | -0.375733 |
| C | -1.008077 | -2.781828 | -0.067825 | C | 0.587942 | -2.724425 | -0.089576 |
| C | 0.376473 | -2.309388 | -0.509631 | C | -0.748085 | -2.017355 | -0.310648 |
| C | 0.7728 | -1.007917 | 0.216139 | C | -0.837469 | -0.735667 | 0.540842 |
| C | 6.627728 | 1.012885 | -0.97051 | C | -6.582726 | 1.626999 | -0.599552 |
| C | 5.782825 | 0.56114 | 1.379505 | C | -6.001129 | -0.802404 | -1.051119 |
| C | 0.825303 | -1.161689 | 1.738146 | C | -0.717908 | -1.005664 | 2.042809 |
| C | -0.722954 | 4.060135 | 0.951094 | C | 1.24146 | 4.107197 | -0.880789 |
| C | -0.097729 | 2.940955 | -1.239404 | C | 0.73132 | 3.022826 | 1.355214 |
| C | 3.399238 | -1.002864 | 0.026778 | C | -3.433591 | -0.331812 | 0.711327 |
| C | 2.049503 | -0.358753 | -0.355474 | C | -2.058759 | 0.138879 | 0.19178 |
| C | -0.724774 | 2.744448 | 0.158478 | C | 1.141212 | 2.780167 | -0.114084 |
| O | -3.048887 | 3.078844 | -0.111693 | O | 3.504931 | 2.753966 | -0.147706 |
| O | -0.255214 | -0.011862 | -0.118387 | O | 0.283438 | 0.123522 | 0.133152 |
| O | -4.858872 | 1.376058 | -0.130377 | O | 5.023276 | 0.796114 | -0.333149 |
| O | -3.741405 | -3.21586 | 0.032557 | O | 3.209936 | -3.570403 | -0.349874 |
| O | 1.364512 | -3.300329 | -0.237212 | O | -1.840249 | -2.866923 | 0.032536 |
| H | -5.388356 | -1.094673 | -0.038599 | H | 5.161361 | -1.725587 | -0.43942 |
| H | 4.51578 | -0.352597 | -1.746027 | H | -4.352522 | 1.660389 | 0.799065 |
| H | -0.917019 | -3.259722 | 0.917209 | H | 0.723392 | -3.496331 | -0.856947 |
| H | -1.362416 | -3.562446 | -0.751844 | H | 0.549669 | -3.254842 | 0.871711 |
| H | 0.355273 | -2.07947 | -1.585752 | H | -0.825082 | -1.706552 | -1.363687 |
| H | 6.682834 | 2.096098 | -0.791551 | H | -6.693477 | 1.846671 | -1.670918 |
| H | 7.624557 | 0.611467 | -0.738929 | H | -7.58618 | 1.367714 | -0.233003 |
| H | 6.436026 | 0.853239 | -2.036486 | H | -6.263209 | 2.544574 | -0.095026 |
| H | 6.747343 | 0.134175 | 1.688119 | H | -6.974985 | -1.1539 | -0.68215 |
| H | 5.826025 | 1.630519 | 1.628825 | H | -6.118929 | -0.65112 | -2.133135 |
| H | 5.002963 | 0.105144 | 1.993737 | H | -5.276088 | -1.605455 | -0.901 |
| H | 1.175391 | -0.232875 | 2.199733 | H | -0.871954 | -0.080974 | 2.60798 |
| H | 1.507594 | -1.969685 | 2.014707 | H | 0.270503 | -1.399413 | 2.296548 |
| H | -0.161656 | -1.395028 | 2.148092 | H | -1.466401 | -1.737385 | 2.357743 |
| H | -1.280469 | 4.842914 | 0.429509 | H | 1.976087 | 4.777706 | -0.426467 |
| H | 0.308833 | 4.402736 | 1.087544 | H | 0.266275 | 4.6067 | -0.874418 |
| H | -1.170406 | 3.9308 | 1.943022 | H | 1.532085 | 3.945459 | -1.924895 |
| H | -0.678314 | 3.66671 | -1.820831 | H | 1.479306 | 3.641495 | 1.864796 |
| H | -0.054098 | 2.001134 | -1.796568 | H | 0.62527 | 2.083185 | 1.904441 |
| H | 0.923836 | 3.32422 | -1.138321 | H | -0.228664 | 3.55 | 1.391887 |
| H | 3.384935 | -2.056642 | -0.272725 | H | -3.384036 | -0.43667 | 1.804744 |

| | | | | | | | |
|---|---|---|---|---|---|---|---|
| H | 3.521044 | -0.988078 | 1.114033 | H | -3.655376 | -1.323093 | 0.31422 |
| H | 1.953909 | -0.330176 | -1.448966 | H | -1.853023 | 1.138618 | 0.595302 |
| H | 2.053407 | 0.68483 | -0.01796 | H | -2.101189 | 0.24641 | -0.899527 |
| H | -0.131436 | 2.005403 | 0.695228 | H | 0.379669 | 2.156874 | -0.580867 |
| H | -4.373988 | 2.256449 | -0.161727 | H | 4.685083 | 1.739847 | -0.253096 |
| H | -4.710689 | -3.287113 | 0.024355 | H | 4.150842 | -3.786932 | -0.461012 |
| H | 1.122528 | -4.096041 | -0.738239 | H | -1.792818 | -3.64676 | -0.544038 |
| **2b** (11) | | | | | | | |
| C | -4.353333 | -0.400778 | -0.016612 | | | | |
| C | -2.287939 | 0.962236 | 0.003796 | | | | |
| C | -2.178395 | -1.517136 | -0.079397 | | | | |
| C | -1.548046 | -0.272172 | -0.036913 | | | | |
| C | -3.721155 | 0.842685 | -0.001396 | | | | |
| C | -3.585701 | -1.55669 | -0.052997 | | | | |
| C | 4.090238 | 0.435606 | -0.410084 | | | | |
| C | -1.717628 | 2.316093 | 0.071091 | | | | |
| C | 5.380645 | 0.258668 | -0.082899 | | | | |
| C | -1.394829 | -2.802797 | -0.181361 | | | | |
| C | 0.05028 | -2.540981 | -0.595231 | | | | |
| C | 0.646259 | -1.35922 | 0.196677 | | | | |
| C | 6.480976 | 0.710984 | -1.015162 | | | | |
| C | 5.871949 | -0.379137 | 1.194414 | | | | |
| C | 0.661043 | -1.611131 | 1.706467 | | | | |
| C | 0.055793 | 3.949752 | 0.825676 | | | | |
| C | 0.32128 | 2.64149 | -1.332359 | | | | |
| C | 2.858145 | 0.054077 | 0.369971 | | | | |
| C | 2.034387 | -1.016155 | -0.373852 | | | | |
| C | -0.222568 | 2.618842 | 0.11388 | | | | |
| O | -2.48012 | 3.312588 | 0.074498 | | | | |
| O | -0.189915 | -0.185035 | -0.078704 | | | | |
| O | -4.525966 | 1.915439 | 0.020282 | | | | |
| O | -4.14906 | -2.792339 | -0.078025 | | | | |
| O | 0.876503 | -3.680364 | -0.367949 | | | | |
| H | -5.438336 | -0.437839 | -0.006331 | | | | |
| H | 3.880574 | 0.906861 | -1.37293 | | | | |
| H | -1.397315 | -3.345399 | 0.773657 | | | | |
| H | -1.870001 | -3.470268 | -0.910104 | | | | |
| H | 0.076966 | -2.258863 | -1.659027 | | | | |
| H | 7.137494 | 1.443749 | -0.524948 | | | | |
| H | 7.123428 | -0.132132 | -1.306293 | | | | |
| H | 6.083244 | 1.167124 | -1.927401 | | | | |
| H | 6.503932 | -1.249912 | 0.971114 | | | | |
| H | 6.502698 | 0.321795 | 1.758861 | | | | |

| | | | | | | | |
|---|---|---|---|---|---|---|---|
| H | 5.06655 | -0.710988 | 1.85375 | | | | |
| H | 1.053083 | -0.739941 | 2.237092 | | | | |
| H | 1.292813 | -2.474495 | 1.9337 | | | | |
| H | -0.343956 | -1.811365 | 2.088019 | | | | |
| H | -0.398585 | 4.791051 | 0.295149 | | | | |
| H | 1.137583 | 4.116828 | 0.875941 | | | | |
| H | -0.335137 | 3.946443 | 1.849116 | | | | |
| H | -0.17468 | 3.426398 | -1.915527 | | | | |
| H | 0.169632 | 1.683068 | -1.836044 | | | | |
| H | 1.396038 | 2.854563 | -1.322185 | | | | |
| H | 3.122272 | -0.305793 | 1.368409 | | | | |
| H | 2.240373 | 0.950442 | 0.513892 | | | | |
| H | 2.607926 | -1.94808 | -0.405369 | | | | |
| H | 1.888657 | -0.703694 | -1.416121 | | | | |
| H | 0.278269 | 1.815085 | 0.65212 | | | | |
| H | -3.910185 | 2.71111 | 0.03424 | | | | |
| H | -5.117137 | -2.706877 | -0.078818 | | | | |
| H | 0.523863 | -4.40659 | -0.907034 | | | | |

Key transitions, oscillator strengths, and rotatory strengths in the ECD of conformers **2b** (5) at B3LYP /6-311++G(d,p) level.

| Species | Excited State | *ΔE(eV)*[a] | *λ(nm)*[b] | *f*[c] | $R_{vel}$[d] |
|---|---|---|---|---|---|
| **2b** (5) | 92 -> 97 | 3.7269 | 332.67 | 0.076 | 5.1071 |
| | 91 ->108 | 4.1589 | 298.12 | 0.0011 | -0.2518 |
| | 91 -> 95 | 4.2772 | 289.87 | 0.0188 | -4.1331 |
| | 84 -> 95 | 4.4623 | 277.85 | 0.3639 | 6.7243 |
| | 94 -> 99 | 5.2236 | 237.36 | 0.003 | -0.6586 |
| | 89 -> 95 | 5.4583 | 227.15 | 0.0631 | 1.2971 |
| | 93 -> 98 | 5.4769 | 226.37 | 0.0051 | -2.6545 |
| | 94 -> 98 | 5.5539 | 223.24 | 0.0055 | -0.1919 |
| | 94 -> 99 | 5.5662 | 222.74 | 0.0101 | -3.9368 |
| | 94 -> 97 | 5.5937 | 221.65 | 0.0272 | 6.4633 |
| | 92 -> 97 | 5.6289 | 220.26 | 0.0131 | 3.5137 |
| | 94 -> 99 | 5.6495 | 219.46 | 0.0044 | -4.4822 |
| | 94 ->100 | 5.7884 | 214.2 | 0.2025 | 61.3966 |
| | 94 ->100 | 5.822 | 212.96 | 0.0047 | -7.9166 |
| | 94 ->101 | 5.8463 | 212.07 | 0.0792 | -41.1861 |
| | 93 -> 99 | 5.8861 | 210.64 | 0.0046 | -3.7936 |
| | 93 ->100 | 5.9038 | 210.01 | 0.021 | -8.0402 |
| | 87 -> 95 | 5.9134 | 209.67 | 0.0128 | -21.5855 |
| | 93 -> 97 | 5.952 | 208.31 | 0.0274 | -6.0836 |
| | 93 -> 97 | 5.9661 | 207.82 | 0.0268 | 2.7626 |
| | 93 ->101 | 6.0398 | 205.28 | 0.036 | -0.0051 |

| | | | | | |
|---|---|---|---|---|---|
| | 93 ->100 | 6.0558 | 204.74 | 0.0027 | 11.3979 |
| | 93 ->100 | 6.0647 | 204.44 | 0.0437 | 5.1613 |
| | 94 ->102 | 6.1164 | 202.71 | 0.0003 | -0.8966 |
| | 94 ->103 | 6.1368 | 202.03 | 0.0159 | -2.3557 |
| | 94 ->104 | 6.1608 | 201.25 | 0.0197 | 10.3733 |
| | 94 ->105 | 6.256 | 198.19 | 0.0293 | 25.3519 |
| | 94 ->104 | 6.2947 | 196.97 | 0.1071 | -12.3992 |
| | 94 ->105 | 6.3085 | 196.54 | 0.0269 | -19.2831 |
| | 91 -> 97 | 6.3101 | 196.48 | 0.0697 | 3.1114 |
| | 93 ->107 | 6.3561 | 195.06 | 0.117 | -32.8891 |
| | 94 ->105 | 6.3806 | 194.31 | 0.0836 | -18.3998 |
| | 86 -> 95 | 6.416 | 193.24 | 0.0175 | -16.5408 |
| | 94 ->106 | 6.4229 | 193.04 | 0.1149 | -17.0325 |
| | 91 -> 99 | 6.4407 | 192.5 | 0.0133 | 45.8503 |
| | 93 ->103 | 6.4605 | 191.91 | 0.0585 | 61.9068 |
| | 92 ->105 | 6.4858 | 191.16 | 0.031 | -26.9348 |
| | 94 ->114 | 6.4903 | 191.03 | 0.0948 | -30.2598 |
| | 93 ->106 | 6.5273 | 189.95 | 0.0063 | 10.1916 |
| | 93 ->105 | 6.5396 | 189.59 | 0.0192 | 12.5553 |

[a]Excitation energy. [b]Wavelength. [c]Oscillator strength. [d]Rotatory strength in velocity form ($10^{-40}$cgs.).

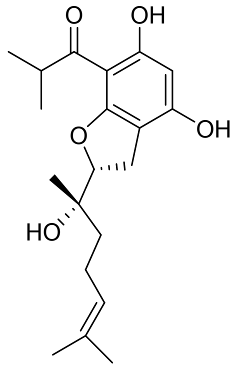


**3b**

Optimized geometries of predominant conformers for compound **3b** at the B3LYP/6-31G (d,p) level in methanol solution.

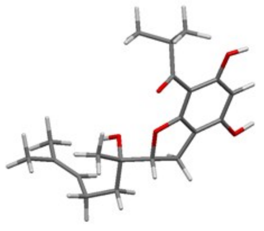
**3b** (1)

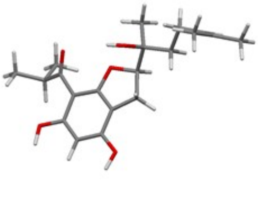
**3b** (2)

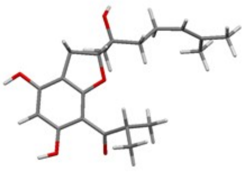
**3b** (3)

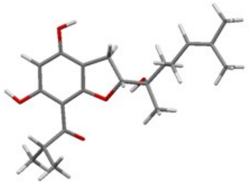
**3b** (4)

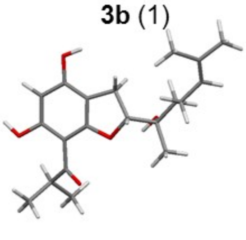
**3b** (5)

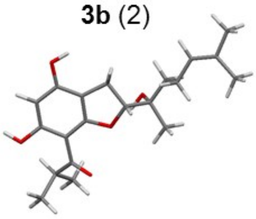
**3b** (6)

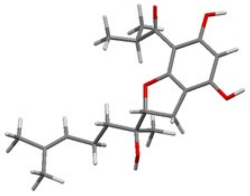
**3b** (7)

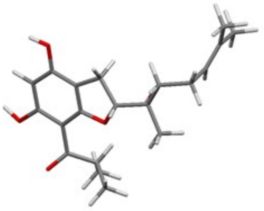
**3b** (8)

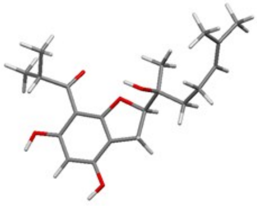
**3b** (9)

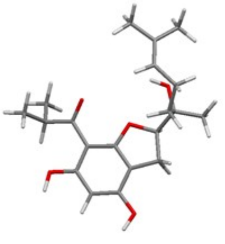
**3b** (10)

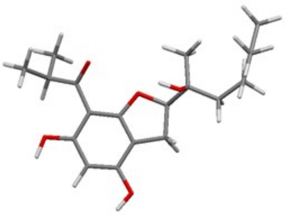
**3b** (11)

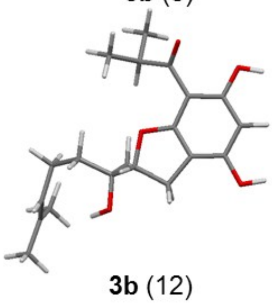
**3b** (12)

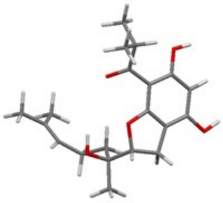
**3b** (13)

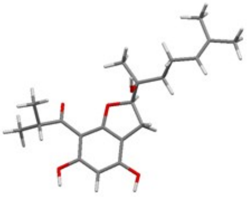
**3b** (14)

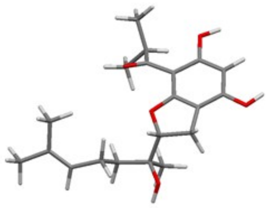
**3b** (15)

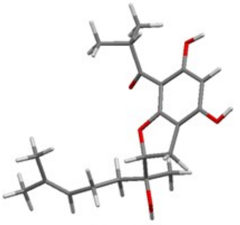
**3b** (16)

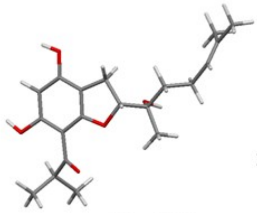
**3b** (17)

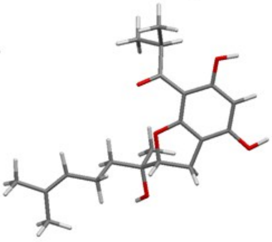
**3b** (18)

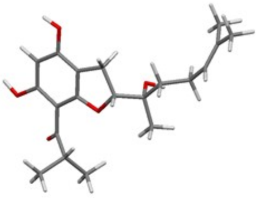
**3b** (19)

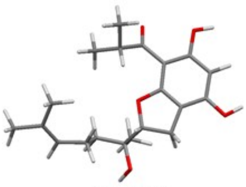
**3b** (20)

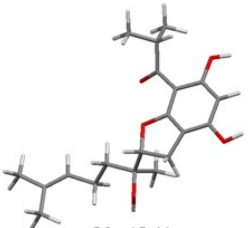
**3b** (21)

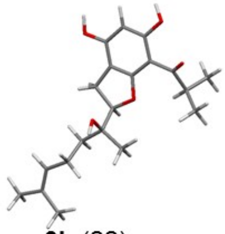
**3b** (22)

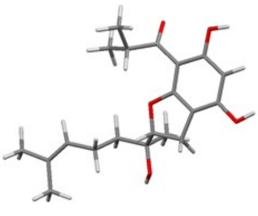
**3b** (23)

Important thermodynamic parameters (a.u.) and Boltzmann distributions of the optimized compound **3b** at B3LYP/6-31G(d,p) level in methanol solution.

| Conformations | E+ZPE | G | % |
|---|---|---|---|
| **3b** (1) | -1154.651026 | -1154.709271 | 2.71% |
| **3b** (2) | -1154.652849 | -1154.711691 | 35.14% |
| **3b** (3) | -1154.649200 | -1154.708913 | 1.86% |
| **3b** (4) | -1154.651495 | -1154.710475 | 9.70% |
| **3b** (5) | -1154.651179 | -1154.710562 | 10.63% |
| **3b** (6) | -1154.651071 | -1154.709747 | 4.49% |
| **3b** (7) | -1154.648744 | -1154.708680 | 1.45% |
| **3b** (8) | -1154.649054 | -1154.708471 | 1.16% |
| **3b** (9) | -1154.649967 | -1154.709170 | 2.44% |
| **3b** (10) | -1154.649569 | -1154.708504 | 1.20% |
| **3b** (11) | -1154.650086 | -1154.708437 | 1.12% |
| **3b** (12) | -1154.650153 | -1154.708681 | 1.45% |
| **3b** (13) | -1154.649131 | -1154.708671 | 1.44% |
| **3b** (14) | -1154.649662 | -1154.708576 | 1.30% |
| **3b** (15) | -1154.649867 | -1154.710090 | 6.45% |
| **3b** (16) | -1154.649849 | -1154.708931 | 1.89% |
| **3b** (17) | -1154.649875 | -1154.709039 | 2.12% |
| **3b** (18) | -1154.649701 | -1154.709618 | 3.91% |
| **3b** (19) | -1154.649182 | -1154.709043 | 2.13% |
| **3b** (20) | -1154.649396 | -1154.708644 | 1.40% |
| **3b** (21) | -1154.649366 | -1154.708570 | 1.29% |
| **3b** (22) | -1154.649210 | -1154.708434 | 1.12% |
| **3b** (23) | -1154.649407 | -1154.709542 | 3.61% |

E+ZPE, G: total energy with zero point energy (ZPE) and Gibbs free energy in methanol solution at B3LYP/6-31G (d,p) level. %: Boltzmann distributions, using the relative Gibbs free energies as weighting factors.

Optimized Z-matrixes of compound **2b** in methanol solution (Å) at B3LYP/6-31G (d,p) level.

| **3b** (1) | | | | **3b** (2) | | | |
|---|---|---|---|---|---|---|---|
| C | 3.160313 | 2.29046 | 0.892372 | C | 2.915794 | 2.752421 | 0.102459 |
| C | 2.665022 | 0.080692 | -0.089364 | C | 2.871899 | 0.285767 | 0.131627 |
| C | 1.034283 | 1.941004 | -0.194174 | C | 0.837778 | 1.612855 | -0.350673 |
| C | 1.429037 | 0.638864 | -0.486688 | C | 1.495954 | 0.402505 | -0.159062 |
| C | 3.524718 | 0.974344 | 0.603794 | C | 3.555673 | 1.523093 | 0.271128 |
| C | 1.900461 | 2.772824 | 0.505518 | C | 1.548214 | 2.799855 | -0.20889 |
| C | -4.863988 | 0.299618 | 0.412904 | C | -5.617112 | -0.611135 | -0.488236 |
| C | 2.99631 | -1.335187 | -0.404857 | C | 3.503156 | -1.053034 | 0.2841 |
| C | -5.322988 | -0.762053 | 1.102715 | C | -6.588299 | -0.002372 | 0.211745 |
| C | -0.374272 | 2.159008 | -0.675834 | C | -0.618694 | 1.348159 | -0.626689 |
| C | -0.67434 | 0.82114 | -1.404294 | C | -0.615282 | -0.181058 | -0.862823 |
| C | -5.586153 | -0.660422 | 2.587521 | C | -7.829727 | 0.513593 | -0.479162 |
| C | -5.643262 | -2.111241 | 0.505516 | C | -6.567348 | 0.22675 | 1.704011 |
| C | 5.156475 | -2.587055 | -0.076449 | C | 4.925104 | -2.417193 | -1.252743 |
| C | 2.998918 | -3.423522 | 0.970167 | C | 5.835867 | -1.675935 | 1.000334 |

| | | | | | | | |
|---|---|---|---|---|---|---|---|
| C | -2.024852 | -1.299645 | -1.603413 | C | -1.52514 | -2.491885 | -0.430043 |
| C | -4.57578 | 0.417463 | -1.064321 | C | -4.316129 | -1.186056 | 0.008568 |
| C | -3.172239 | 0.97613 | -1.394256 | C | -3.102416 | -0.491914 | -0.643907 |
| C | 3.822016 | -2.183486 | 0.575141 | C | 4.942076 | -1.298672 | -0.194237 |
| C | -1.966649 | 0.1002 | -0.969665 | C | -1.727563 | -0.991044 | -0.161631 |
| O | 2.56002 | -1.866893 | -1.424115 | O | 2.856959 | -1.997115 | 0.734176 |
| O | 0.47344 | -0.053728 | -1.149998 | O | 0.669423 | -0.663636 | -0.335488 |
| O | 4.766827 | 0.520322 | 0.940776 | O | 4.871746 | 1.477595 | 0.626388 |
| O | 1.483766 | 4.038033 | 0.779999 | O | 0.87425 | 3.968538 | -0.375004 |
| O | -1.88731 | -0.014555 | 0.45829 | O | -1.662764 | -0.734473 | 1.248989 |
| H | 3.861633 | 2.944311 | 1.405173 | H | 3.475626 | 3.675415 | 0.234585 |
| H | -4.697208 | 1.221556 | 0.974504 | H | -5.76655 | -0.707344 | -1.566207 |
| H | -1.065033 | 2.313743 | 0.159908 | H | -1.242298 | 1.6105 | 0.236809 |
| H | -0.469449 | 3.014737 | -1.352235 | H | -1.003395 | 1.888368 | -1.496612 |
| H | -0.70551 | 0.961074 | -2.490154 | H | -0.61293 | -0.409313 | -1.935375 |
| H | -5.340614 | 0.330857 | 2.98112 | H | -7.805815 | 0.327703 | -1.557787 |
| H | -6.642276 | -0.8656 | 2.810959 | H | -8.734603 | 0.040982 | -0.071364 |
| H | -5.001545 | -1.406073 | 3.14343 | H | -7.949129 | 1.594879 | -0.321115 |
| H | -5.422666 | -2.18011 | -0.561971 | H | -5.657876 | -0.138 | 2.18666 |
| H | -6.708327 | -2.34335 | 0.642748 | H | -7.42425 | -0.267616 | 2.182783 |
| H | -5.085291 | -2.904319 | 1.020983 | H | -6.663965 | 1.297569 | 1.93101 |
| H | 5.765067 | -1.707637 | -0.310574 | H | 4.313318 | -2.140628 | -2.1195 |
| H | 4.975557 | -3.141258 | -1.004145 | H | 4.519089 | -3.341794 | -0.830631 |
| H | 5.73155 | -3.229116 | 0.600538 | H | 5.942803 | -2.614912 | -1.60765 |
| H | 2.062678 | -3.142284 | 1.466564 | H | 5.890979 | -0.858854 | 1.72702 |
| H | 3.572297 | -4.051358 | 1.661497 | H | 6.85329 | -1.897695 | 0.65867 |
| H | 2.749556 | -4.018756 | 0.086193 | H | 5.441796 | -2.564215 | 1.506139 |
| H | -2.08803 | -1.241147 | -2.696495 | H | -1.641307 | -2.723646 | -1.495122 |
| H | -2.899329 | -1.850213 | -1.243067 | H | -2.253454 | -3.08049 | 0.133871 |
| H | -1.131083 | -1.86746 | -1.335535 | H | -0.522131 | -2.80352 | -0.123253 |
| H | -4.734775 | -0.533591 | -1.580997 | H | -4.22844 | -1.107833 | 1.095434 |
| H | -5.301514 | 1.119695 | -1.501065 | H | -4.291898 | -2.258123 | -0.232057 |
| H | -3.070183 | 1.96066 | -0.920596 | H | -3.174151 | 0.583519 | -0.443875 |
| H | -3.117499 | 1.14117 | -2.478029 | H | -3.15199 | -0.614227 | -1.734239 |
| H | 4.033248 | -1.600057 | 1.473292 | H | 5.333599 | -0.387724 | -0.650954 |
| H | 5.247482 | 1.219812 | 1.413471 | H | 5.222638 | 2.38162 | 0.684116 |
| H | 2.175981 | 4.511269 | 1.271065 | H | 1.481074 | 4.717399 | -0.251008 |
| H | -2.766832 | -0.296801 | 0.769797 | H | -0.769771 | -0.997479 | 1.531893 |
| **3b** (3) | | | | **3b** (4) | | | |
| C | -4.41381 | -0.744021 | 0.45974 | C | 3.071022 | 2.660175 | -0.184958 |
| C | -2.534757 | 0.83138 | 0.185028 | C | 2.858652 | 0.209772 | 0.010256 |
| C | -2.286517 | -1.582569 | -0.310747 | C | 0.868805 | 1.685276 | -0.0173 |
| C | -1.786551 | -0.287731 | -0.231844 | C | 1.468185 | 0.430479 | 0.07593 |

| | | | | | | | |
|---|---|---|---|---|---|---|---|
| C | -3.882186 | 0.545501 | 0.546749 | C | 3.641813 | 1.390836 | -0.111919 |
| C | -3.613059 | -1.814865 | 0.035675 | C | 1.67758 | 2.811613 | -0.135534 |
| C | 4.855152 | -0.329336 | -0.388213 | C | -5.257921 | 0.387437 | -0.480435 |
| C | -1.973319 | 2.207733 | 0.269009 | C | 3.409562 | -1.171173 | 0.078381 |
| C | 5.608486 | 0.584089 | 0.245547 | C | -6.473266 | -0.173646 | -0.587451 |
| C | -1.198309 | -2.522531 | -0.752578 | C | -0.62715 | 1.526915 | 0.110533 |
| C | 0.008116 | -1.561347 | -0.921459 | C | -0.784279 | -0.003331 | -0.050957 |
| C | 6.646401 | 1.383433 | -0.508326 | C | -7.721623 | 0.67667 | -0.533003 |
| C | 5.521493 | 0.911004 | 1.717089 | C | -6.734381 | -1.649509 | -0.769954 |
| C | 0.323364 | 3.165356 | -0.142342 | C | 4.307297 | -2.739533 | -1.649995 |
| C | -1.650432 | 3.823228 | -1.60079 | C | 5.808023 | -1.920068 | 0.229175 |
| C | 0.972093 | -1.878473 | 1.422156 | C | -1.738367 | -2.221525 | 0.695756 |
| C | 3.773399 | -1.213641 | 0.175904 | C | -3.906958 | -0.281354 | -0.504304 |
| C | 2.402255 | -0.918611 | -0.466377 | C | -3.172793 | -0.117757 | 0.842818 |
| C | -0.980849 | 2.688243 | -0.801266 | C | 4.658499 | -1.557453 | -0.728135 |
| C | 1.265293 | -1.895135 | -0.083155 | C | -1.749702 | -0.701964 | 0.928309 |
| O | -2.328912 | 2.992916 | 1.143841 | O | 2.833845 | -2.037717 | 0.73348 |
| O | -0.463703 | -0.224134 | -0.555005 | O | 0.550342 | -0.568126 | 0.18615 |
| O | -4.670958 | 1.581836 | 0.939984 | O | 4.998111 | 1.246882 | -0.111551 |
| O | -4.076128 | -3.090061 | -0.051462 | O | 1.187535 | 4.07832 | -0.204098 |
| O | 1.589341 | -3.22755 | -0.521005 | O | -1.317639 | -0.422111 | 2.269288 |
| H | -5.455288 | -0.915545 | 0.722495 | H | 3.697262 | 3.544555 | -0.258777 |
| H | 5.023023 | -0.457852 | -1.459729 | H | -5.218339 | 1.470508 | -0.345853 |
| H | -0.992203 | -3.311545 | -0.022181 | H | -0.984528 | 1.853047 | 1.095418 |
| H | -1.433462 | -3.023529 | -1.697925 | H | -1.199875 | 2.062138 | -0.654548 |
| H | 0.309789 | -1.502064 | -1.971682 | H | -1.04201 | -0.256978 | -1.085002 |
| H | 7.648566 | 1.230175 | -0.083485 | H | -7.488836 | 1.738137 | -0.400627 |
| H | 6.681281 | 1.112959 | -1.568561 | H | -8.313098 | 0.568398 | -1.4531 |
| H | 6.442965 | 2.461307 | -0.437039 | H | -8.376232 | 0.364443 | 0.292916 |
| H | 6.48493 | 0.724541 | 2.211837 | H | -7.36744 | -2.030903 | 0.043153 |
| H | 4.756712 | 0.338292 | 2.246786 | H | -5.824217 | -2.252873 | -0.796606 |
| H | 5.303145 | 1.97822 | 1.861339 | H | -7.287476 | -1.831504 | -1.701895 |
| H | 0.115902 | 3.960742 | 0.581285 | H | 3.964422 | -3.597991 | -1.064103 |
| H | 1.012946 | 3.556948 | -0.898608 | H | 5.188746 | -3.041703 | -2.226587 |
| H | 0.825718 | 2.345901 | 0.383675 | H | 3.514892 | -2.47303 | -2.359239 |
| H | -2.568574 | 3.481163 | -2.092692 | H | 6.688481 | -2.241041 | -0.339097 |
| H | -0.969111 | 4.19147 | -2.376002 | H | 5.508863 | -2.740081 | 0.891405 |
| H | -1.90768 | 4.657802 | -0.940347 | H | 6.095053 | -1.062906 | 0.846644 |
| H | 0.722937 | -0.868743 | 1.761765 | H | -2.036308 | -2.48012 | -0.325776 |
| H | 1.848132 | -2.214795 | 1.987453 | H | -2.429945 | -2.706015 | 1.392631 |
| H | 0.142831 | -2.546287 | 1.673901 | H | -0.735265 | -2.626001 | 0.863389 |
| H | 4.039405 | -2.261963 | -0.02948 | H | -3.99796 | -1.342324 | -0.753796 |
| H | 3.712097 | -1.113369 | 1.263394 | H | -3.307541 | 0.174438 | -1.306386 |

| | | | | | | | |
|---|---|---|---|---|---|---|---|
| H | 2.512756 | -0.953172 | -1.558708 | H | -3.762322 | -0.600288 | 1.632015 |
| H | 2.095799 | 0.102708 | -0.211898 | H | -3.125838 | 0.946582 | 1.105035 |
| H | -0.753433 | 1.867428 | -1.48415 | H | 4.969561 | -0.710814 | -1.34337 |
| H | -5.576071 | 1.253618 | 1.06958 | H | 5.41365 | 2.118867 | -0.214894 |
| H | -5.006692 | -3.121415 | 0.226534 | H | 0.21806 | 4.057778 | -0.149271 |
| H | 2.248875 | -3.587298 | 0.092823 | H | -0.397312 | -0.733458 | 2.326889 |
| **3b** (5) | | | | **3b** (6) | | | |
| C | 2.536017 | 2.824747 | 0.257941 | C | 3.033681 | 2.696412 | -0.043538 |
| C | 2.702728 | 0.413295 | -0.238999 | C | 2.838708 | 0.248119 | -0.284996 |
| C | 0.519719 | 1.498038 | 0.202658 | C | 0.841829 | 1.685621 | 0.000905 |
| C | 1.298347 | 0.388634 | -0.121697 | C | 1.446084 | 0.445144 | -0.19636 |
| C | 3.291362 | 1.694288 | -0.049168 | C | 3.611839 | 1.439973 | -0.215414 |
| C | 1.145285 | 2.724716 | 0.40604 | C | 1.643174 | 2.820341 | 0.087743 |
| C | -5.398794 | -0.820081 | -0.137329 | C | -5.299866 | 0.446894 | -0.221078 |
| C | 3.46245 | -0.828846 | -0.553047 | C | 3.407229 | -1.119468 | -0.444434 |
| C | -6.178881 | 0.217221 | -0.481797 | C | -6.520911 | -0.091858 | -0.369796 |
| C | -0.915223 | 1.061743 | 0.373371 | C | -0.641387 | 1.485337 | 0.19707 |
| C | -0.868634 | -0.375223 | -0.198796 | C | -0.810467 | 0.012081 | -0.241935 |
| C | -7.623943 | 0.273127 | -0.042201 | C | -7.76078 | 0.706145 | -0.0375 |
| C | -5.736786 | 1.398008 | -1.31265 | C | -6.79836 | -1.49108 | -0.864989 |
| C | 5.907561 | -1.116843 | -1.074495 | C | 5.791224 | -1.824708 | -0.845366 |
| C | 4.832829 | -2.38324 | 0.84698 | C | 4.499911 | -2.69228 | 1.162802 |
| C | -1.389133 | -2.843305 | -0.017725 | C | -1.718591 | -2.318288 | 0.12503 |
| C | -3.947286 | -1.059506 | -0.465796 | C | -3.955715 | -0.180675 | -0.489082 |
| C | -3.08591 | -1.127317 | 0.812644 | C | -3.120089 | -0.298794 | 0.802543 |
| C | 4.855976 | -1.066903 | 0.048514 | C | 4.737913 | -1.493231 | 0.226555 |
| C | -1.593549 | -1.461184 | 0.622662 | C | -1.698708 | -0.875206 | 0.654473 |
| O | 2.958915 | -1.708551 | -1.248077 | O | 2.788551 | -1.985772 | -1.058546 |
| O | 0.551875 | -0.741961 | -0.247603 | O | 0.537993 | -0.567823 | -0.225335 |
| O | 4.638666 | 1.799248 | -0.234083 | O | 4.960647 | 1.323072 | -0.380881 |
| O | 0.472844 | 3.858448 | 0.740299 | O | 1.146821 | 4.070119 | 0.290179 |
| O | -1.048054 | -1.467449 | 1.95189 | O | -1.158911 | -0.862381 | 1.985565 |
| H | 3.012659 | 3.793261 | 0.377276 | H | 3.650548 | 3.589774 | -0.016251 |
| H | -5.852024 | -1.604762 | 0.472366 | H | -5.248625 | 1.471361 | 0.153768 |
| H | -1.207331 | 1.049337 | 1.430782 | H | -0.926117 | 1.613462 | 1.24906 |
| H | -1.639067 | 1.676898 | -0.172219 | H | -1.26922 | 2.147828 | -0.408548 |
| H | -1.219308 | -0.38885 | -1.236461 | H | -1.148996 | -0.041666 | -1.282215 |
| H | -7.903907 | -0.599116 | 0.557121 | H | -7.517792 | 1.714185 | 0.31346 |
| H | -7.820187 | 1.175241 | 0.554348 | H | -8.417416 | 0.798234 | -0.914173 |
| H | -8.298735 | 0.323506 | -0.908413 | H | -8.354249 | 0.206525 | 0.74115 |
| H | -4.688219 | 1.350164 | -1.615526 | H | -7.37845 | -2.053563 | -0.120373 |
| H | -5.885162 | 2.335095 | -0.758309 | H | -5.894632 | -2.063224 | -1.087098 |
| H | -6.346647 | 1.479173 | -2.223137 | H | -7.411925 | -1.463771 | -1.77623 |

| | | | | | | | |
|---|---|---|---|---|---|---|---|
| H | 5.659701 | -1.905624 | -1.79335 | H | 5.441596 | -2.643034 | -1.484569 |
| H | 6.898742 | -1.330074 | -0.658254 | H | 6.729862 | -2.135267 | -0.372489 |
| H | 5.961071 | -0.163472 | -1.609947 | H | 5.999931 | -0.955816 | -1.477733 |
| H | 4.105709 | -2.343114 | 1.666519 | H | 5.43856 | -2.98215 | 1.648185 |
| H | 5.820193 | -2.576803 | 1.280978 | H | 4.1199 | -3.551359 | 0.601161 |
| H | 4.568259 | -3.223932 | 0.198018 | H | 3.774341 | -2.450351 | 1.948354 |
| H | -1.77143 | -2.879728 | -1.043138 | H | -2.116222 | -2.373367 | -0.8938 |
| H | -1.908067 | -3.604164 | 0.574264 | H | -2.34228 | -2.940048 | 0.775446 |
| H | -0.324534 | -3.094659 | -0.051255 | H | -0.706789 | -2.735474 | 0.110494 |
| H | -3.861732 | -2.01172 | -1.007747 | H | -4.068095 | -1.164513 | -0.953233 |
| H | -3.566378 | -0.286004 | -1.140617 | H | -3.418053 | 0.444885 | -1.217088 |
| H | -3.495565 | -1.89286 | 1.484176 | H | -3.650907 | -0.942149 | 1.514964 |
| H | -3.165739 | -0.175849 | 1.351629 | H | -3.046027 | 0.686697 | 1.279225 |
| H | 5.10508 | -0.247654 | 0.725593 | H | 5.095683 | -0.649283 | 0.819555 |
| H | 4.914686 | 2.716526 | -0.072277 | H | 5.369816 | 2.201108 | -0.307237 |
| H | -0.477718 | 3.669253 | 0.802739 | H | 0.177684 | 4.033247 | 0.342486 |
| H | -0.093123 | -1.626166 | 1.85277 | H | -0.243236 | -1.182863 | 1.910087 |
| **3b** (7) | | | | **3b** (8) | | | |
| C | -4.468556 | -1.11221 | -0.065149 | C | 3.230456 | 2.669104 | 0.412066 |
| C | -2.777415 | 0.677788 | -0.218481 | C | 3.001927 | 0.233034 | 0.083002 |
| C | -2.142958 | -1.715079 | -0.287647 | C | 1.114227 | 1.782733 | -0.327981 |
| C | -1.827931 | -0.360711 | -0.300513 | C | 1.668418 | 0.508481 | -0.280958 |
| C | -4.129007 | 0.242437 | -0.108476 | C | 3.772764 | 1.38161 | 0.426933 |
| C | -3.473991 | -2.096662 | -0.161697 | C | 1.895683 | 2.874113 | 0.033474 |
| C | 4.685249 | 0.636524 | 0.35292 | C | -4.767623 | -0.627271 | 0.327531 |
| C | -2.421632 | 2.121771 | -0.285866 | C | 3.595408 | -1.13211 | 0.069842 |
| C | 5.878259 | 0.525016 | -0.253482 | C | -5.843956 | -0.035253 | 0.871876 |
| C | -0.888353 | -2.531641 | -0.435759 | C | -0.329425 | 1.685719 | -0.741201 |
| C | 0.192493 | -1.43046 | -0.598853 | C | -0.415001 | 0.22012 | -1.225982 |
| C | 6.669915 | 1.756781 | -0.62841 | C | -6.1739 | -0.236167 | 2.333638 |
| C | 6.548107 | -0.778583 | -0.616423 | C | -6.814067 | 0.859953 | 0.138045 |
| C | -1.519012 | 3.645781 | 1.472892 | C | 3.403042 | -3.059929 | 1.649485 |
| C | -0.195446 | 3.241889 | -0.659348 | C | 2.586973 | -3.288861 | -0.745935 |
| C | 0.91309 | -1.355956 | 1.851406 | C | -1.563725 | -2.026348 | -1.283228 |
| C | 3.744152 | -0.455648 | 0.792246 | C | -4.286087 | -0.549668 | -1.098784 |
| C | 2.413753 | -0.406693 | 0.012122 | C | -2.916139 | 0.144281 | -1.296095 |
| C | -1.134764 | 2.621417 | 0.388547 | C | 2.736293 | -2.344867 | 0.460052 |
| C | 1.376395 | -1.488616 | 0.39525 | C | -1.658013 | -0.579584 | -0.772935 |
| O | -3.15898 | 2.936441 | -0.834409 | O | 4.776688 | -1.311463 | -0.214455 |
| O | -0.49125 | -0.143323 | -0.457348 | O | 0.777268 | -0.448213 | -0.677921 |
| O | -5.097756 | 1.19112 | 0.004066 | O | 5.061742 | 1.192094 | 0.817938 |
| O | -3.752218 | -3.427282 | -0.140704 | O | 1.32149 | 4.105452 | 0.000582 |
| O | 1.91718 | -2.801279 | 0.15431 | O | -1.708403 | -0.56912 | 0.660934 |

| | | | | | | | |
|---|---|---|---|---|---|---|---|
| H | -5.511005 | -1.402578 | 0.043749 | H | 3.849281 | 3.516533 | 0.69832 |
| H | 4.318851 | 1.64623 | 0.550635 | H | -4.146696 | -1.234845 | 0.983536 |
| H | -0.691124 | -3.17208 | 0.430131 | H | -1.00004 | 1.856416 | 0.109966 |
| H | -0.913546 | -3.187701 | -1.312314 | H | -0.604871 | 2.387387 | -1.533868 |
| H | 0.604703 | -1.44466 | -1.612724 | H | -0.317612 | 0.164658 | -2.316336 |
| H | 6.859266 | 1.789979 | -1.710679 | H | -7.174242 | -0.675208 | 2.457434 |
| H | 6.151842 | 2.6786 | -0.345051 | H | -5.451119 | -0.892012 | 2.829647 |
| H | 7.656062 | 1.755777 | -0.142839 | H | -6.190024 | 0.722653 | 2.871318 |
| H | 5.961004 | -1.65938 | -0.346517 | H | -6.859916 | 1.849526 | 0.613984 |
| H | 7.526329 | -0.86093 | -0.122512 | H | -7.832291 | 0.448879 | 0.185903 |
| H | 6.744242 | -0.822665 | -1.696728 | H | -6.561828 | 1.007835 | -0.914552 |
| H | -2.058071 | 4.489724 | 1.03052 | H | 3.480963 | -2.400576 | 2.52191 |
| H | -0.618978 | 4.028661 | 1.96691 | H | 4.411406 | -3.391179 | 1.382024 |
| H | -2.160098 | 3.196507 | 2.240504 | H | 2.814517 | -3.937161 | 1.940689 |
| H | 0.11956 | 2.49752 | -1.398292 | H | 2.067492 | -2.795578 | -1.574172 |
| H | 0.702632 | 3.643451 | -0.17633 | H | 2.012248 | -4.178725 | -0.465033 |
| H | -0.69823 | 4.060332 | -1.185981 | H | 3.571927 | -3.612766 | -1.099614 |
| H | 0.503635 | -0.360055 | 2.045354 | H | -1.602333 | -2.065701 | -2.377912 |
| H | 1.753548 | -1.509764 | 2.536974 | H | -2.38882 | -2.623349 | -0.886252 |
| H | 0.149632 | -2.100473 | 2.095923 | H | -0.625111 | -2.487083 | -0.960786 |
| H | 4.203674 | -1.441976 | 0.668017 | H | -4.237891 | -1.562963 | -1.521186 |
| H | 3.544738 | -0.33386 | 1.866311 | H | -5.007958 | -0.007493 | -1.718143 |
| H | 2.637787 | -0.520265 | -1.056489 | H | -2.960152 | 1.137935 | -0.836112 |
| H | 1.953934 | 0.581461 | 0.135491 | H | -2.774063 | 0.300362 | -2.37388 |
| H | -0.624285 | 1.781937 | 0.865502 | H | 1.744263 | -2.006599 | 0.767225 |
| H | -5.950897 | 0.750251 | 0.151229 | H | 5.432538 | 2.046377 | 1.095096 |
| H | -4.71121 | -3.561118 | -0.058926 | H | 1.972611 | 4.778052 | 0.261017 |
| H | 2.556118 | -2.99121 | 0.859133 | H | -0.876095 | -0.969912 | 0.96609 |
| **3b** (9) | | | | **3b** (10) | | | |
| C | 3.734177 | 2.14284 | 0.312069 | C | 3.604425 | -1.646069 | -0.783532 |
| C | 2.800646 | -0.085186 | -0.198532 | C | 2.193872 | 0.248875 | -0.067469 |
| C | 1.374808 | 1.938985 | -0.148062 | C | 1.394082 | -2.089785 | 0.076333 |
| C | 1.561995 | 0.575189 | -0.348911 | C | 1.210272 | -0.719026 | 0.233445 |
| C | 3.889756 | 0.767542 | 0.126915 | C | 3.401964 | -0.276863 | -0.599026 |
| C | 2.46558 | 2.730544 | 0.194358 | C | 2.595554 | -2.559413 | -0.442267 |
| C | -5.333926 | 0.19676 | -0.348958 | C | -3.479533 | 0.82752 | -1.345605 |
| C | 2.907994 | -1.559023 | -0.384749 | C | 1.925221 | 1.694938 | 0.158541 |
| C | -6.074084 | -0.815488 | 0.132091 | C | -4.253884 | 1.726417 | -0.714123 |
| C | -0.091313 | 2.261989 | -0.258902 | C | 0.147691 | -2.818493 | 0.50344 |
| C | -0.657774 | 0.942165 | -0.834298 | C | -0.693773 | -1.666116 | 1.111096 |
| C | -6.993498 | -1.599608 | -0.775739 | C | -3.998142 | 3.208108 | -0.868164 |
| C | -6.085437 | -1.266846 | 1.572843 | C | -5.423128 | 1.383352 | 0.176307 |
| C | 4.955734 | -3.023276 | -0.429523 | C | 2.640192 | 3.306598 | 1.93248 |

| | | | | | | | |
|---|---|---|---|---|---|---|---|
| C | 3.082768 | -3.460788 | 1.229025 | C | 3.311223 | 3.695246 | -0.48628 |
| C | -2.299544 | -0.966782 | -0.795353 | C | -2.964052 | -2.730751 | 1.187675 |
| C | -4.400916 | 1.120078 | 0.394216 | C | -3.578095 | -0.678888 | -1.356887 |
| C | -3.070494 | 1.436377 | -0.327668 | C | -2.28759 | -1.381244 | -0.869491 |
| C | 3.881549 | -2.385541 | 0.469714 | C | 3.049489 | 2.642127 | 0.605279 |
| C | -1.944266 | 0.394124 | -0.175905 | C | -2.166596 | -1.530543 | 0.669975 |
| O | 2.173842 | -2.143874 | -1.178333 | O | 0.783698 | 2.138572 | 0.050322 |
| O | 0.397494 | -0.063139 | -0.642875 | O | -0.005401 | -0.418763 | 0.757987 |
| O | 5.129737 | 0.204371 | 0.201338 | O | 4.363561 | 0.613449 | -0.977057 |
| O | 2.248512 | 4.057063 | 0.396199 | O | 2.735412 | -3.9019 | -0.602634 |
| O | -1.712068 | 0.244229 | 1.232986 | O | -2.708575 | -0.391632 | 1.355263 |
| H | 4.601747 | 2.758906 | 0.53799 | H | 4.539252 | -2.002026 | -1.21052 |
| H | -5.433183 | 0.423027 | -1.413342 | H | -2.664422 | 1.218541 | -1.959364 |
| H | -0.524193 | 2.47793 | 0.725598 | H | -0.353337 | -3.300178 | -0.345599 |
| H | -0.304745 | 3.111211 | -0.914607 | H | 0.340754 | -3.599802 | 1.245386 |
| H | -0.807252 | 1.023507 | -1.91739 | H | -0.680315 | -1.711215 | 2.205553 |
| H | -8.035525 | -1.54091 | -0.430424 | H | -3.141513 | 3.410998 | -1.518821 |
| H | -6.956885 | -1.236731 | -1.808054 | H | -4.875955 | 3.717289 | -1.290634 |
| H | -6.729831 | -2.666807 | -0.779229 | H | -3.807529 | 3.679416 | 0.106158 |
| H | -5.377452 | -0.722375 | 2.201882 | H | -5.55049 | 0.309792 | 0.325787 |
| H | -5.850252 | -2.337817 | 1.64516 | H | -6.356909 | 1.785218 | -0.241336 |
| H | -7.086544 | -1.141552 | 2.009124 | H | -5.300863 | 1.847932 | 1.164173 |
| H | 5.628718 | -3.649288 | 0.167508 | H | 2.481716 | 2.562572 | 2.721983 |
| H | 5.557608 | -2.258765 | -0.931334 | H | 1.71319 | 3.875337 | 1.81012 |
| H | 4.488341 | -3.653629 | -1.194091 | H | 3.426515 | 3.992196 | 2.26784 |
| H | 2.338005 | -3.011295 | 1.89633 | H | 4.081391 | 4.40167 | -0.156103 |
| H | 3.758742 | -4.070508 | 1.838966 | H | 2.396397 | 4.259255 | -0.699414 |
| H | 2.559112 | -4.118645 | 0.528386 | H | 3.654258 | 3.227292 | -1.414651 |
| H | -2.514611 | -0.868705 | -1.865692 | H | -2.575659 | -3.672037 | 0.786153 |
| H | -3.181155 | -1.386853 | -0.307669 | H | -4.014372 | -2.63963 | 0.893588 |
| H | -1.464491 | -1.665474 | -0.681871 | H | -2.921075 | -2.772932 | 2.281412 |
| H | -4.916423 | 2.083402 | 0.53092 | H | -4.441625 | -1.032743 | -0.786518 |
| H | -4.179843 | 0.751594 | 1.399729 | H | -3.747694 | -0.996424 | -2.396148 |
| H | -2.698288 | 2.387531 | 0.069043 | H | -1.424516 | -0.820099 | -1.248507 |
| H | -3.256749 | 1.595633 | -1.398113 | H | -2.230298 | -2.379238 | -1.319145 |
| H | 4.373298 | -1.733832 | 1.194237 | H | 3.964822 | 2.069281 | 0.76567 |
| H | 5.779782 | 0.886105 | 0.438425 | H | 5.135206 | 0.130852 | -1.316654 |
| H | 3.083422 | 4.493638 | 0.633713 | H | 3.613006 | -4.098213 | -0.970712 |
| H | -0.974029 | -0.382845 | 1.322514 | H | -2.384641 | 0.403138 | 0.898889 |
| **3b** (11) | | | | **3b** (12) | | | |
| C | 3.406353 | 2.339798 | 0.549706 | C | -3.829367 | -2.115886 | 0.437886 |
| C | 2.712667 | -0.003504 | 0.207833 | C | -2.941029 | 0.134598 | -0.049591 |
| C | 1.16484 | 1.881733 | -0.216606 | C | -1.518643 | -1.886405 | -0.216675 |

| | | | | | | | |
|---|---|---|---|---|---|---|---|
| C | 1.455904 | 0.522592 | -0.159563 | C | -1.714045 | -0.512244 | -0.303718 |
| C | 3.67838 | 0.970719 | 0.577269 | C | -4.007649 | -0.735225 | 0.318856 |
| C | 2.142572 | 2.800181 | 0.149078 | C | -2.58187 | -2.696436 | 0.165513 |
| C | -5.093006 | 0.330135 | 0.319998 | C | 4.802176 | 0.60518 | -0.571934 |
| C | 2.950471 | -1.472438 | 0.199164 | C | -3.140446 | 1.602941 | -0.189755 |
| C | -5.850797 | -0.671974 | 0.79366 | C | 5.713234 | 0.161398 | 0.315012 |
| C | -0.270607 | 2.064156 | -0.633281 | C | -0.100493 | -2.224654 | -0.587986 |
| C | -0.633037 | 0.638928 | -1.11198 | C | 0.46492 | -0.835785 | -0.986357 |
| C | -6.307921 | -0.677974 | 2.234525 | C | 6.881097 | -0.684172 | -0.138722 |
| C | -6.327004 | -1.85631 | -0.013589 | C | 5.698993 | 0.459122 | 1.795112 |
| C | 4.125257 | -2.984795 | -1.407552 | C | -2.514304 | 3.473262 | 1.341974 |
| C | 4.955082 | -2.779241 | 0.984949 | C | -1.5793 | 3.39685 | -1.01935 |
| C | -2.174513 | -1.353871 | -1.179226 | C | 1.557294 | -0.263264 | 1.249525 |
| C | -4.569327 | 0.5325 | -1.078475 | C | 3.595421 | 1.482023 | -0.337162 |
| C | -3.109589 | 1.028851 | -1.161731 | C | 2.277401 | 0.909305 | -0.905896 |
| C | 4.318519 | -2.041306 | -0.206091 | C | -2.010398 | 2.567867 | 0.202358 |
| C | -2.001292 | 0.082 | -0.659255 | C | 1.762443 | -0.401492 | -0.266032 |
| O | 2.033847 | -2.251311 | 0.452978 | O | -4.209769 | 2.06967 | -0.573893 |
| O | 0.402305 | -0.243219 | -0.554022 | O | -0.591593 | 0.142136 | -0.715019 |
| O | 4.890665 | 0.520873 | 1.011286 | O | -5.216108 | -0.178634 | 0.603215 |
| O | 1.82264 | 4.120563 | 0.106836 | O | -2.35869 | -4.034513 | 0.254967 |
| O | -2.039379 | 0.090146 | 0.77458 | O | 2.658183 | -1.488978 | -0.551539 |
| H | 4.169902 | 3.048953 | 0.861292 | H | -4.664931 | -2.742691 | 0.74141 |
| H | -4.830149 | 1.127983 | 1.0166 | H | 4.952286 | 0.326307 | -1.617395 |
| H | -0.895534 | 2.364441 | 0.216847 | H | 0.459596 | -2.672535 | 0.239541 |
| H | -0.405549 | 2.802591 | -1.428837 | H | -0.039494 | -2.926901 | -1.425957 |
| H | -0.546352 | 0.562867 | -2.202612 | H | 0.647355 | -0.792876 | -2.064938 |
| H | -5.948166 | 0.199513 | 2.78159 | H | 6.89593 | -1.649857 | 0.384816 |
| H | -7.405088 | -0.696467 | 2.30316 | H | 6.853447 | -0.87825 | -1.2155 |
| H | -5.951573 | -1.576268 | 2.758789 | H | 7.834283 | -0.190208 | 0.095665 |
| H | -7.425259 | -1.878051 | -0.05612 | H | 4.838125 | 1.051372 | 2.113109 |
| H | -6.01941 | -2.798345 | 0.461432 | H | 6.606706 | 1.008159 | 2.080878 |
| H | -5.956175 | -1.86039 | -1.041427 | H | 5.705791 | -0.47264 | 2.37639 |
| H | 3.705442 | -2.454697 | -2.270567 | H | -2.790101 | 2.888775 | 2.227626 |
| H | 3.448008 | -3.805057 | -1.149763 | H | -3.393547 | 4.04104 | 1.021133 |
| H | 5.088418 | -3.411358 | -1.709533 | H | -1.731167 | 4.181393 | 1.63501 |
| H | 5.911907 | -3.223828 | 0.688456 | H | -0.8085 | 4.122426 | -0.735597 |
| H | 4.296402 | -3.582468 | 1.333423 | H | -2.43496 | 3.944989 | -1.428274 |
| H | 5.141221 | -2.096695 | 1.820437 | H | -1.170096 | 2.756977 | -1.808531 |
| H | -2.223493 | -1.378984 | -2.274186 | H | 0.897488 | 0.576859 | 1.488159 |
| H | -3.093893 | -1.788535 | -0.779348 | H | 2.516166 | -0.094473 | 1.749155 |
| H | -1.332837 | -1.978026 | -0.863611 | H | 1.125975 | -1.17516 | 1.673507 |
| H | -4.69163 | -0.369199 | -1.686071 | H | 3.475016 | 1.724534 | 0.7229 |

| | | | | | | | |
|---|---|---|---|---|---|---|---|
| H | -5.178789 | 1.305598 | -1.572837 | H | 3.76582 | 2.442922 | -0.845444 |
| H | -3.023213 | 1.971598 | -0.6079 | H | 2.407183 | 0.723251 | -1.980718 |
| H | -2.898067 | 1.263958 | -2.213203 | H | 1.496573 | 1.672462 | -0.809143 |
| H | 4.977689 | -1.223185 | -0.503059 | H | -1.151689 | 1.997561 | 0.562121 |
| H | 5.462149 | 1.279033 | 1.216691 | H | -5.815761 | -0.876807 | 0.914503 |
| H | 2.592571 | 4.652207 | 0.368999 | H | -3.175453 | -4.485998 | 0.525629 |
| H | -1.286872 | -0.454682 | 1.065095 | H | 3.549163 | -1.199958 | -0.280248 |
| **3b** (13) | | | | **3b** (14) | | | |
| C | -3.108745 | -1.541997 | 1.534467 | C | 3.264186 | 2.58646 | 0.201608 |
| C | -2.11327 | 0.194079 | 0.0904 | C | 2.937917 | 0.184227 | -0.260191 |
| C | -1.235104 | -2.120932 | 0.12801 | C | 1.038744 | 1.770779 | -0.252823 |
| C | -1.206605 | -0.805599 | -0.322106 | C | 1.569916 | 0.496065 | -0.423339 |
| C | -3.067183 | -0.231397 | 1.052925 | C | 3.76956 | 1.294697 | 0.044612 |
| C | -2.191456 | -2.494836 | 1.066383 | C | 1.886881 | 2.823025 | 0.070997 |
| C | 4.016341 | 0.783674 | 0.273288 | C | -5.529373 | 0.242107 | 0.507255 |
| C | -2.008182 | 1.577321 | -0.449523 | C | 3.423247 | -1.214771 | -0.40851 |
| C | 4.24318 | 1.871545 | 1.028853 | C | -6.696374 | -0.216346 | 0.026174 |
| C | -0.154462 | -2.916417 | -0.555684 | C | -0.457159 | 1.716672 | -0.399271 |
| C | 0.533029 | -1.835214 | -1.431877 | C | -0.687049 | 0.230486 | -0.791136 |
| C | 4.602824 | 3.191474 | 0.385224 | C | -7.889173 | 0.702339 | -0.106429 |
| C | 4.172495 | 1.91981 | 2.536717 | C | -6.953339 | -1.636446 | -0.417869 |
| C | -3.316775 | 2.827028 | -2.17483 | C | 5.769042 | -2.134069 | -0.370256 |
| C | -3.24997 | 3.661513 | 0.22351 | C | 4.021431 | -2.957706 | 1.278988 |
| C | 2.893564 | -2.674039 | -1.565255 | C | -1.647227 | -2.037913 | -0.235695 |
| C | 3.65005 | -0.605985 | 0.7311 | C | -4.235386 | -0.503297 | 0.708457 |
| C | 2.229225 | -1.077271 | 0.328189 | C | -3.109012 | 0.058872 | -0.18301 |
| C | -3.267652 | 2.422691 | -0.689764 | C | 4.551963 | -1.747316 | 0.488177 |
| C | 2.01713 | -1.492174 | -1.146351 | C | -1.702304 | -0.535998 | 0.079632 |
| O | -0.913293 | 2.038302 | -0.76706 | O | 2.883132 | -1.984105 | -1.200733 |
| O | -0.238067 | -0.59958 | -1.251587 | O | 0.619477 | -0.425994 | -0.709091 |
| O | -3.927717 | 0.705839 | 1.5434 | O | 5.112837 | 1.068325 | 0.12856 |
| O | -2.187396 | -3.784451 | 1.494631 | O | 1.334441 | 4.054043 | 0.241021 |
| O | 2.358592 | -0.416132 | -2.037793 | O | -1.299627 | -0.302797 | 1.439779 |
| H | -3.844457 | -1.81808 | 2.286331 | H | 3.941047 | 3.411082 | 0.412913 |
| H | 4.090993 | 0.899101 | -0.806324 | H | -5.484064 | 1.297476 | 0.784707 |
| H | 0.537623 | -3.382712 | 0.15431 | H | -0.96277 | 1.962821 | 0.540364 |
| H | -0.56176 | -3.723787 | -1.174751 | H | -0.837458 | 2.396246 | -1.169014 |
| H | 0.457242 | -2.072914 | -2.496609 | H | -0.998887 | 0.142526 | -1.837082 |
| H | 4.647529 | 3.115574 | -0.706034 | H | -8.234572 | 0.753089 | -1.148676 |
| H | 3.87104 | 3.970193 | 0.643593 | H | -8.740221 | 0.332188 | 0.482634 |
| H | 5.577268 | 3.554663 | 0.742236 | H | -7.661639 | 1.719949 | 0.226957 |
| H | 3.904181 | 0.964009 | 2.992791 | H | -7.762696 | -2.086281 | 0.17401 |
| H | 3.434867 | 2.666106 | 2.863523 | H | -6.07751 | -2.284027 | -0.334647 |

| | | | | | | | |
|---|---|---|---|---|---|---|---|
| H | 5.136659 | 2.235629 | 2.959506 | H | -7.289315 | -1.656834 | -1.463862 |
| H | -3.366624 | 1.948535 | -2.828868 | H | 5.4868 | -2.885201 | -1.116198 |
| H | -2.427913 | 3.404398 | -2.447591 | H | 6.560253 | -2.556176 | 0.259843 |
| H | -4.20305 | 3.441975 | -2.367591 | H | 6.177856 | -1.263127 | -0.892693 |
| H | -3.268741 | 3.375702 | 1.280228 | H | 3.689337 | -3.747552 | 0.59791 |
| H | -4.124472 | 4.29144 | 0.025061 | H | 3.174677 | -2.680105 | 1.917756 |
| H | -2.348713 | 4.256946 | 0.040852 | H | 4.810686 | -3.362666 | 1.922377 |
| H | 2.726047 | -3.54295 | -0.921061 | H | -2.334121 | -2.595839 | 0.410293 |
| H | 3.951563 | -2.404051 | -1.507777 | H | -0.636452 | -2.419154 | -0.075098 |
| H | 2.676721 | -2.959748 | -2.60012 | H | -1.934557 | -2.23353 | -1.27484 |
| H | 4.391138 | -1.320147 | 0.345635 | H | -3.931525 | -0.397144 | 1.760607 |
| H | 3.715225 | -0.675458 | 1.821725 | H | -4.365655 | -1.573915 | 0.525572 |
| H | 1.517257 | -0.278518 | 0.56961 | H | -3.060481 | 1.144546 | -0.031691 |
| H | 1.967845 | -1.931716 | 0.964691 | H | -3.372499 | -0.100719 | -1.236608 |
| H | -4.153108 | 1.828197 | -0.456911 | H | 4.852803 | -0.970396 | 1.193572 |
| H | -4.523221 | 0.291575 | 2.189351 | H | 5.567505 | 1.89786 | 0.349493 |
| H | -2.894544 | -3.916246 | 2.14801 | H | 2.025954 | 4.6928 | 0.481476 |
| H | 1.745858 | 0.310833 | -1.82859 | H | -1.763528 | -0.944889 | 1.9994 |
| **3b** (15) | | | | **3b** (16) | | | |
| C | -4.029083 | -1.144732 | -0.493821 | C | -4.044863 | -1.143645 | -0.379209 |
| C | -2.24088 | 0.542907 | -0.272121 | C | -2.265344 | 0.507922 | 0.06262 |
| C | -1.727462 | -1.869697 | -0.494097 | C | -1.738281 | -1.840861 | -0.517169 |
| C | -1.337955 | -0.538781 | -0.376981 | C | -1.357873 | -0.548287 | -0.169631 |
| C | -3.612417 | 0.180197 | -0.353162 | C | -3.635019 | 0.148884 | -0.046267 |
| C | -3.081886 | -2.177984 | -0.553687 | C | -3.091405 | -2.143759 | -0.622721 |
| C | 4.822711 | 0.053177 | 0.035374 | C | 4.825328 | 0.065197 | 0.028706 |
| C | -1.741391 | 1.933384 | -0.093138 | C | -1.776818 | 1.868569 | 0.418882 |
| C | 5.227447 | 1.164803 | -0.600344 | C | 5.195516 | 1.271321 | -0.431754 |
| C | -0.5107 | -2.75469 | -0.51763 | C | -0.513756 | -2.688455 | -0.734264 |
| C | 0.635461 | -1.72072 | -0.353024 | C | 0.627328 | -1.675353 | -0.447366 |
| C | 6.477701 | 1.892834 | -0.162489 | C | 6.460401 | 1.93183 | 0.066801 |
| C | 4.522692 | 1.795444 | -1.777436 | C | 4.434723 | 2.078945 | -1.455889 |
| C | -3.027606 | 4.097811 | -0.132337 | C | -1.523359 | 3.961563 | -0.924198 |
| C | -1.611937 | 3.466188 | 1.879274 | C | -3.066707 | 3.925158 | 1.092807 |
| C | 0.701324 | -1.823417 | 2.201088 | C | 0.83425 | -2.20874 | 2.043368 |
| C | 3.616046 | -0.806353 | -0.238909 | C | 3.612268 | -0.752571 | -0.330825 |
| C | 2.638967 | -0.816238 | 0.95406 | C | 2.691042 | -0.971925 | 0.886803 |
| C | -2.526753 | 2.94496 | 0.756225 | C | -2.508254 | 3.118656 | -0.092884 |
| C | 1.519793 | -1.884141 | 0.903853 | C | 1.581368 | -2.036085 | 0.713348 |
| O | -0.660161 | 2.273697 | -0.569211 | O | -0.746748 | 2.009444 | 1.074758 |
| O | 0.011537 | -0.398502 | -0.314429 | O | -0.008638 | -0.397102 | -0.134107 |
| O | -4.532837 | 1.186884 | -0.338979 | O | -4.561636 | 1.108655 | 0.239143 |
| O | -3.428999 | -3.486549 | -0.676418 | O | -3.43174 | -3.416446 | -0.958696 |

| | | | | | | | |
|---|---|---|---|---|---|---|---|
| O | 2.081773 | -3.197137 | 0.730731 | O | 2.144453 | -3.290307 | 0.290639 |
| H | -5.089669 | -1.37204 | -0.572053 | H | -5.105698 | -1.376539 | -0.431987 |
| H | 5.429955 | -0.286662 | 0.877598 | H | 5.471097 | -0.401131 | 0.776417 |
| H | -0.507311 | -3.500434 | 0.283317 | H | -0.467361 | -3.55653 | -0.069099 |
| H | -0.411422 | -3.304047 | -1.460627 | H | -0.44894 | -3.072929 | -1.758264 |
| H | 1.282993 | -1.7263 | -1.232525 | H | 1.224359 | -1.509738 | -1.347078 |
| H | 7.208587 | 1.951256 | -0.981553 | H | 7.15685 | 2.128579 | -0.760711 |
| H | 6.251108 | 2.930023 | 0.122647 | H | 6.242898 | 2.907245 | 0.524706 |
| H | 6.960896 | 1.403853 | 0.689632 | H | 6.979052 | 1.315698 | 0.808419 |
| H | 4.259001 | 2.838564 | -1.554252 | H | 4.159665 | 3.060238 | -1.044802 |
| H | 3.607789 | 1.273666 | -2.066985 | H | 3.519577 | 1.59227 | -1.800887 |
| H | 5.185166 | 1.826373 | -2.653881 | H | 5.062761 | 2.278719 | -2.335367 |
| H | -2.184962 | 4.588112 | -0.632348 | H | -0.666196 | 4.266243 | -0.315471 |
| H | -3.548983 | 4.846343 | 0.475206 | H | -2.022119 | 4.863212 | -1.297057 |
| H | -3.722109 | 3.736286 | -0.897485 | H | -1.146307 | 3.402168 | -1.788414 |
| H | -0.724851 | 3.95176 | 1.460912 | H | -2.258588 | 4.210814 | 1.775261 |
| H | -1.278106 | 2.653185 | 2.534814 | H | -3.805458 | 3.344372 | 1.654518 |
| H | -2.150296 | 4.196205 | 2.494142 | H | -3.553052 | 4.839619 | 0.734494 |
| H | 1.359991 | -1.98142 | 3.063348 | H | 1.539639 | -2.509093 | 2.827329 |
| H | -0.072318 | -2.596594 | 2.22564 | H | 0.062015 | -2.980814 | 1.975564 |
| H | 0.224956 | -0.84545 | 2.320136 | H | 0.366896 | -1.269867 | 2.355857 |
| H | 3.100072 | -0.475969 | -1.145573 | H | 3.054152 | -0.284383 | -1.147431 |
| H | 3.940845 | -1.840154 | -0.421729 | H | 3.934096 | -1.737971 | -0.695684 |
| H | 2.178661 | 0.172629 | 1.061855 | H | 2.226044 | -0.02222 | 1.175149 |
| H | 3.210433 | -0.990977 | 1.877407 | H | 3.307498 | -1.284866 | 1.742419 |
| H | -3.389638 | 2.450127 | 1.205837 | H | -3.339167 | 2.815285 | -0.732819 |
| H | -5.425924 | 0.807783 | -0.387357 | H | -5.453327 | 0.739421 | 0.128476 |
| H | -4.396899 | -3.568205 | -0.704366 | H | -4.399159 | -3.495972 | -1.004724 |
| H | 2.647105 | -3.374768 | 1.500512 | H | 2.749976 | -3.585965 | 0.990353 |
| **3b** (17) | | | | **3b** (18) | | | |
| C | 2.885016 | 2.727335 | 0.260349 | C | -4.025956 | -1.577587 | -0.33455 |
| C | 2.884079 | 0.299076 | -0.18648 | C | -2.504136 | 0.366642 | -0.306297 |
| C | 0.824189 | 1.657449 | -0.394482 | C | -1.642223 | -1.954432 | -0.354411 |
| C | 1.511203 | 0.450847 | -0.487036 | C | -1.452542 | -0.575689 | -0.357429 |
| C | 3.547463 | 1.501422 | 0.179149 | C | -3.807947 | -0.198728 | -0.314669 |
| C | 1.510727 | 2.803919 | -0.01285 | C | -2.936667 | -2.461785 | -0.341344 |
| C | -5.578859 | -0.726693 | -0.335172 | C | 4.450379 | 1.056224 | -0.503305 |
| C | 3.536934 | -1.035667 | -0.268381 | C | -2.214569 | 1.825542 | -0.252184 |
| C | -6.52441 | -0.04917 | 0.335941 | C | 5.792692 | 1.067037 | -0.45999 |
| C | -0.63323 | 1.432219 | -0.690273 | C | -0.308505 | -2.65142 | -0.345054 |
| C | -0.654035 | -0.074891 | -1.06801 | C | 0.67503 | -1.450276 | -0.346801 |
| C | -7.833731 | 0.308448 | -0.329221 | C | 6.553508 | 2.372921 | -0.43752 |
| C | -6.408047 | 0.413354 | 1.768457 | C | 6.667854 | -0.163119 | -0.433733 |

| | | | | | | | |
|---|---|---|---|---|---|---|---|
| C | 5.981314 | -1.606679 | -0.068738 | C | -3.800319 | 3.772474 | -0.422714 |
| C | 4.264849 | -2.708897 | 1.443867 | C | -2.281038 | 3.510413 | 1.596235 |
| C | -1.393546 | -2.432912 | -0.554484 | C | 0.932584 | -1.317673 | 2.192156 |
| C | -4.219198 | -1.169415 | 0.140649 | C | 3.517762 | -0.127061 | -0.52359 |
| C | -3.087571 | -0.522223 | -0.684436 | C | 2.605127 | -0.153993 | 0.719195 |
| C | 4.663481 | -1.418887 | 0.704404 | C | -3.12617 | 2.777278 | 0.538427 |
| C | -1.65272 | -0.944385 | -0.278829 | C | 1.659646 | -1.373127 | 0.841223 |
| O | 3.142934 | -1.875355 | -1.075779 | O | -1.205493 | 2.281614 | -0.786152 |
| O | 0.704972 | -0.575258 | -0.849056 | O | -0.1379 | -0.234588 | -0.354655 |
| O | 4.891899 | 1.437649 | 0.403302 | O | -4.866293 | 0.660909 | -0.354434 |
| O | 0.809096 | 3.96573 | 0.071568 | O | -3.086839 | -3.812727 | -0.343821 |
| O | -1.409106 | -0.649588 | 1.107066 | O | 2.399234 | -2.601905 | 0.724577 |
| H | 3.440225 | 3.624615 | 0.523282 | H | -5.042168 | -1.964003 | -0.361486 |
| H | -5.798308 | -0.99621 | -1.370579 | H | 3.944755 | 2.024451 | -0.514943 |
| H | -1.258799 | 1.630536 | 0.1863 | H | -0.169662 | -3.294754 | 0.529548 |
| H | -1.004678 | 2.054969 | -1.510782 | H | -0.158083 | -3.282592 | -1.227773 |
| H | -0.852415 | -0.211585 | -2.136147 | H | 1.251431 | -1.436804 | -1.27467 |
| H | -8.684855 | -0.123104 | 0.216335 | H | 7.181607 | 2.44907 | 0.461435 |
| H | -7.99021 | 1.396437 | -0.333618 | H | 7.235067 | 2.448426 | -1.296701 |
| H | -7.877743 | -0.046095 | -1.364029 | H | 5.882855 | 3.23797 | -0.458367 |
| H | -6.530134 | 1.50351 | 1.832271 | H | 7.350127 | -0.170693 | -1.295271 |
| H | -5.453602 | 0.154675 | 2.232422 | H | 6.102246 | -1.097558 | -0.445858 |
| H | -7.209535 | -0.02255 | 2.381032 | H | 7.304212 | -0.164693 | 0.462244 |
| H | 5.864877 | -2.374794 | -0.841489 | H | -3.044104 | 4.339962 | -0.976547 |
| H | 6.779443 | -1.923354 | 0.612467 | H | -4.420593 | 4.48175 | 0.137076 |
| H | 6.294629 | -0.674883 | -0.550209 | H | -4.441891 | 3.254882 | -1.1432 |
| H | 4.102081 | -3.526431 | 0.734756 | H | -1.478467 | 4.083642 | 1.121834 |
| H | 3.343775 | -2.571488 | 2.022435 | H | -1.825242 | 2.807206 | 2.303294 |
| H | 5.058902 | -3.004468 | 2.138806 | H | -2.910774 | 4.201978 | 2.167205 |
| H | -2.07697 | -3.061504 | 0.026745 | H | 1.665795 | -1.308631 | 3.007417 |
| H | -0.368775 | -2.693405 | -0.280994 | H | 0.283345 | -2.186326 | 2.336528 |
| H | -1.543249 | -2.669983 | -1.613799 | H | 0.32766 | -0.409646 | 2.276514 |
| H | -4.075817 | -0.928696 | 1.19894 | H | 2.897722 | -0.063483 | -1.429434 |
| H | -4.156965 | -2.263517 | 0.053577 | H | 4.062862 | -1.073123 | -0.58593 |
| H | -3.17738 | 0.566246 | -0.585254 | H | 2.000097 | 0.759947 | 0.757022 |
| H | -3.23438 | -0.754609 | -1.747821 | H | 3.239778 | -0.139537 | 1.616475 |
| H | 4.799012 | -0.620532 | 1.436583 | H | -3.901963 | 2.201057 | 1.045792 |
| H | 5.218308 | 2.318672 | 0.650061 | H | -5.695077 | 0.154304 | -0.344025 |
| H | 1.399998 | 4.685722 | 0.348345 | H | -4.031933 | -4.03784 | -0.327108 |
| H | -1.86925 | -1.319101 | 1.637008 | H | 3.041673 | -2.623246 | 1.452945 |
| **3b** (19) | | | | **3b** (20) | | | |
| C | 2.992191 | 2.8528 | 0.076718 | C | -4.351583 | -0.453098 | -0.364581 |
| C | 3.02723 | 0.38812 | 0.171685 | C | -2.35854 | 0.915472 | 0.129268 |

| | | | | | | | |
|---|---|---|---|---|---|---|---|
| C | 0.971497 | 1.632885 | -0.422694 | C | -2.167121 | -1.46478 | -0.528379 |
| C | 1.666918 | 0.451187 | -0.192443 | C | -1.611989 | -0.24562 | -0.155851 |
| C | 3.665871 | 1.652564 | 0.317633 | C | -3.770108 | 0.757487 | 0.021757 |
| C | 1.636717 | 2.845501 | -0.285138 | C | -3.549384 | -1.571381 | -0.635249 |
| C | -5.434936 | -0.757546 | -0.340406 | C | 4.61356 | -0.554408 | -0.081666 |
| C | 3.754561 | -0.889072 | 0.407196 | C | -1.742549 | 2.209829 | 0.530557 |
| C | -6.422783 | 0.071921 | 0.033283 | C | 5.125775 | 0.626434 | -0.465033 |
| C | -0.470126 | 1.321866 | -0.716957 | C | -1.078825 | -2.487382 | -0.711325 |
| C | -0.462603 | -0.230949 | -0.757736 | C | 0.196279 | -1.649934 | -0.42316 |
| C | -7.662635 | 0.225021 | -0.81755 | C | 6.506052 | 1.050435 | -0.0182 |
| C | -6.422169 | 0.911403 | 1.288197 | C | 4.423333 | 1.629629 | -1.348183 |
| C | 3.127584 | -3.334376 | 0.34342 | C | 0.640118 | 2.907285 | 0.974505 |
| C | 4.721282 | -2.341674 | -1.368231 | C | -0.684715 | 3.920609 | -0.942516 |
| C | -1.26935 | -2.432582 | 0.18299 | C | 0.411214 | -2.306933 | 2.036875 |
| C | -4.128127 | -1.040889 | 0.354746 | C | 3.269867 | -1.151909 | -0.409854 |
| C | -2.92191 | -0.60989 | -0.505165 | C | 2.393007 | -1.313752 | 0.848538 |
| C | 3.470936 | -2.096647 | -0.499622 | C | -0.417511 | 2.650689 | -0.111146 |
| C | -1.532881 | -0.921594 | 0.108874 | C | 1.127157 | -2.189157 | 0.684319 |
| O | 4.626527 | -0.982257 | 1.26688 | O | -2.297764 | 2.965313 | 1.323868 |
| O | 0.872506 | -0.646216 | -0.312549 | O | -0.254869 | -0.309619 | -0.042991 |
| O | 4.985178 | 1.666032 | 0.651676 | O | -4.553269 | 1.844039 | 0.259863 |
| O | 0.930782 | 3.986234 | -0.508366 | O | -4.065188 | -2.775251 | -0.999228 |
| O | -1.410675 | -0.335488 | 1.415413 | O | 1.481165 | -3.493623 | 0.191824 |
| H | 3.522195 | 3.79762 | 0.173693 | H | -5.432974 | -0.52714 | -0.455227 |
| H | -5.568521 | -1.302223 | -1.277561 | H | 5.232272 | -1.177665 | 0.568074 |
| H | -1.133013 | 1.694132 | 0.071338 | H | -1.177427 | -3.338276 | -0.029457 |
| H | -0.813555 | 1.743476 | -1.667153 | H | -1.053408 | -2.898947 | -1.72602 |
| H | -0.562949 | -0.597125 | -1.784788 | H | 0.779285 | -1.528808 | -1.339071 |
| H | -8.565707 | -0.046587 | -0.252812 | H | 7.162714 | 1.231489 | -0.881015 |
| H | -7.798494 | 1.269326 | -1.13252 | H | 6.467035 | 1.994981 | 0.542749 |
| H | -7.622609 | -0.398983 | -1.71617 | H | 6.980702 | 0.296227 | 0.617768 |
| H | -6.522104 | 1.97681 | 1.038654 | H | 4.335142 | 2.597671 | -0.835357 |
| H | -5.519151 | 0.79096 | 1.890784 | H | 3.420441 | 1.317455 | -1.648404 |
| H | -7.285413 | 0.660909 | 1.920347 | H | 5.005505 | 1.817431 | -2.261278 |
| H | 3.939264 | -3.557337 | 1.043822 | H | 0.284858 | 3.662369 | 1.683867 |
| H | 2.978198 | -4.207394 | -0.301838 | H | 1.571183 | 3.268053 | 0.522992 |
| H | 2.210191 | -3.179246 | 0.922315 | H | 0.866052 | 1.992048 | 1.532834 |
| H | 5.590877 | -2.555604 | -0.737836 | H | -1.069975 | 4.723352 | -0.305302 |
| H | 4.956271 | -1.470017 | -1.990286 | H | -1.416823 | 3.734221 | -1.737089 |
| H | 4.556651 | -3.196829 | -2.033302 | H | 0.242885 | 4.265819 | -1.412709 |
| H | -2.02467 | -2.929686 | 0.801378 | H | 1.093811 | -2.733158 | 2.78155 |
| H | -0.287772 | -2.622721 | 0.622766 | H | -0.463941 | -2.960201 | 1.973149 |
| H | -1.301147 | -2.889306 | -0.812511 | H | 0.090566 | -1.325312 | 2.398811 |

| | | | | | | | |
|---|---|---|---|---|---|---|---|
| H | -4.078828 | -0.532109 | 1.322794 | H | 2.74715 | -0.547621 | -1.157603 |
| H | -4.067645 | -2.119237 | 0.560241 | H | 3.414437 | -2.146174 | -0.854914 |
| H | -3.000834 | 0.470067 | -0.677667 | H | 2.09797 | -0.325995 | 1.221451 |
| H | -2.98564 | -1.09172 | -1.490049 | H | 2.997974 | -1.771426 | 1.644861 |
| H | 2.6297 | -1.868798 | -1.156847 | H | -0.055723 | 1.866059 | -0.778484 |
| H | 5.304397 | 2.583276 | 0.626804 | H | -5.475866 | 1.614458 | 0.060001 |
| H | 1.504781 | 4.758133 | -0.371001 | H | -5.035014 | -2.721872 | -1.027409 |
| H | -1.932619 | -0.874519 | 2.030189 | H | 2.066349 | -3.903002 | 0.850473 |
| **3b** (21) | | | | **3b** (22) | | | |
| C | -4.06917 | -1.599625 | 0.142965 | C | -3.43739 | 2.607187 | -0.06803 |
| C | -2.573182 | 0.359456 | 0.032545 | C | -3.095669 | 0.165767 | -0.131232 |
| C | -1.738373 | -1.932612 | -0.389575 | C | -1.227284 | 1.722442 | 0.326198 |
| C | -1.544953 | -0.560578 | -0.264885 | C | -1.744123 | 0.443621 | 0.151685 |
| C | -3.850472 | -0.224152 | 0.243807 | C | -3.929738 | 1.312264 | -0.250438 |
| C | -3.008805 | -2.458979 | -0.18285 | C | -2.078129 | 2.815666 | 0.211364 |
| C | 4.753958 | 1.027712 | 0.521473 | C | 5.419238 | 0.483432 | -0.259462 |
| C | -2.282391 | 1.815703 | 0.133703 | C | -3.626375 | -1.213779 | -0.320853 |
| C | 5.969802 | 1.116364 | -0.041807 | C | 6.573003 | -0.065801 | 0.153574 |
| C | -0.439477 | -2.593672 | -0.76394 | C | 0.258759 | 1.641313 | 0.543382 |
| C | 0.535534 | -1.386677 | -0.790591 | C | 0.49561 | 0.106018 | 0.58826 |
| C | 6.625956 | 2.461905 | -0.251161 | C | 7.719726 | 0.805629 | 0.612015 |
| C | 6.79625 | -0.05714 | -0.510518 | C | 6.859276 | -1.547333 | 0.206729 |
| C | -2.661804 | 3.700179 | -1.461319 | C | -2.832786 | -3.61218 | -0.153283 |
| C | -3.76076 | 3.731619 | 0.829613 | C | -4.28312 | -2.567319 | 1.6535 |
| C | 1.199001 | -1.51965 | 1.670193 | C | 1.564251 | -1.955297 | -0.409537 |
| C | 3.932523 | -0.203881 | 0.804186 | C | 4.168583 | -0.20303 | -0.744165 |
| C | 2.617905 | -0.209368 | -0.002837 | C | 2.960464 | 0.107182 | 0.163333 |
| C | -3.307513 | 2.852779 | -0.349148 | C | -3.157439 | -2.329119 | 0.624266 |
| C | 1.703715 | -1.437038 | 0.224056 | C | 1.597132 | -0.421333 | -0.351722 |
| O | -1.191717 | 2.205799 | 0.545723 | O | -4.486827 | -1.458465 | -1.161438 |
| O | -0.262526 | -0.192451 | -0.519516 | O | -0.785496 | -0.517141 | 0.239027 |
| O | -4.869383 | 0.607156 | 0.607188 | O | -5.253351 | 1.112243 | -0.498224 |
| O | -3.164806 | -3.804064 | -0.304234 | O | -1.54387 | 4.055545 | 0.37636 |
| O | 2.393067 | -2.646159 | -0.143499 | O | 1.295426 | 0.126519 | -1.64543 |
| H | -5.059623 | -2.00545 | 0.335447 | H | -4.11261 | 3.45641 | -0.145506 |
| H | 4.268488 | 1.962755 | 0.80931 | H | 5.350885 | 1.573291 | -0.240586 |
| H | -0.11925 | -3.357273 | -0.048332 | H | 0.814045 | 2.103723 | -0.279444 |
| H | -0.488622 | -3.081476 | -1.744046 | H | 0.581611 | 2.120824 | 1.473231 |
| H | 0.962738 | -1.250523 | -1.78859 | H | 0.723412 | -0.227553 | 1.605918 |
| H | 7.589231 | 2.51803 | 0.275348 | H | 8.003338 | 0.571925 | 1.647937 |
| H | 6.845508 | 2.632814 | -1.314602 | H | 8.616509 | 0.632575 | 0.000372 |
| H | 5.996161 | 3.28451 | 0.102576 | H | 7.471818 | 1.870589 | 0.55859 |
| H | 7.029701 | 0.039059 | -1.579982 | H | 7.721194 | -1.797013 | -0.42764 |

| | | | | | | | |
|---|---|---|---|---|---|---|---|
| H | 6.306573 | -1.021947 | -0.359866 | H | 6.017827 | -2.166038 | -0.113581 |
| H | 7.761648 | -0.083494 | 0.013923 | H | 7.130855 | -1.849813 | 1.227598 |
| H | -1.767469 | 4.208689 | -1.087776 | H | -3.700356 | -3.938235 | -0.735764 |
| H | -3.369286 | 4.457509 | -1.817378 | H | -2.557066 | -4.417155 | 0.537111 |
| H | -2.369447 | 3.082148 | -2.318451 | H | -1.996924 | -3.458328 | -0.845515 |
| H | -2.898873 | 4.228731 | 1.288089 | H | -5.201081 | -2.892221 | 1.151497 |
| H | -4.265106 | 3.135075 | 1.596846 | H | -4.50807 | -1.657749 | 2.222471 |
| H | -4.459004 | 4.502258 | 0.483803 | H | -3.984255 | -3.345557 | 2.364561 |
| H | 2.03772 | -1.636464 | 2.365004 | H | 2.3262 | -2.333708 | -1.099718 |
| H | 0.534345 | -2.377287 | 1.811198 | H | 0.586955 | -2.298418 | -0.756353 |
| H | 0.659527 | -0.608863 | 1.946739 | H | 1.760814 | -2.392649 | 0.575726 |
| H | 4.500023 | -1.113473 | 0.580492 | H | 3.942545 | 0.158827 | -1.758509 |
| H | 3.703324 | -0.231332 | 1.878945 | H | 4.318661 | -1.283589 | -0.821862 |
| H | 2.870288 | -0.171608 | -1.070639 | H | 2.890056 | 1.19594 | 0.279175 |
| H | 2.044574 | 0.698844 | 0.218995 | H | 3.146741 | -0.304854 | 1.1636 |
| H | -4.178648 | 2.338391 | -0.759355 | H | -2.265719 | -1.998094 | 1.159982 |
| H | -5.686128 | 0.091314 | 0.709229 | H | -5.710578 | 1.968634 | -0.462823 |
| H | -4.092705 | -4.042398 | -0.141702 | H | -2.235986 | 4.728001 | 0.262799 |
| H | 3.020774 | -2.854858 | 0.566028 | H | 1.83817 | -0.34136 | -2.299125 |
| | **3b** (23) | | | | | | |
| C | -4.348624 | -1.106434 | -0.171801 | | | | |
| C | -2.652823 | 0.666558 | 0.084182 | | | | |
| C | -2.018799 | -1.665617 | -0.457312 | | | | |
| C | -1.704046 | -0.336494 | -0.197441 | | | | |
| C | -4.004812 | 0.220092 | 0.101466 | | | | |
| C | -3.353572 | -2.055576 | -0.448132 | | | | |
| C | 4.352912 | 0.731738 | -0.232481 | | | | |
| C | -2.289803 | 2.082629 | 0.364951 | | | | |
| C | 5.688657 | 0.595836 | -0.265736 | | | | |
| C | -0.757266 | -2.453099 | -0.684978 | | | | |
| C | 0.337772 | -1.371542 | -0.47699 | | | | |
| C | 6.591449 | 1.774669 | 0.016906 | | | | |
| C | 6.420245 | -0.687425 | -0.578366 | | | | |
| C | -0.089775 | 3.305658 | 0.541111 | | | | |
| C | -1.750839 | 3.853157 | -1.302298 | | | | |
| C | 0.649728 | -1.814897 | 2.020305 | | | | |
| C | 3.293599 | -0.311653 | -0.476135 | | | | |
| C | 2.41351 | -0.532403 | 0.770968 | | | | |
| C | -1.146293 | 2.741363 | -0.422238 | | | | |
| C | 1.345513 | -1.646399 | 0.66206 | | | | |
| O | -2.923548 | 2.759089 | 1.170436 | | | | |
| O | -0.358799 | -0.115111 | -0.19292 | | | | |
| O | -4.977361 | 1.139263 | 0.346716 | | | | |

| | | | | | | | |
|---|---|---|---|---|---|---|---|
| O | -3.635394 | -3.359776 | -0.708348 | | | | |
| O | 1.952164 | -2.886121 | 0.255351 | | | | |
| H | -5.39521 | -1.402384 | -0.170909 | | | | |
| H | 3.958826 | 1.720818 | 0.01168 | | | | |
| H | -0.642497 | -3.290303 | 0.010256 | | | | |
| H | -0.70407 | -2.87345 | -1.695489 | | | | |
| H | 0.893663 | -1.210615 | -1.402911 | | | | |
| H | 7.252892 | 1.570048 | 0.870693 | | | | |
| H | 7.249054 | 1.983419 | -0.838937 | | | | |
| H | 6.021828 | 2.683217 | 0.23748 | | | | |
| H | 7.063196 | -0.561172 | -1.460691 | | | | |
| H | 5.752961 | -1.531186 | -0.76813 | | | | |
| H | 7.086588 | -0.963037 | 0.250832 | | | | |
| H | -0.547783 | 4.016236 | 1.237206 | | | | |
| H | 0.696938 | 3.826163 | -0.016447 | | | | |
| H | 0.380235 | 2.507351 | 1.126341 | | | | |
| H | -2.25043 | 4.604327 | -0.681742 | | | | |
| H | -2.485494 | 3.451236 | -2.009931 | | | | |
| H | -0.962031 | 4.348377 | -1.879527 | | | | |
| H | 1.391874 | -2.063777 | 2.787909 | | | | |
| H | -0.089887 | -2.620465 | 1.997417 | | | | |
| H | 0.150977 | -0.889719 | 2.32515 | | | | |
| H | 2.662859 | 0.023781 | -1.31217 | | | | |
| H | 3.727946 | -1.268552 | -0.779039 | | | | |
| H | 1.915093 | 0.405093 | 1.046245 | | | | |
| H | 3.067289 | -0.789474 | 1.616356 | | | | |
| H | -0.674755 | 2.000691 | -1.071022 | | | | |
| H | -5.84363 | 0.712704 | 0.239328 | | | | |
| H | -4.595646 | -3.502395 | -0.663996 | | | | |
| H | 2.602318 | -3.126646 | 0.935917 | | | | |

Key transitions, oscillator strengths, and rotatory strengths in the ECD of conformers **3b** (2) at B3LYP /6-311++G(d,p) level.

| Species | Excited State | *ΔE(eV)*[a] | *λ(nm)*[b] | *f*[c] | $R_{vel}$[d] |
|---|---|---|---|---|---|
| **3b** (2) | 94 -> 95 | 3.918700 | 316.39 | 0.0305 | -75.8784 |
| | 91 ->106 | 3.937700 | 314.86 | 0.0308 | 53.8871 |
| | 93 -> 95 | 4.528300 | 273.8 | 0.3085 | 40.2122 |
| | 94 -> 95 | 4.620700 | 268.32 | 0.0115 | -0.367 |
| | 94 ->104 | 5.112700 | 242.5 | 0.0023 | -1.5119 |
| | 92 -> 97 | 5.387300 | 230.14 | 0.0899 | 14.9736 |
| | 93 -> 96 | 5.478400 | 226.31 | 0.0012 | -1.3631 |
| | 93 -> 98 | 5.484200 | 226.08 | 0.005 | -9.8991 |
| | 92 -> 96 | 5.513300 | 224.88 | 0.0283 | 13.2656 |

| | | | | | |
|---|---|---|---|---|---|
| | 94 -> 98 | 5.520600 | 224.58 | 0.0927 | 1.4995 |
| | 94 -> 96 | 5.555800 | 223.16 | 0.0171 | 1.65 |
| | 94 -> 97 | 5.664500 | 218.88 | 0.023 | -0.0083 |
| | 93 -> 96 | 5.783600 | 214.37 | 0.0029 | -5.345 |
| | 93 -> 97 | 5.811700 | 213.33 | 0.0045 | -0.5347 |
| | 93 -> 99 | 5.867800 | 211.29 | 0.0002 | -0.7141 |
| | 92 -> 99 | 5.872700 | 211.12 | 0.0006 | -0.2906 |
| | 92 ->100 | 5.895400 | 210.31 | 0.0057 | 4.8729 |
| | 92 -> 98 | 5.906800 | 209.9 | 0.0048 | -2.7276 |
| | 91 -> 97 | 5.917100 | 209.54 | 0.0137 | -0.5534 |
| | 94 ->102 | 5.971800 | 207.62 | 0.0504 | -42.6022 |
| | 91 -> 99 | 5.972500 | 207.59 | 0.004 | -3.7499 |
| | 89 -> 95 | 5.981000 | 207.3 | 0.011 | -10.7148 |
| | 94 ->101 | 6.009900 | 206.3 | 0.0104 | -3.0651 |
| | 94 ->102 | 6.035900 | 205.41 | 0.0003 | -2.7403 |
| | 93 ->101 | 6.073300 | 204.15 | 0.0798 | -4.896 |
| | 94 ->103 | 6.089400 | 203.61 | 0.006 | 11.9507 |
| | 93 ->102 | 6.128200 | 202.32 | 0.0403 | 10.4256 |
| | 92 -> 97 | 6.162500 | 201.19 | 0.0472 | 9.4553 |
| | 92 ->100 | 6.200500 | 199.96 | 0.0721 | 3.0761 |
| | 93 ->101 | 6.238400 | 198.74 | 0.0833 | -20.8478 |
| | 92 ->106 | 6.258400 | 198.11 | 0.0245 | 6.1696 |
| | 92 ->102 | 6.321300 | 196.14 | 0.0684 | 31.3548 |
| | 94 ->106 | 6.349800 | 195.26 | 0.1312 | -41.1168 |
| | 91 ->100 | 6.367200 | 194.72 | 0.1481 | -2.2898 |
| | 92 ->101 | 6.393300 | 193.93 | 0.0191 | -7.9926 |
| | 91 -> 98 | 6.404700 | 193.58 | 0.0041 | 11.0496 |
| | 92 ->104 | 6.407900 | 193.49 | 0.0043 | -4.4652 |
| | 93 ->104 | 6.421300 | 193.08 | 0.0702 | 20.2539 |
| | 91 ->104 | 6.446100 | 192.34 | 0.0119 | 25.4312 |
| | 92 ->104 | 6.463700 | 191.82 | 0.0074 | -15.6493 |

[a]Excitation energy. [b]Wavelength. [c]Oscillator strength. [d]Rotatory strength in velocity form ($10^{-40}$cgs.).

HRESIMS of compound **1**

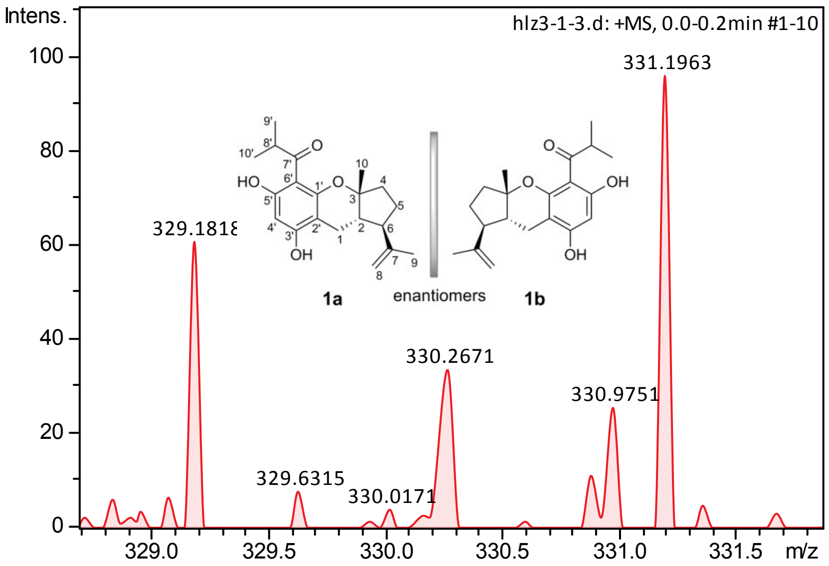

$^1$H NMR of compound **1** (in methanol-$d_4$)

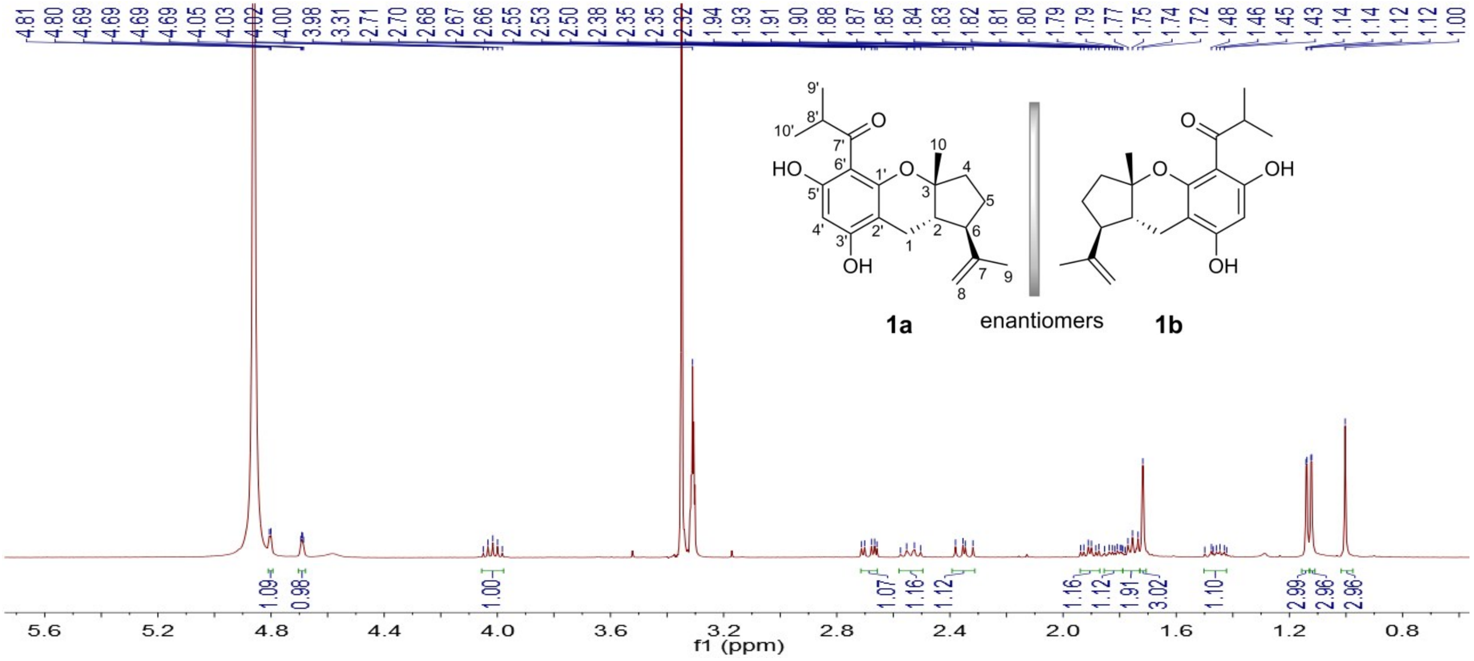

$^{13}C$ NMR of compound **1** (in methanol-$d_4$)

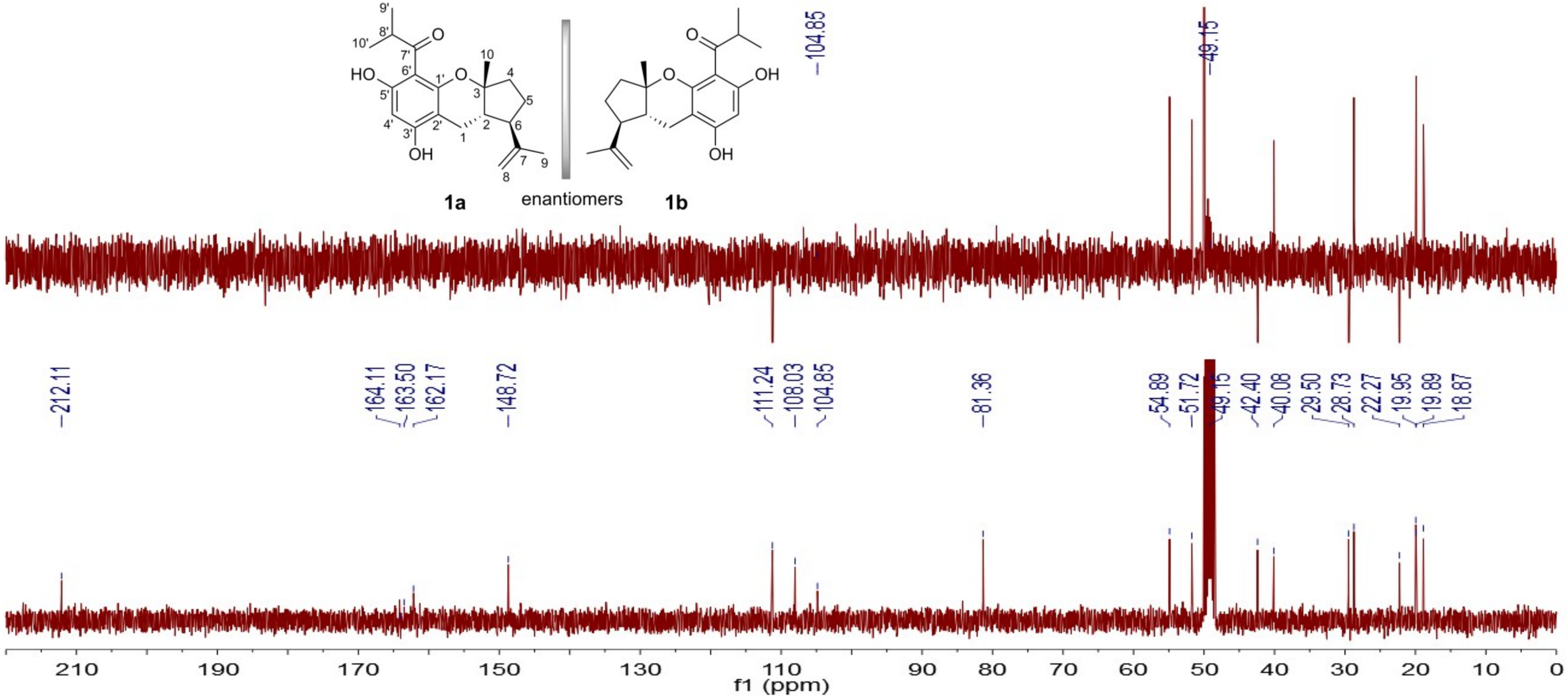

HSQC of compound **1** (in methanol-$d_4$)

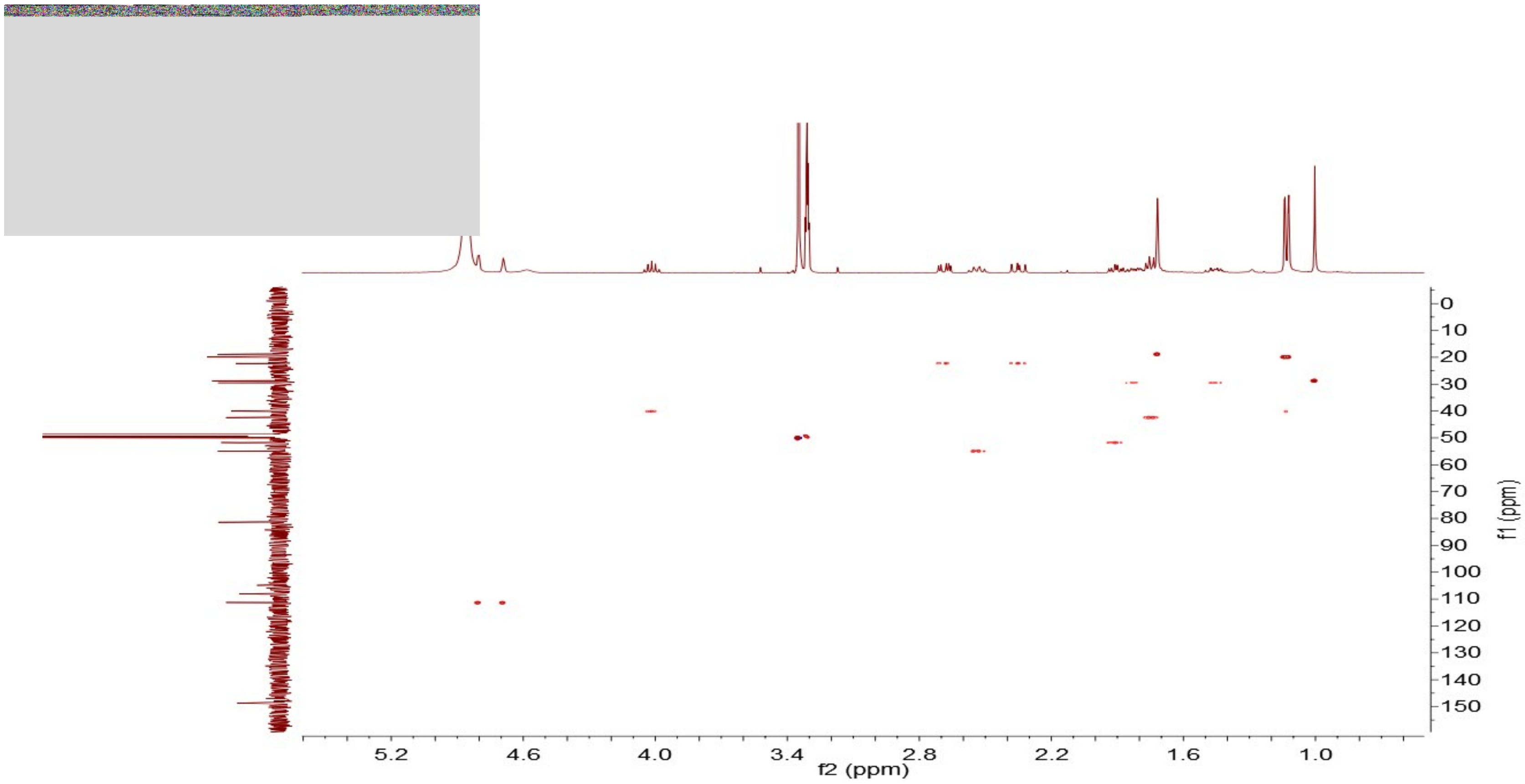

HMBC of compound **1** (in methanol-$d_4$)

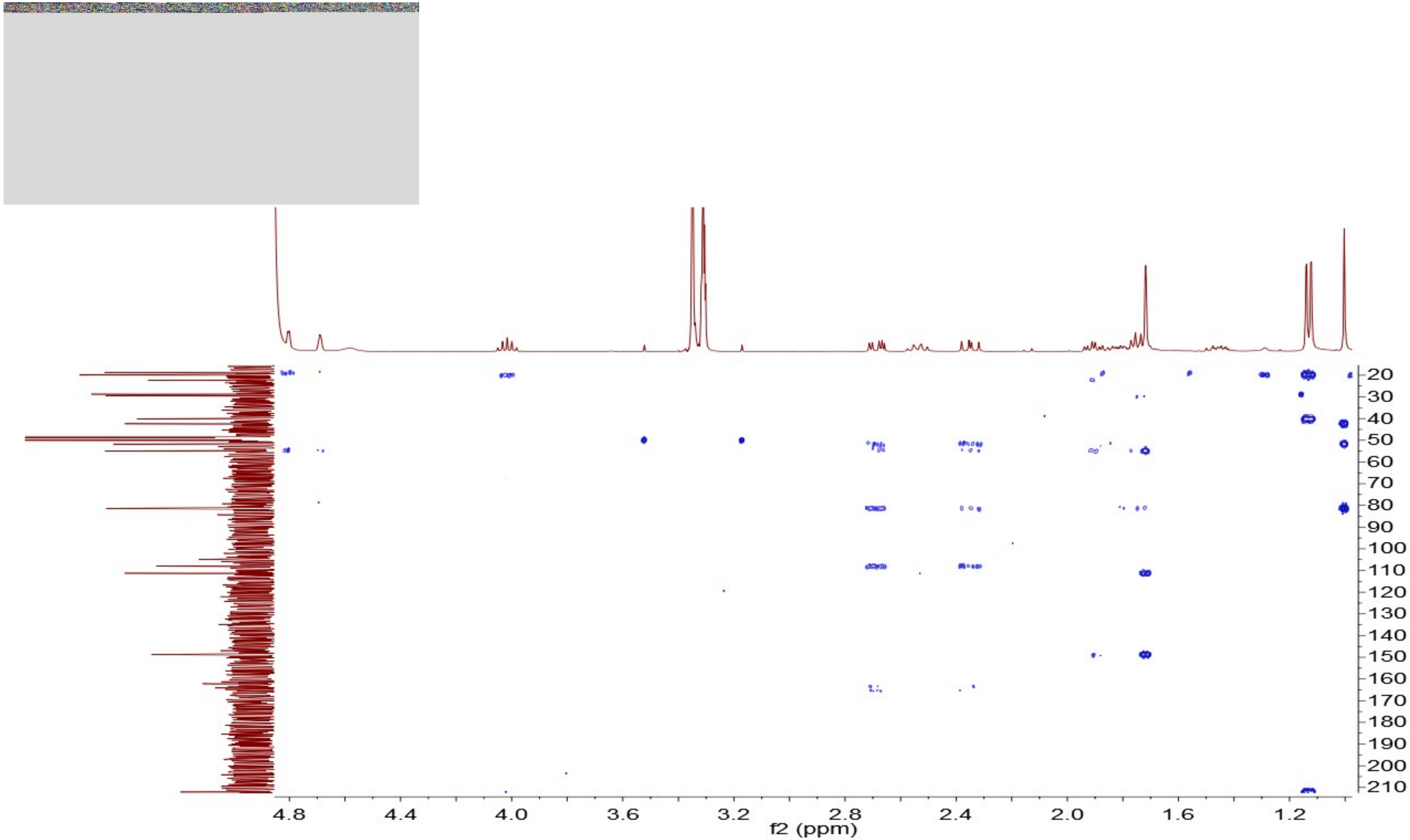

$^{1}H–^{1}H$ COSY of compound **1** (in methanol-$d_4$)

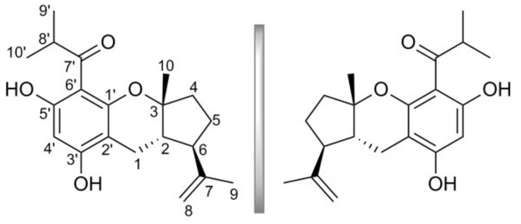

**1a** enantiomers **1b**

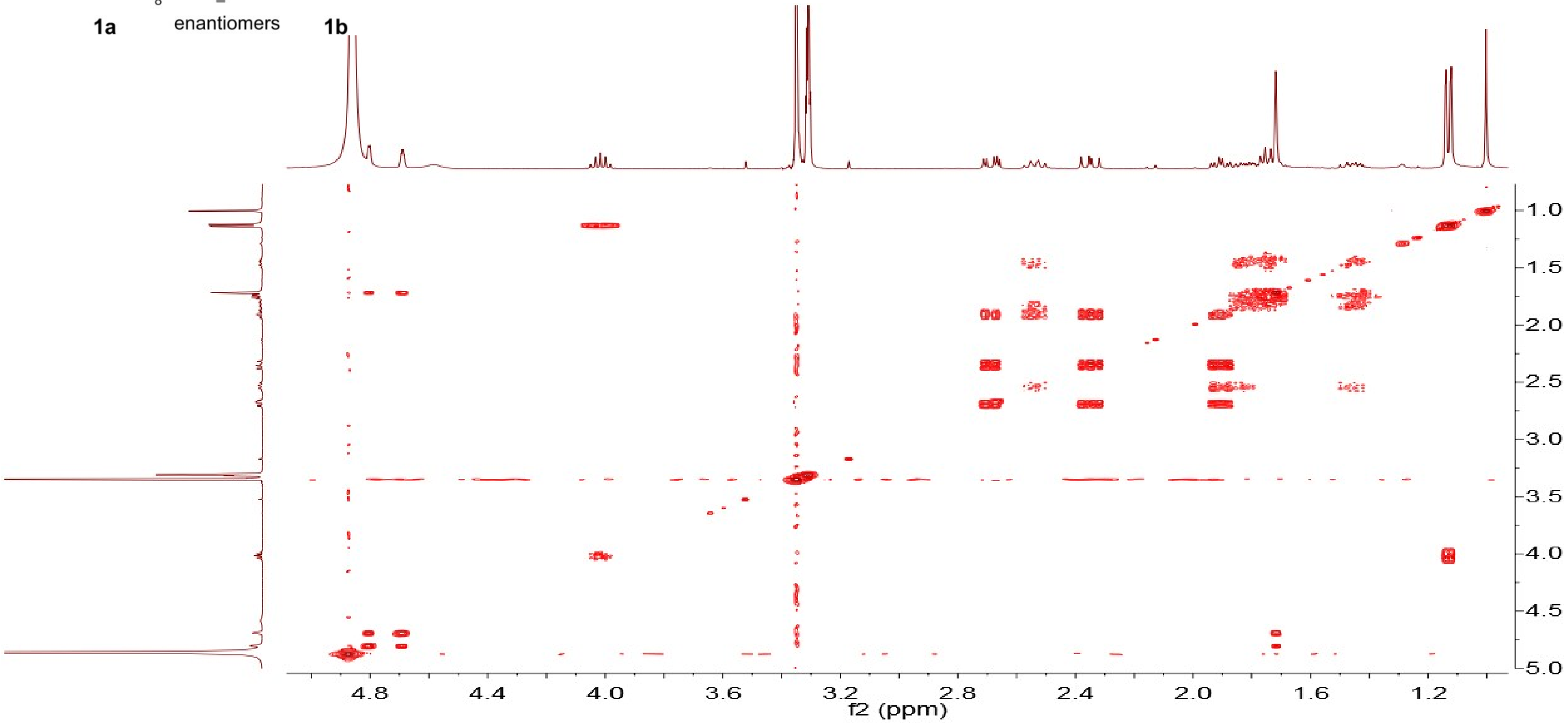

NOESY of compound **1** (in methanol-$d_4$)

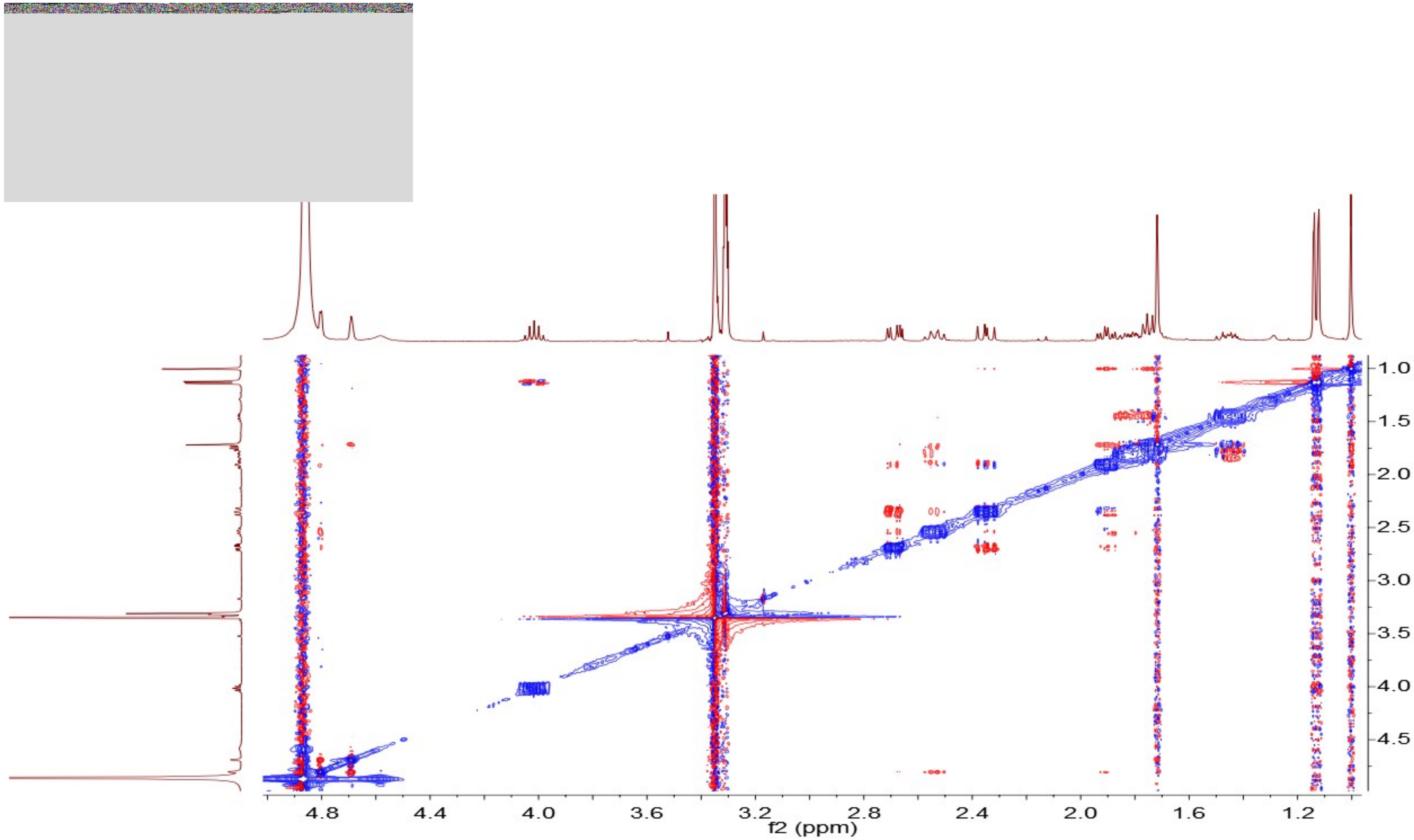

UV of compound **1** (in MeOH)

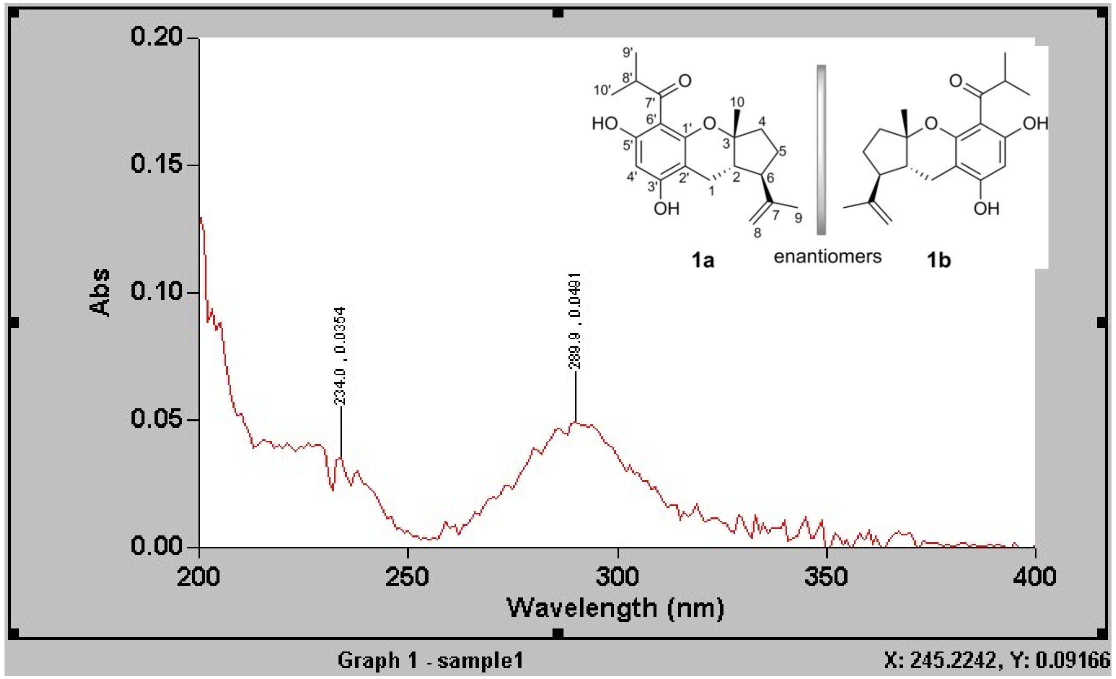

IR of compound **1** (KBr disc)

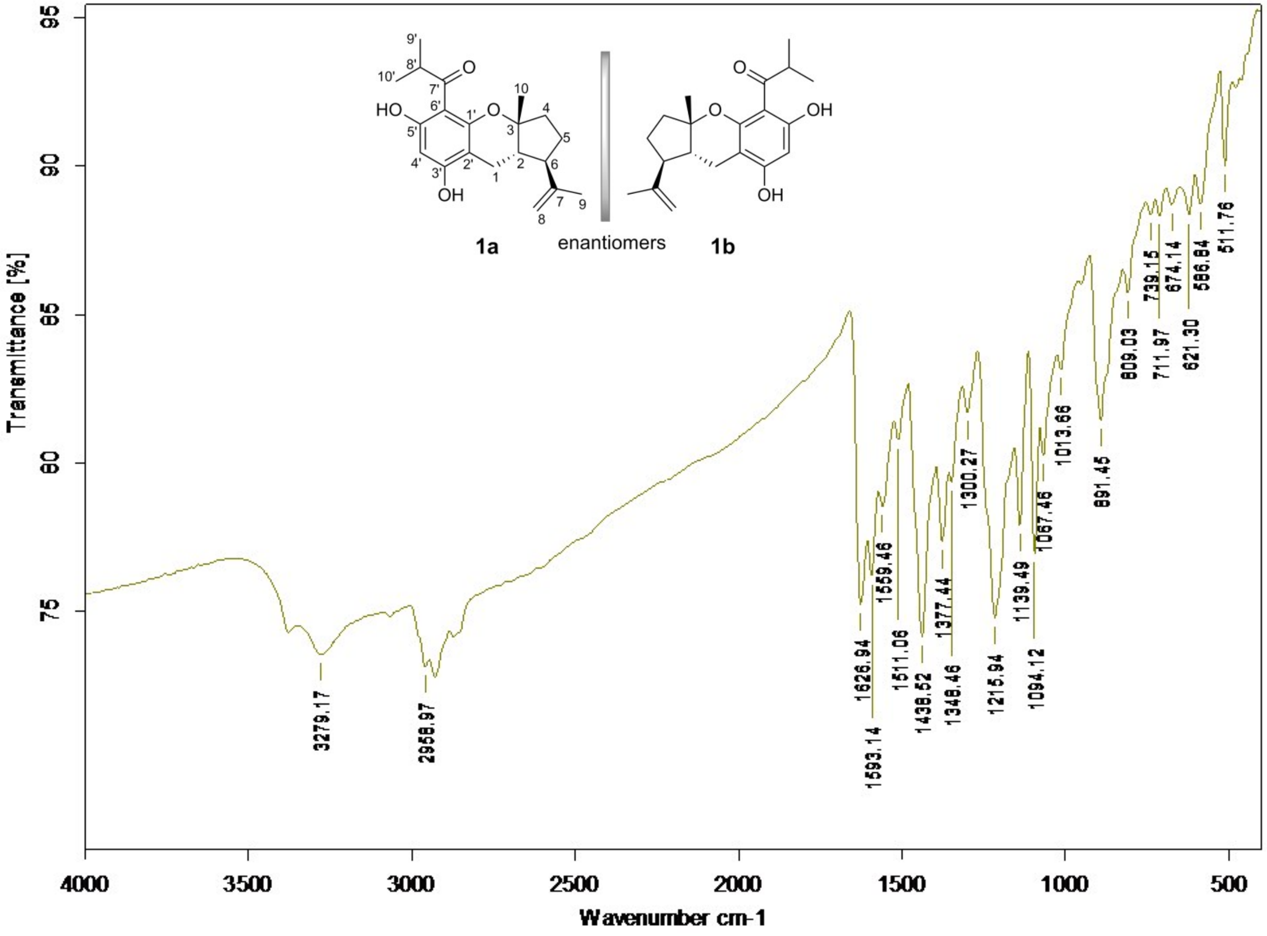

HRESIMS of compound **2**

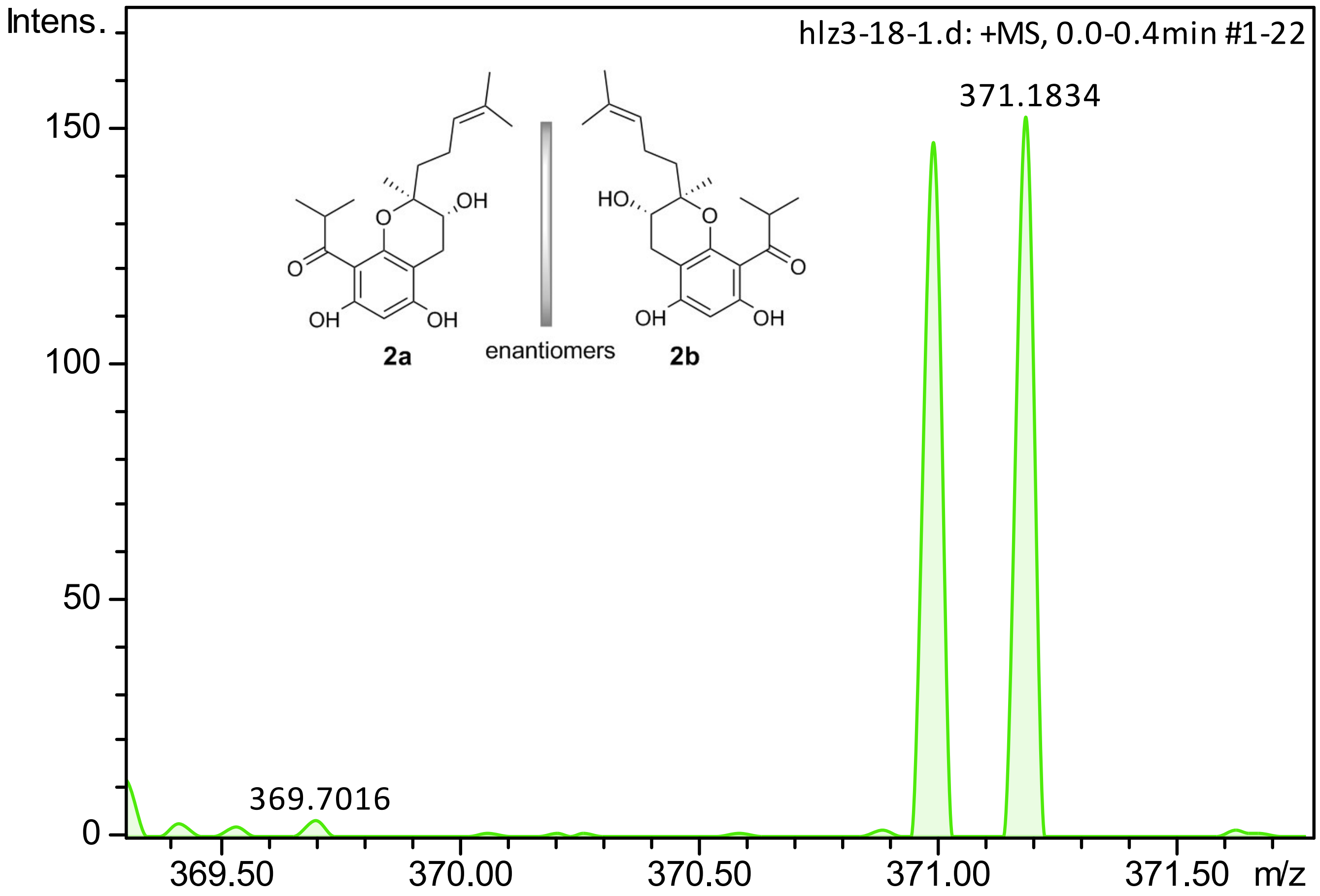

$^{1}$H NMR of compound **2** (in methanol-$d_4$)

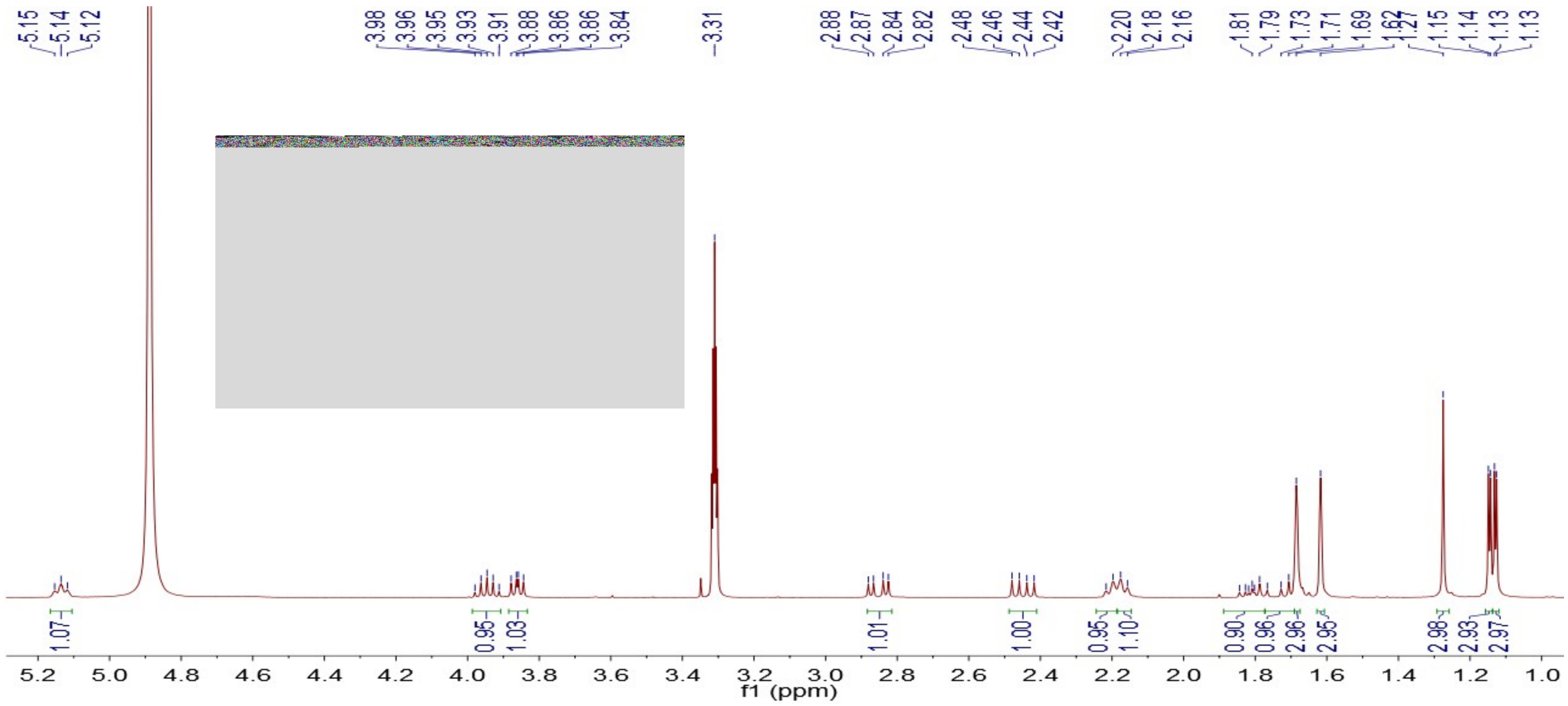

$^{13}C$ NMR of compound **2** (in methanol-$d_4$)

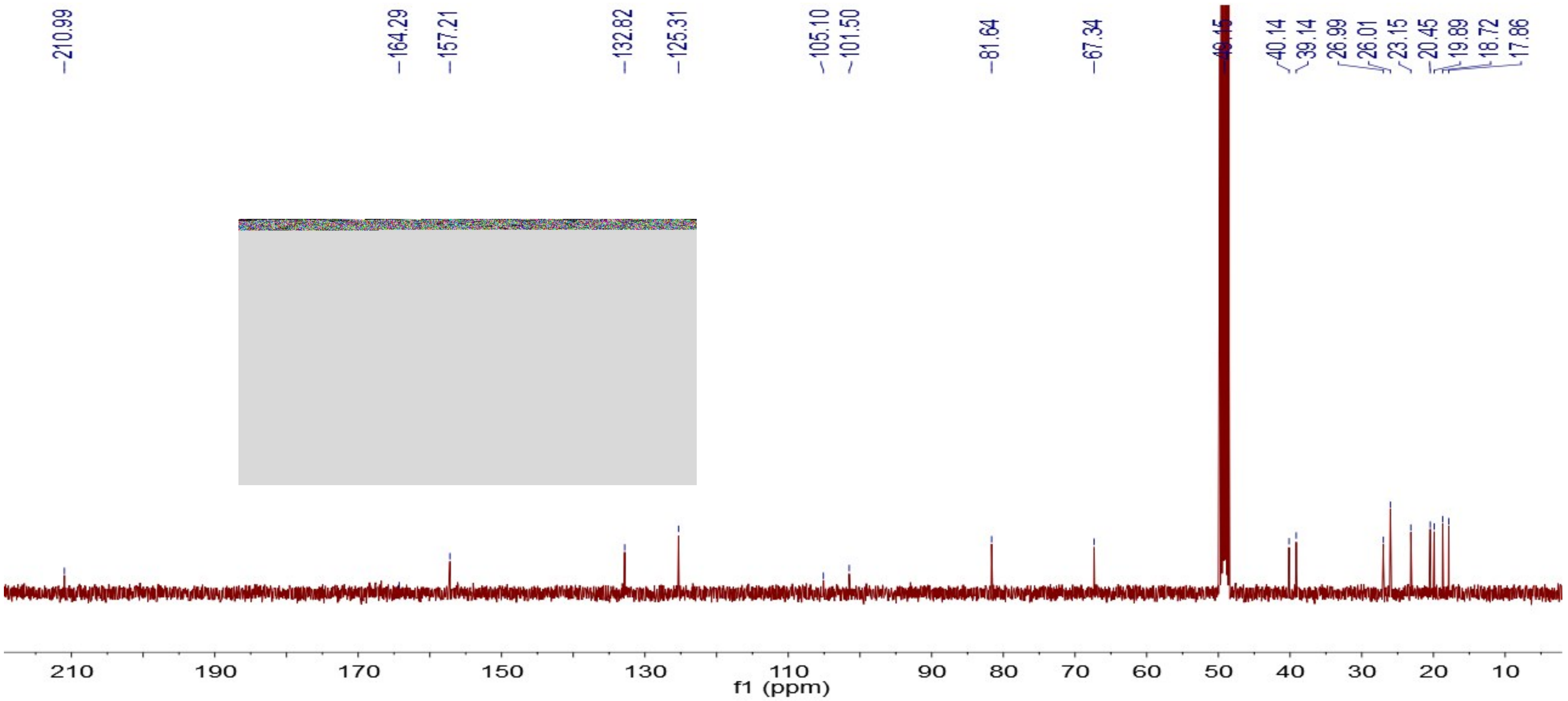


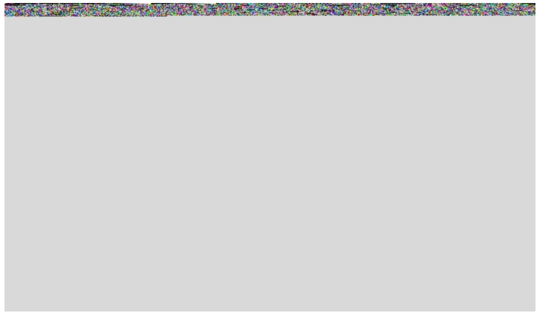

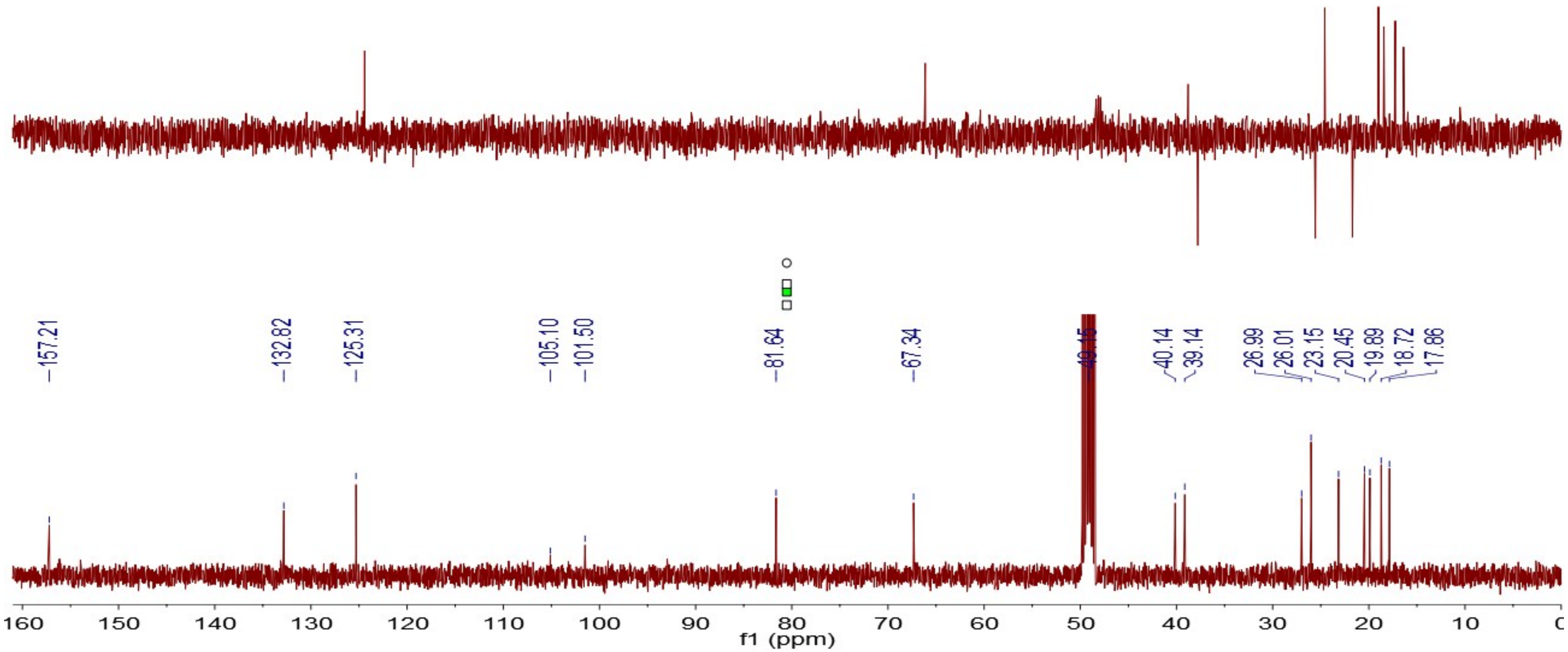

157.21
132.82
125.31
105.10
101.50
81.64
67.34
49.15
40.14
39.14
26.99
26.01
23.15
20.45
19.89
18.72
17.86
160
150
140
130
120
110
100
90
80
70
60
50
40
30
20
10
f1 (ppm)

HSQC of compound **2** (in methanol-$d_4$)

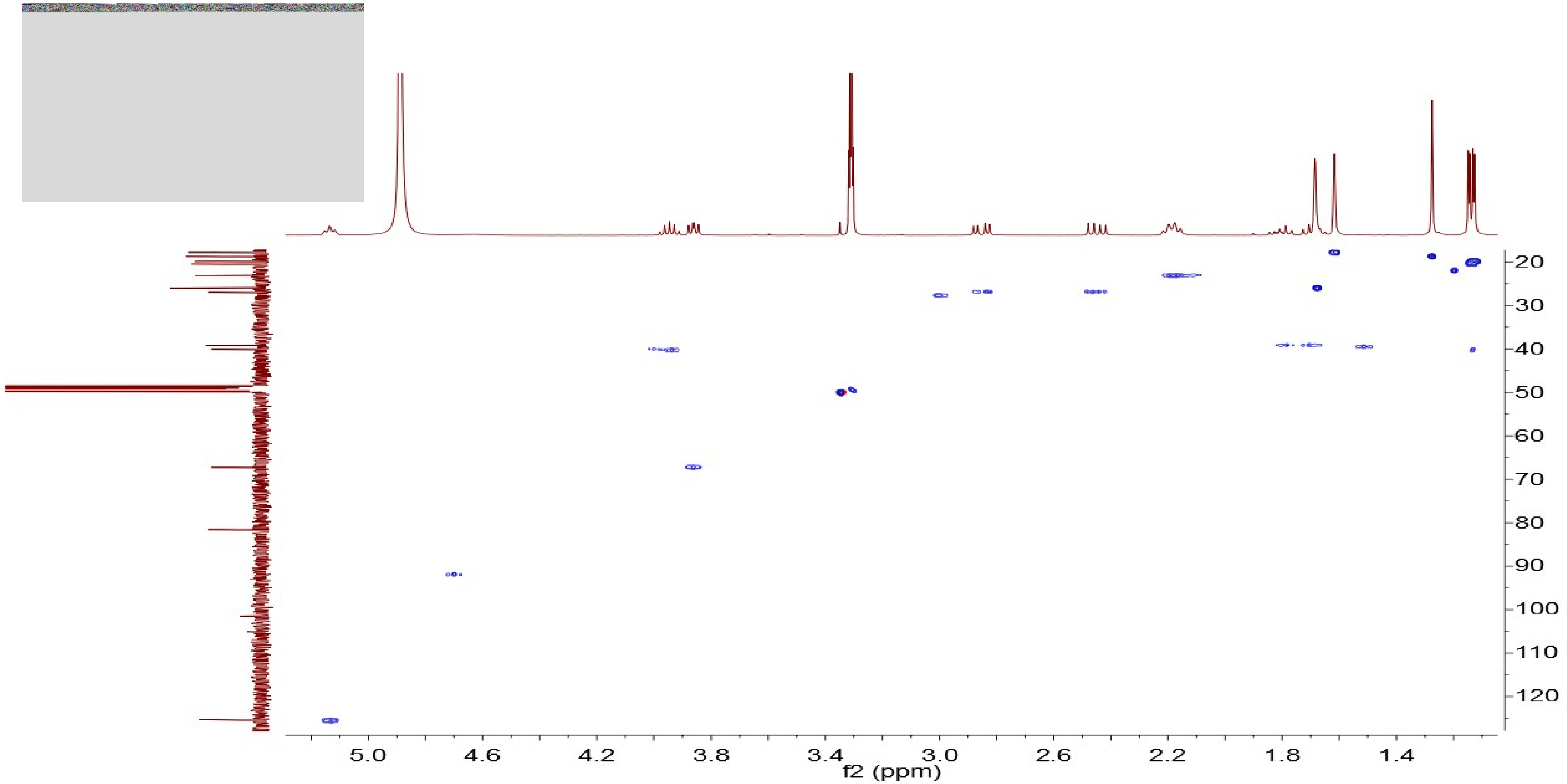

HMBC of compound **2** (in methanol-$d_4$)

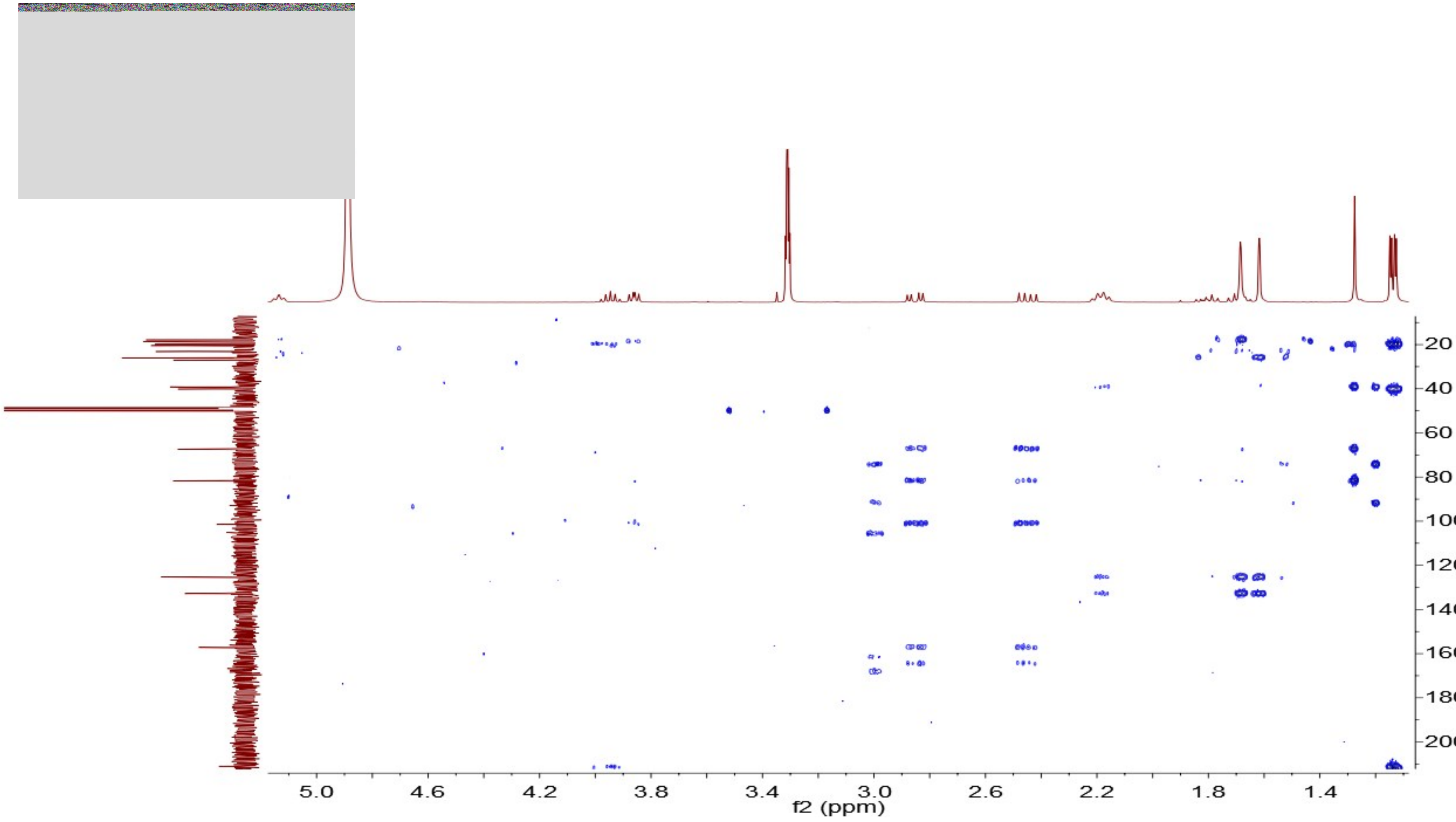

$^{1}$H–$^{1}$H COSY of compound **2** (in methanol-$d_4$)

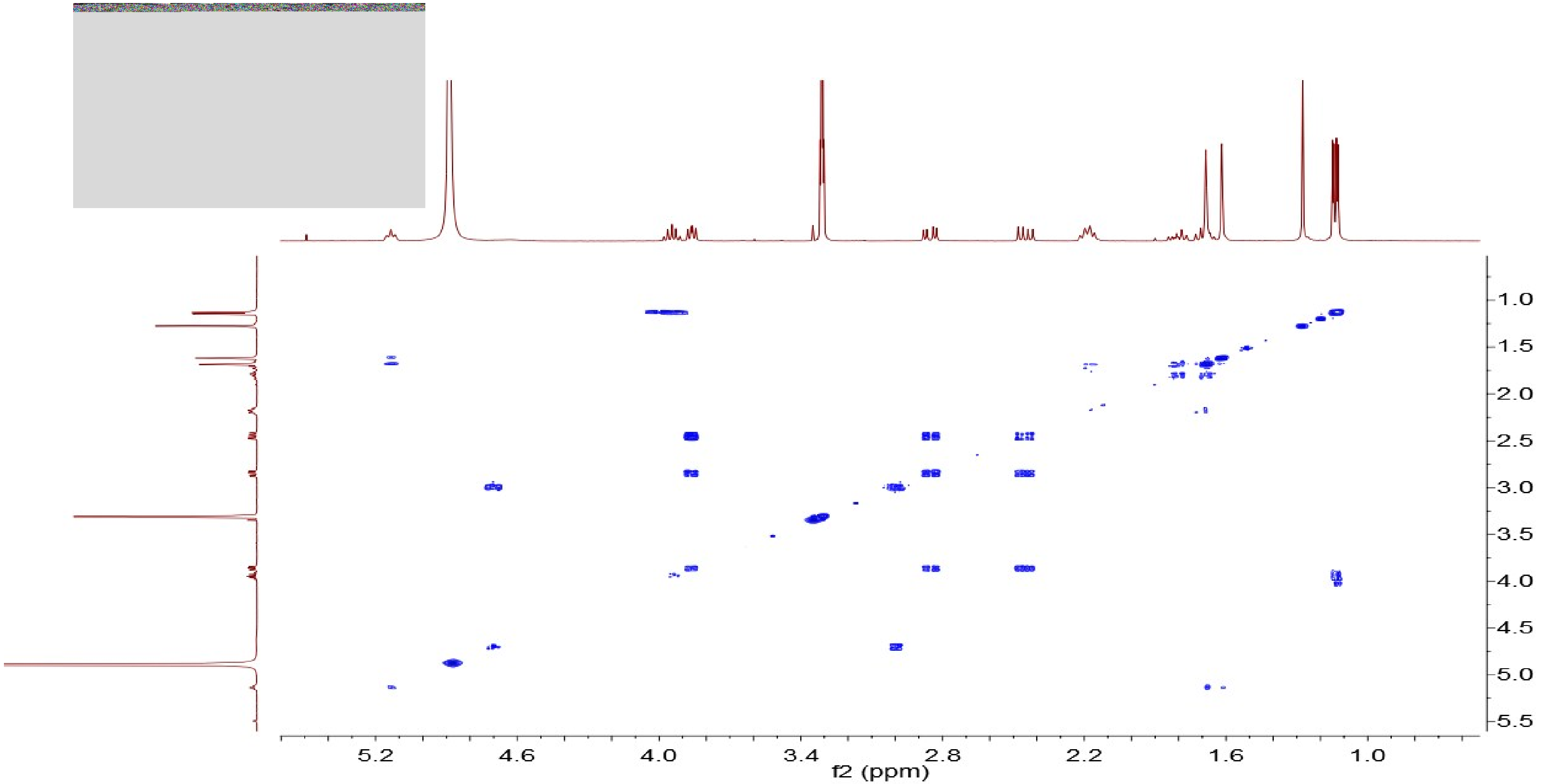

NOESY of compound **2** (in methanol-$d_4$)

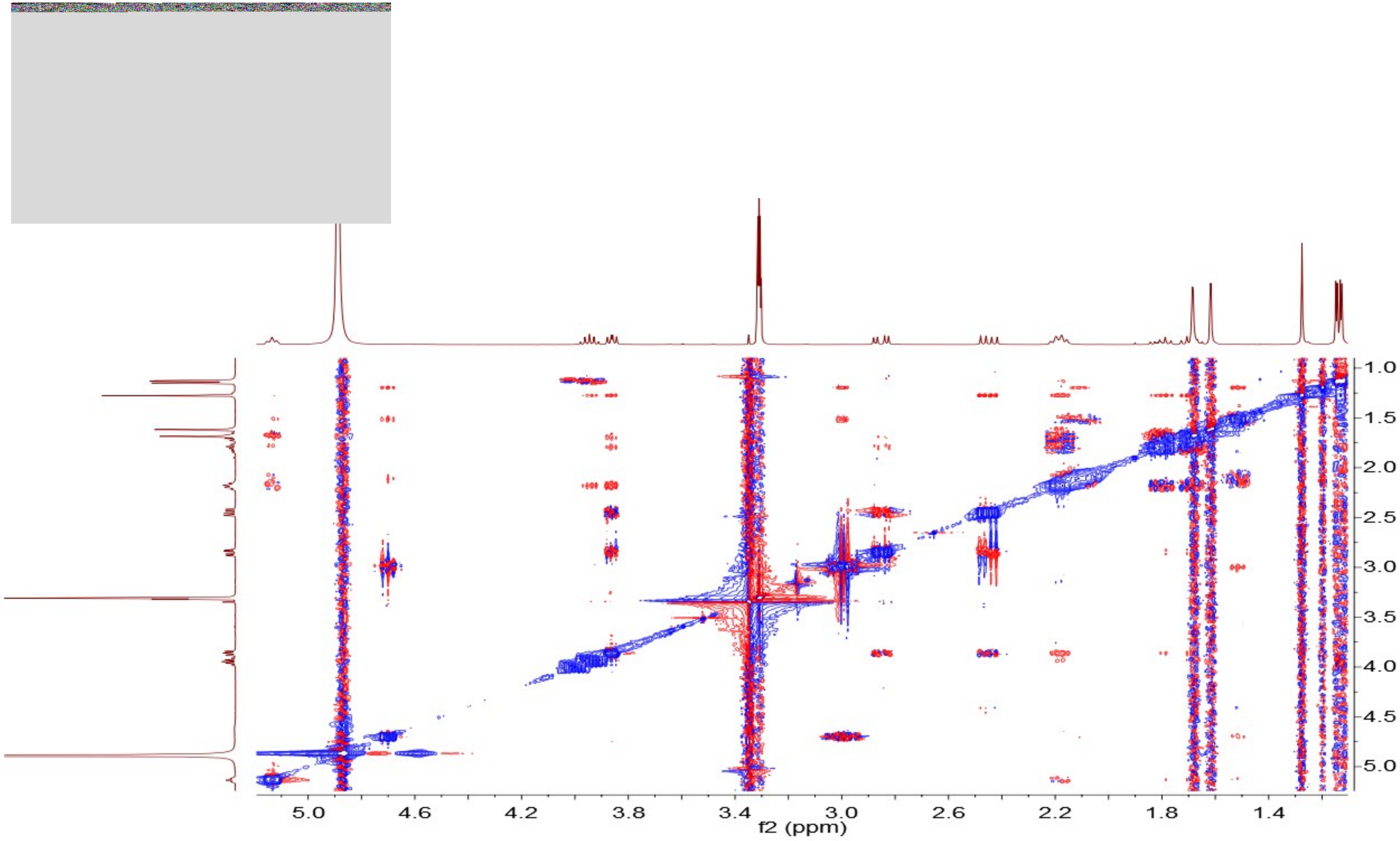

UV of compound **2**(in MeOH)

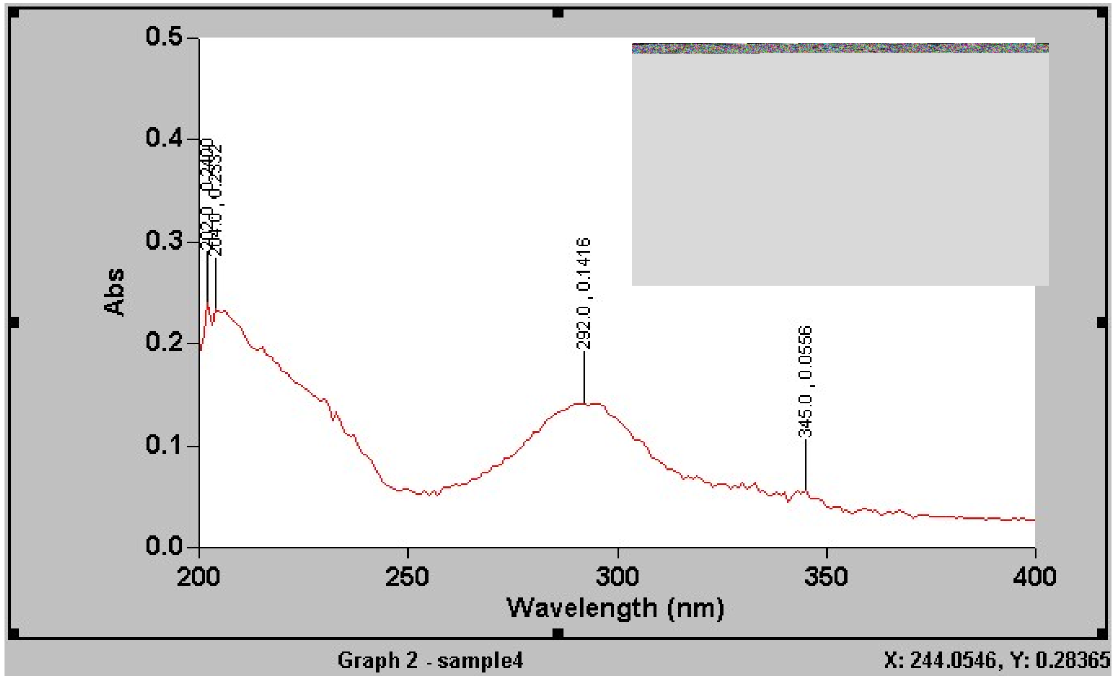

IR of compound **2**(KBr disc)

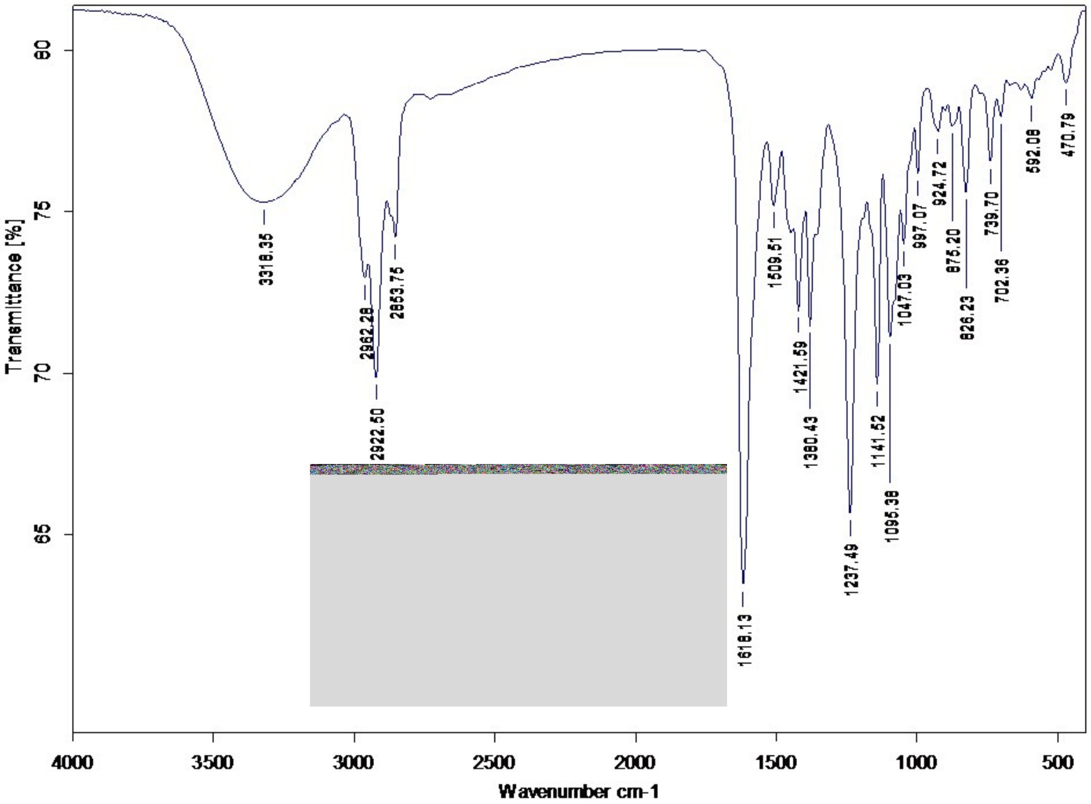

HRESIMS of compound **3**

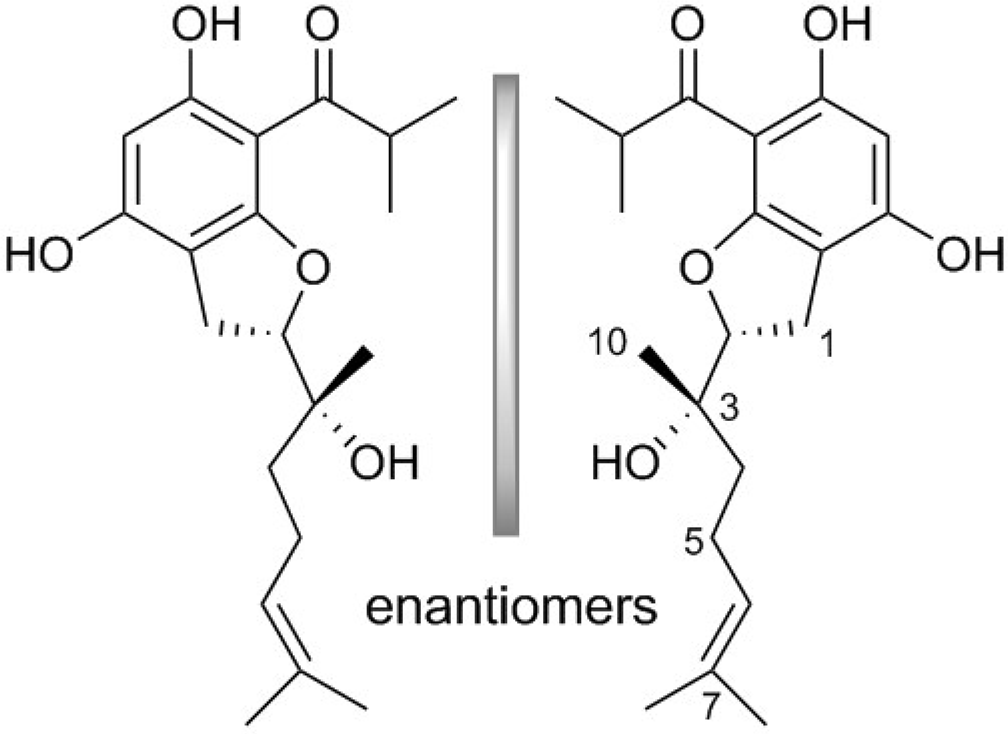


3a

3b

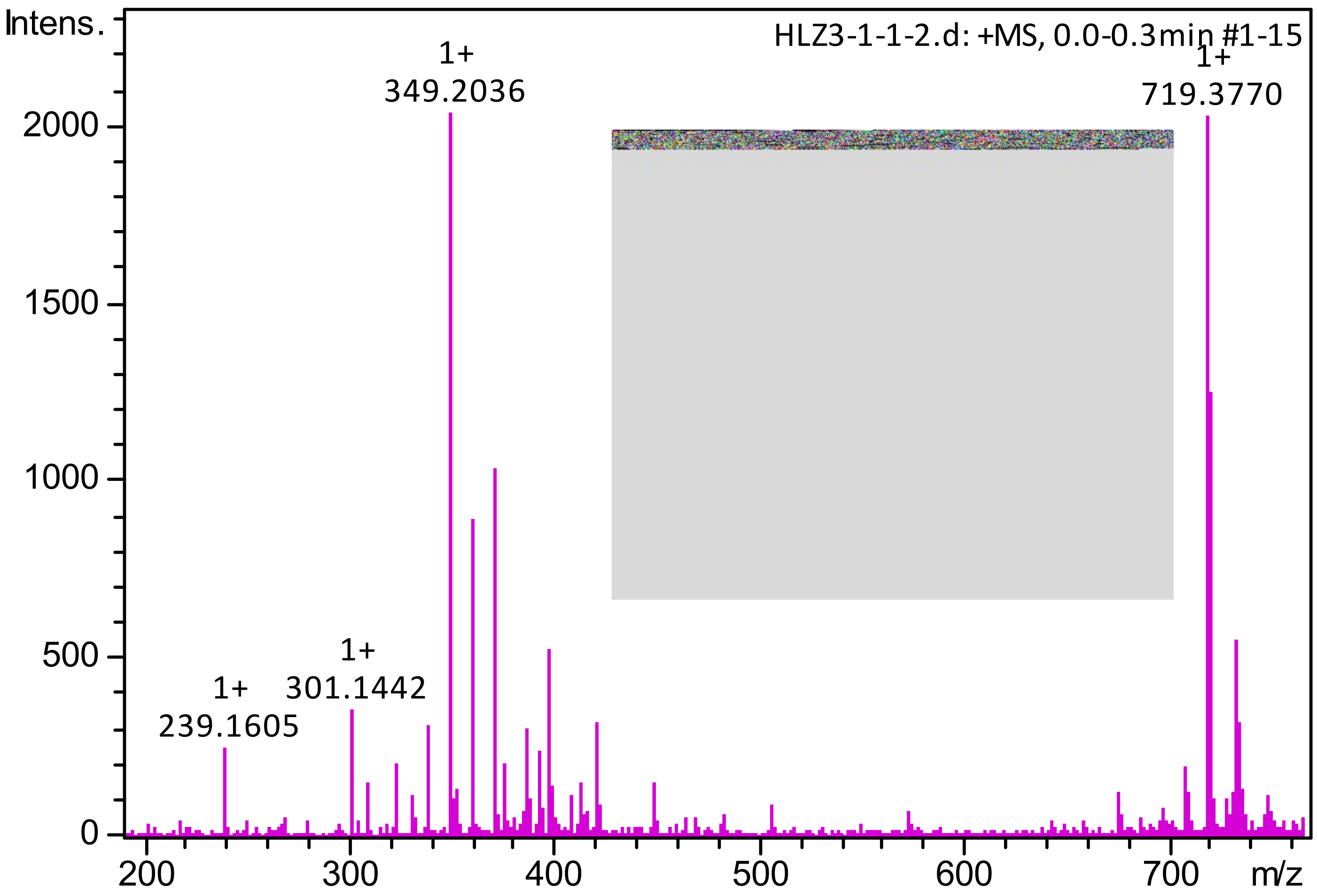


$^{1}$H NMR of compound **3** (in methanol-$d_4$)

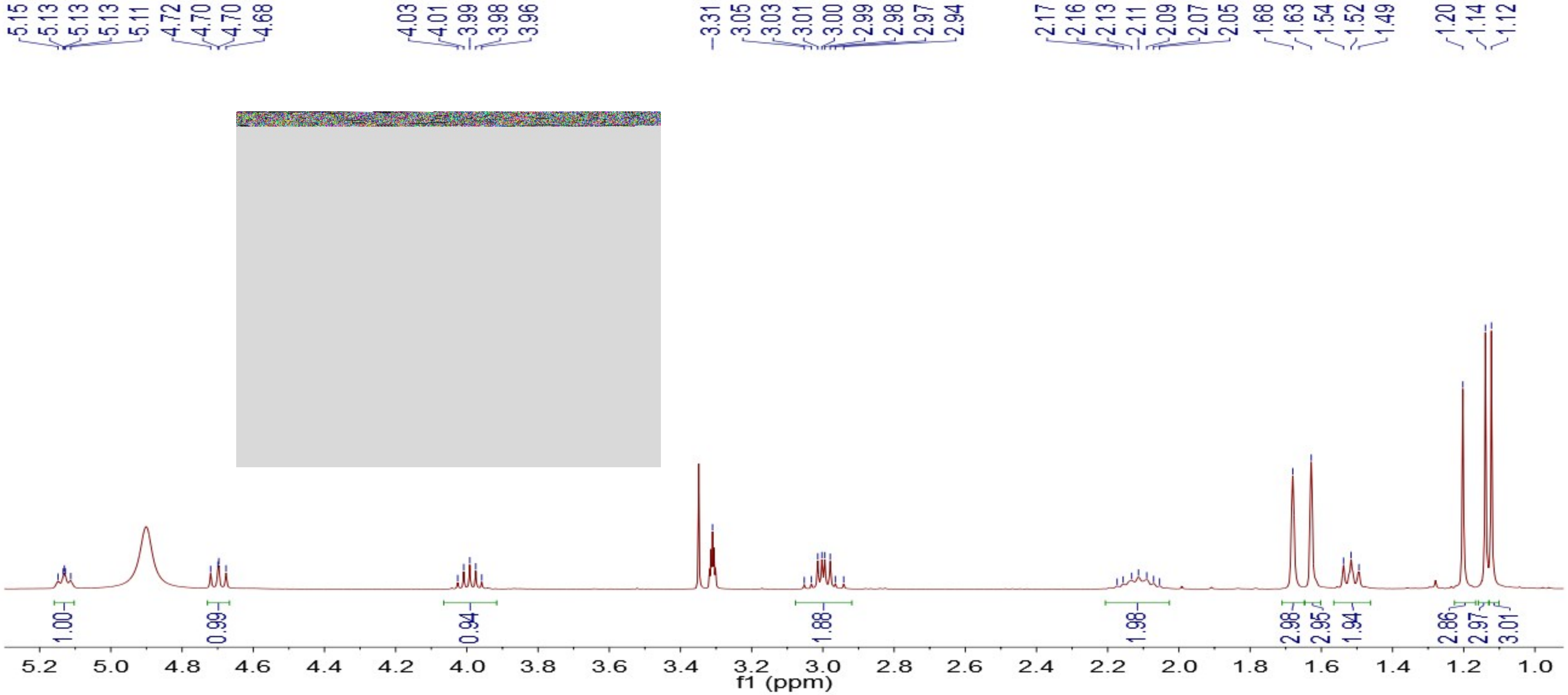
5.15 5.13 5.13 5.13 5.11 4.72 4.70 4.70 4.68
4.03 4.01 3.99 3.98 3.96
3.31 3.05 3.03 3.01 3.00 2.99 2.98 2.97 2.94
2.17 2.16 2.13 2.11 2.09 2.07 2.05 1.68 1.63 1.54 1.52 1.49
1.20 1.14 1.12
1.00 0.99 0.94 1.88 1.98 2.98 2.95 1.94 2.86 2.97 3.01
5.2 5.0 4.8 4.6 4.4 4.2 4.0 3.8 3.6 3.4 3.2 3.0 2.8 2.6 2.4 2.2 2.0 1.8 1.6 1.4 1.2 1.0
f1 (ppm)

$^{13}C$ NMR of compound **3** (in methanol-$d_4$)

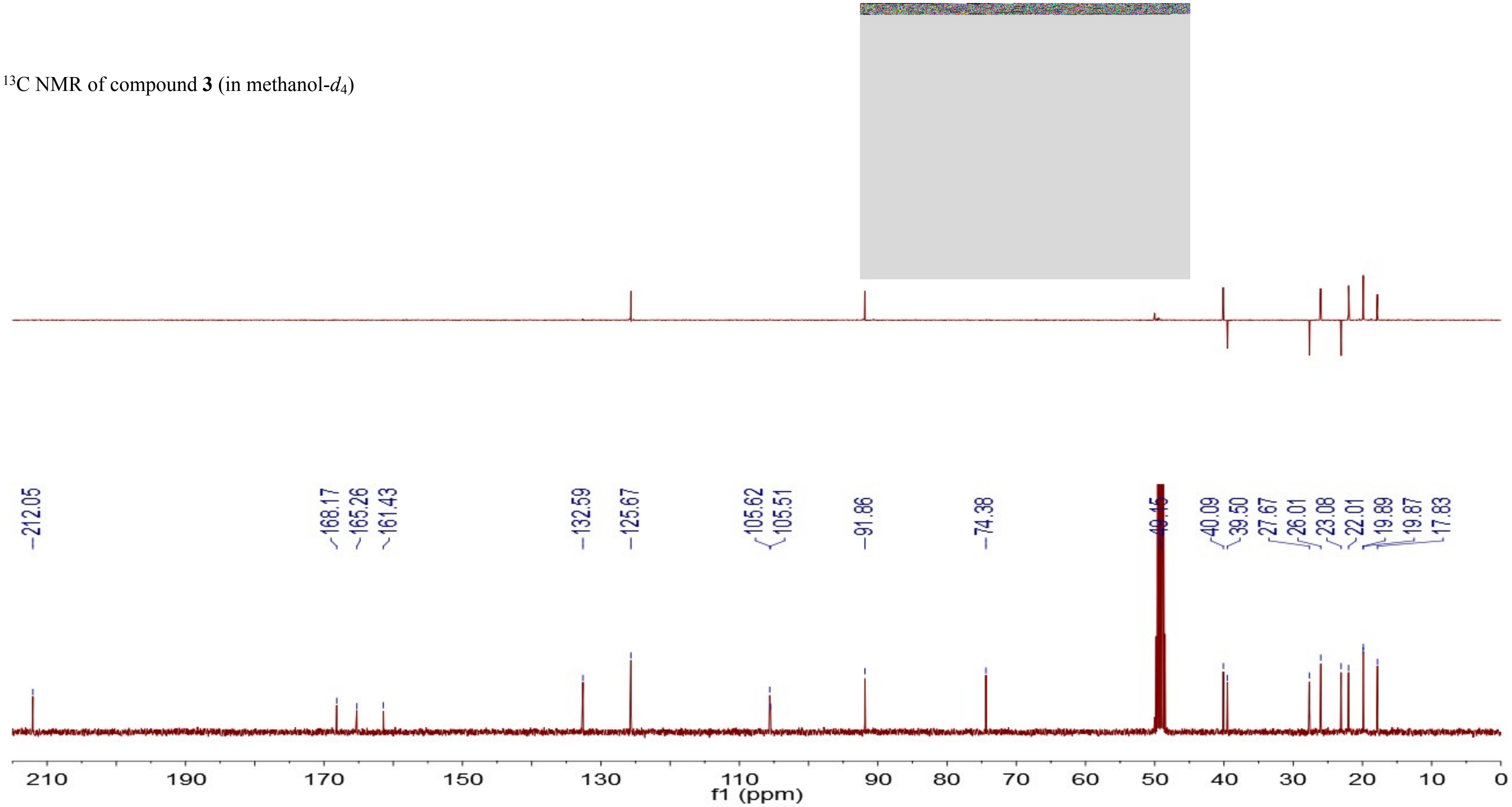

HSQC of compound **3** (in methanol-$d_4$)

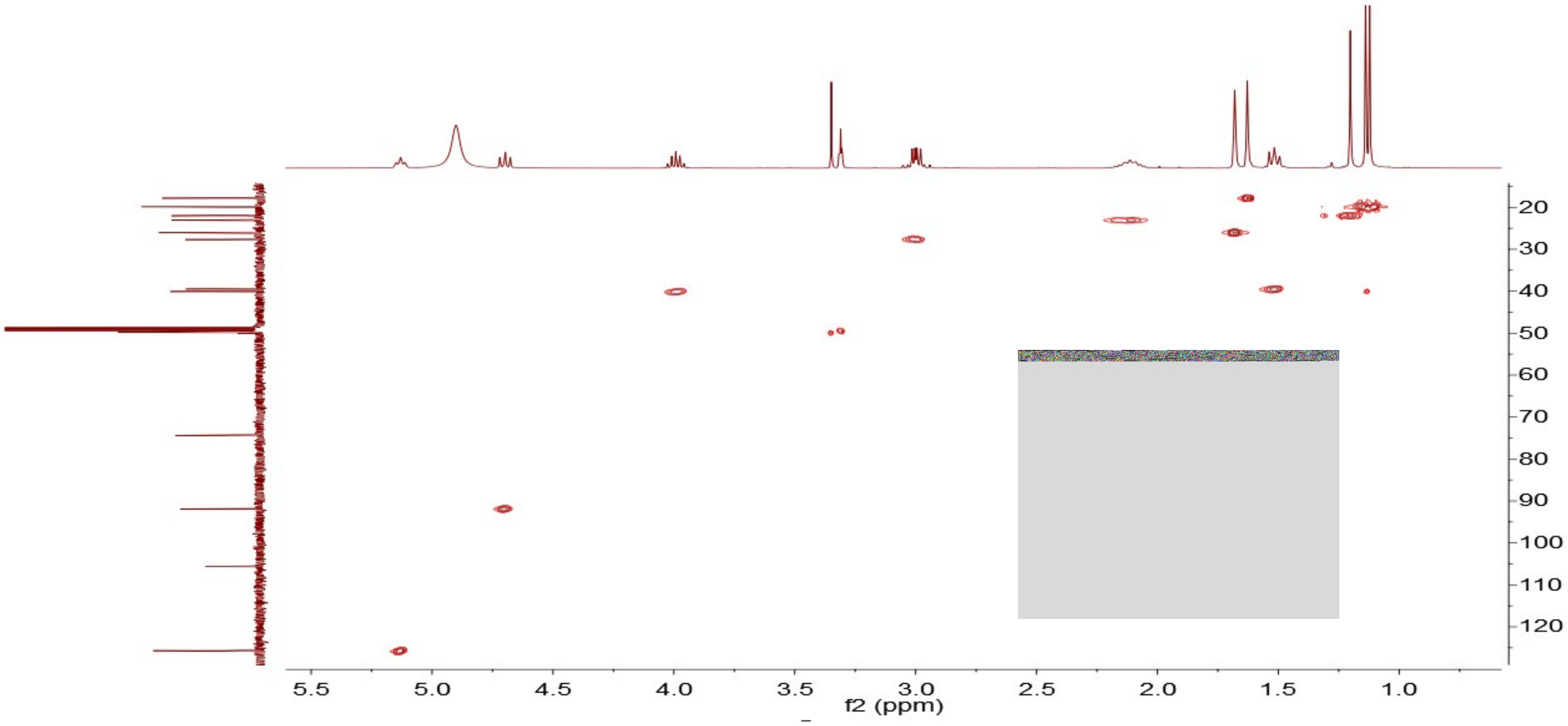

HMBC of compound **3** (in methanol-$d_4$)

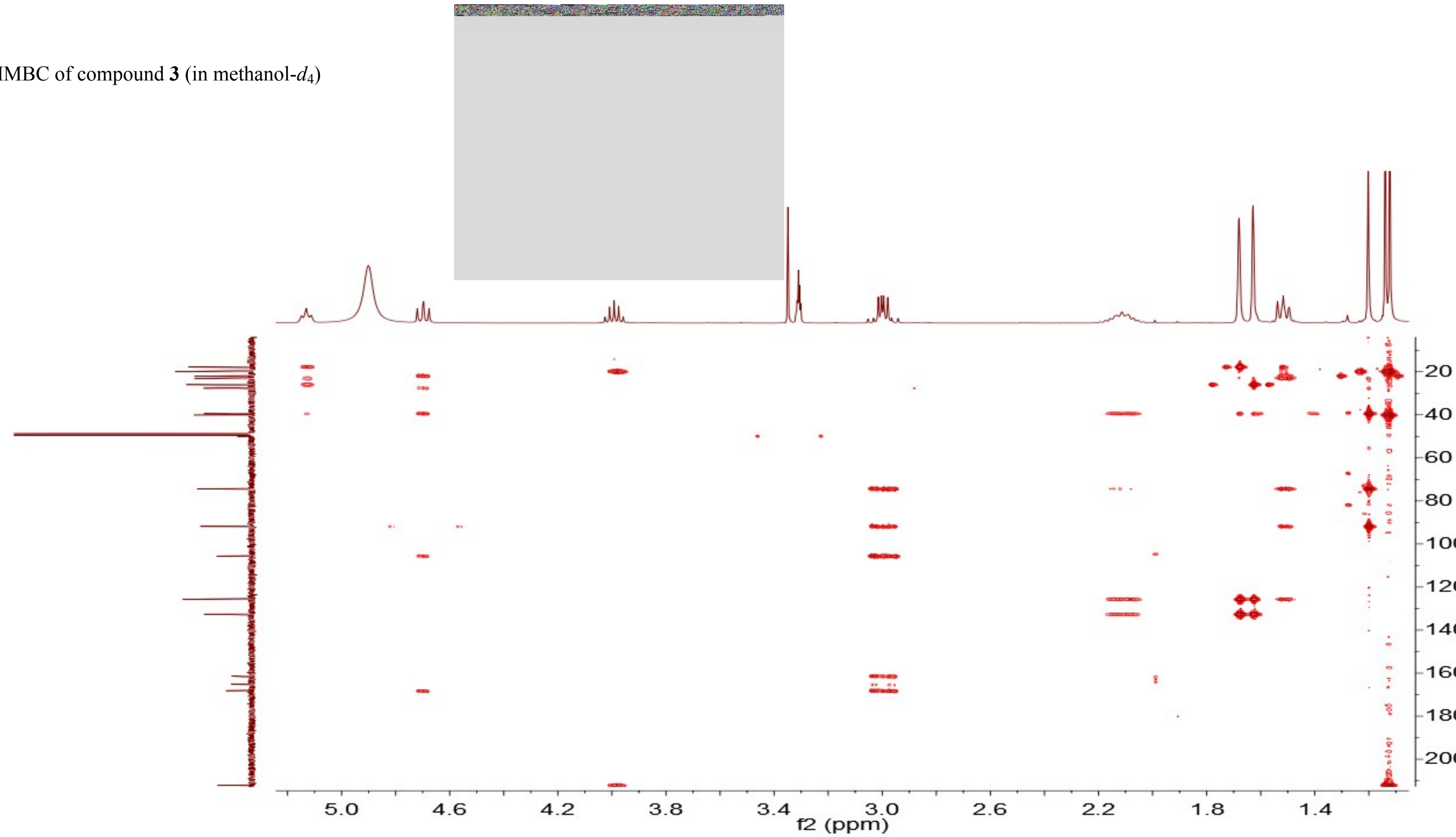

$^1H$–$^1H$ COSY of compound **3** (in methanol-$d_4$)

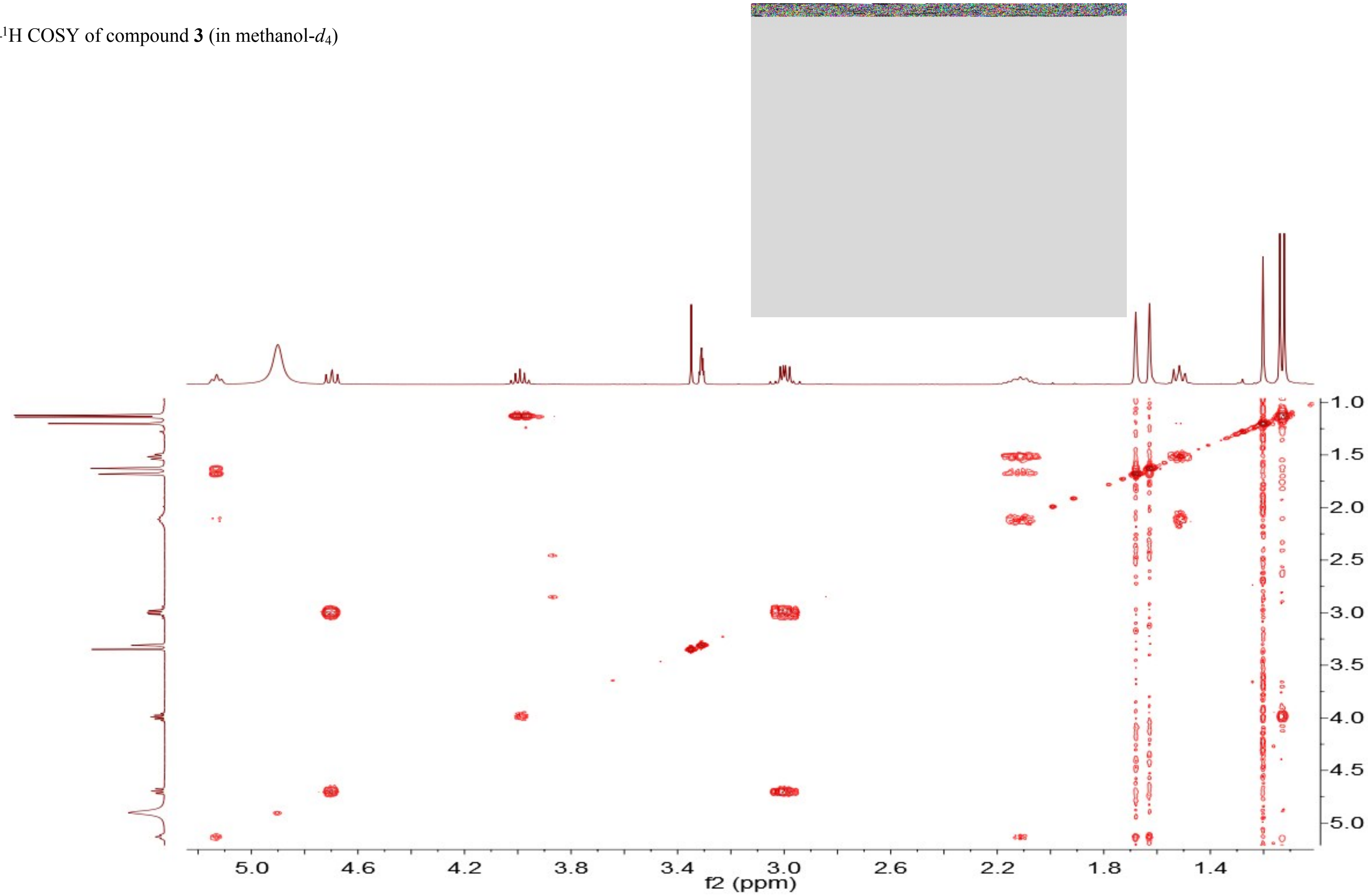

NOESY of compound **3** (in methanol-$d_4$)

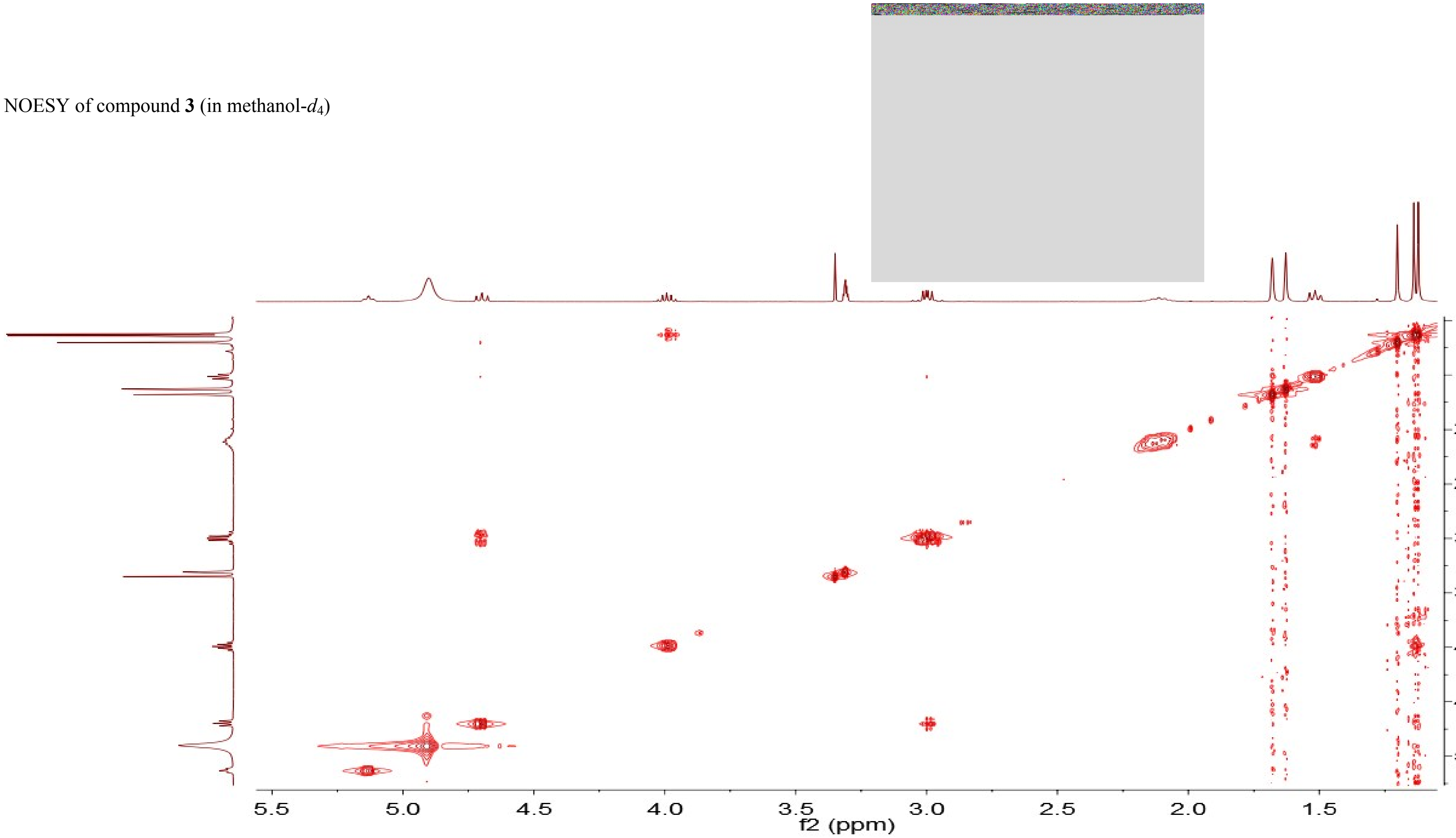

UV of compound **3**(in MeOH)

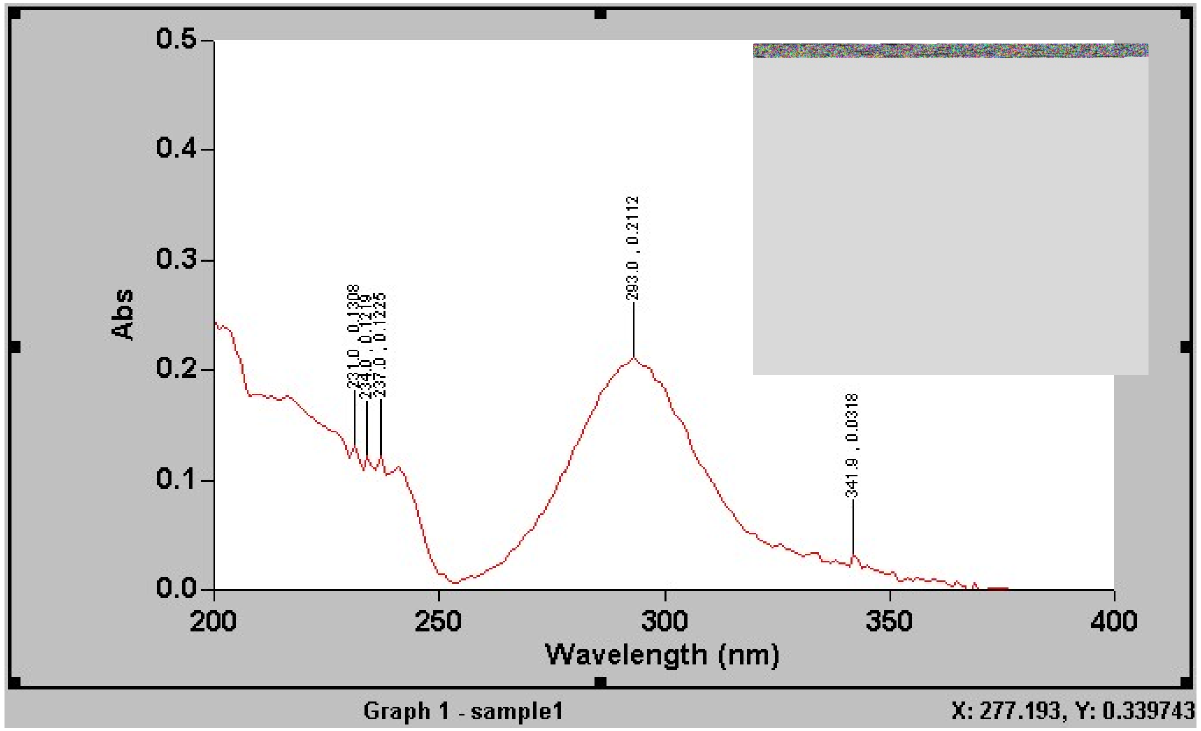

IR of compound **3** (KBr disc)

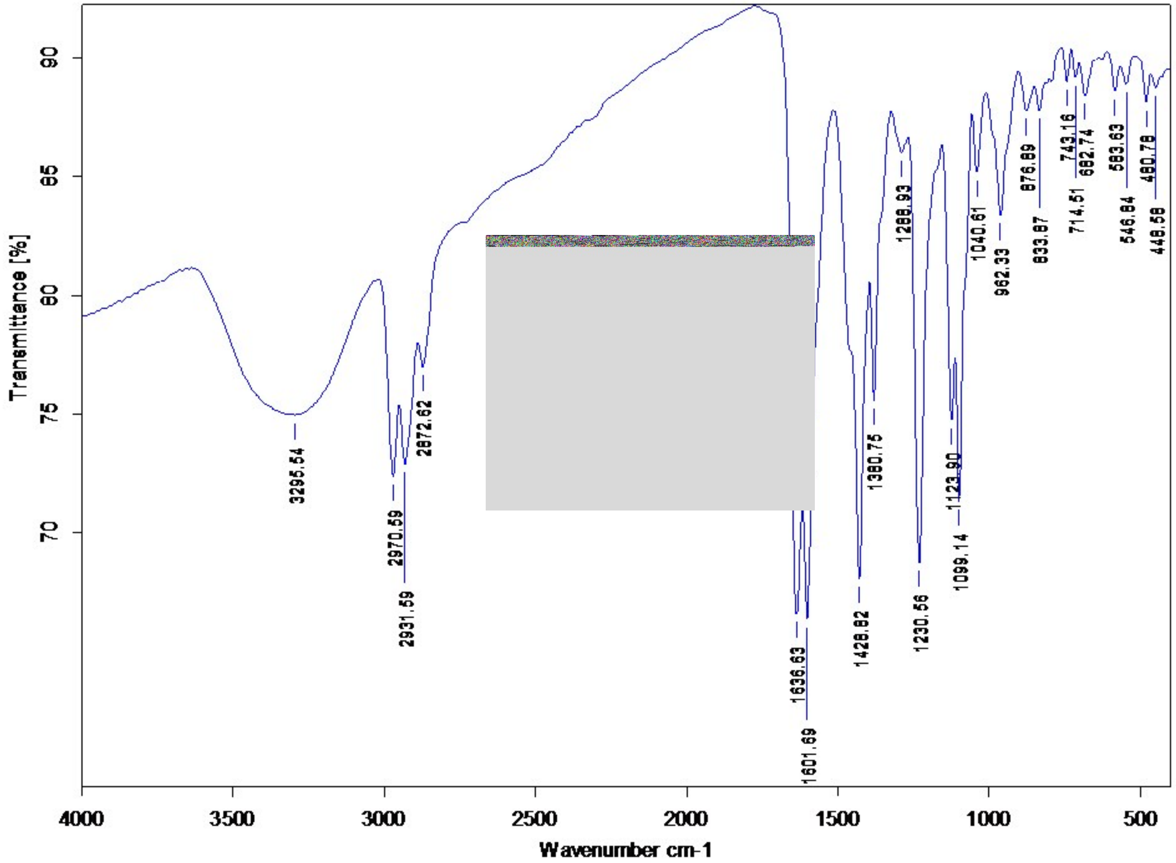

HRESIMS of compound **4**

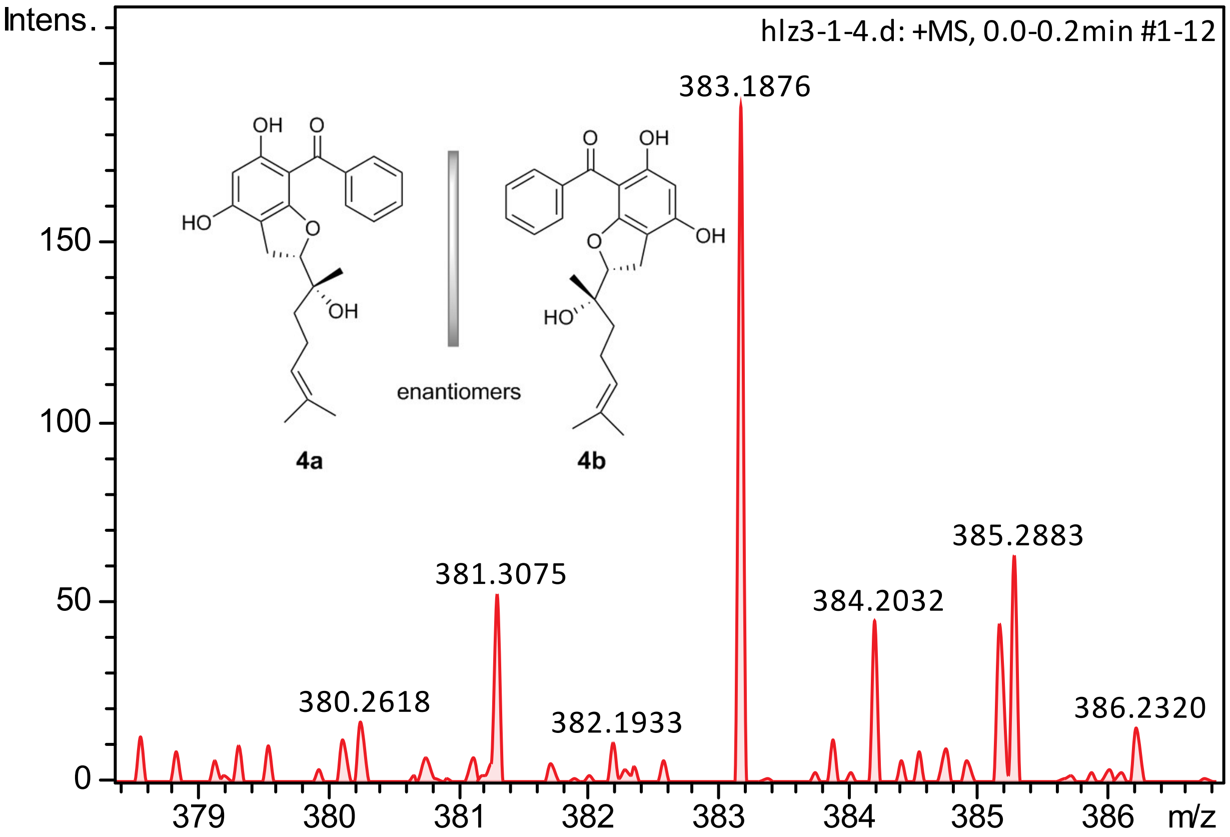

$^{1}$H NMR of compound **4** (in methanol-$d_4$)

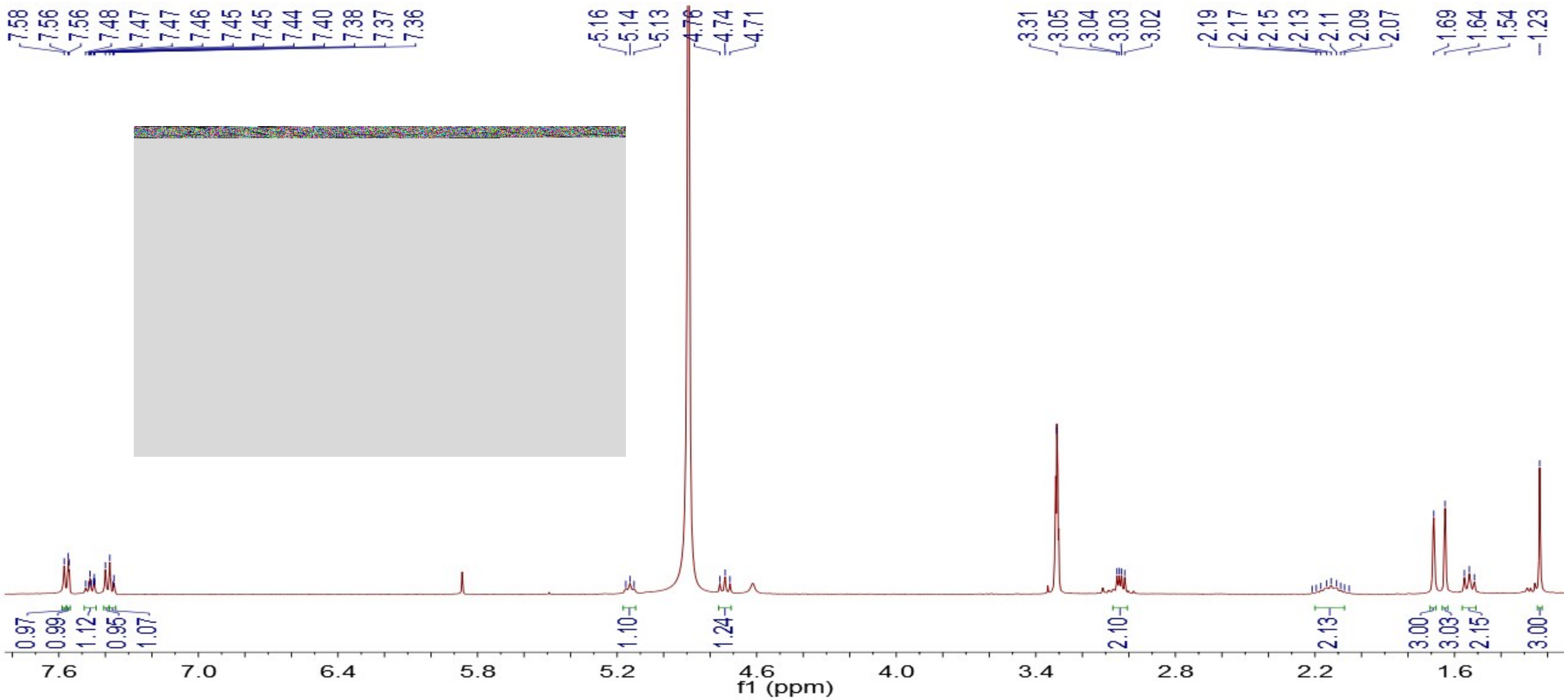

$^{13}$C NMR of compound **4** (in methanol-$d_4$)

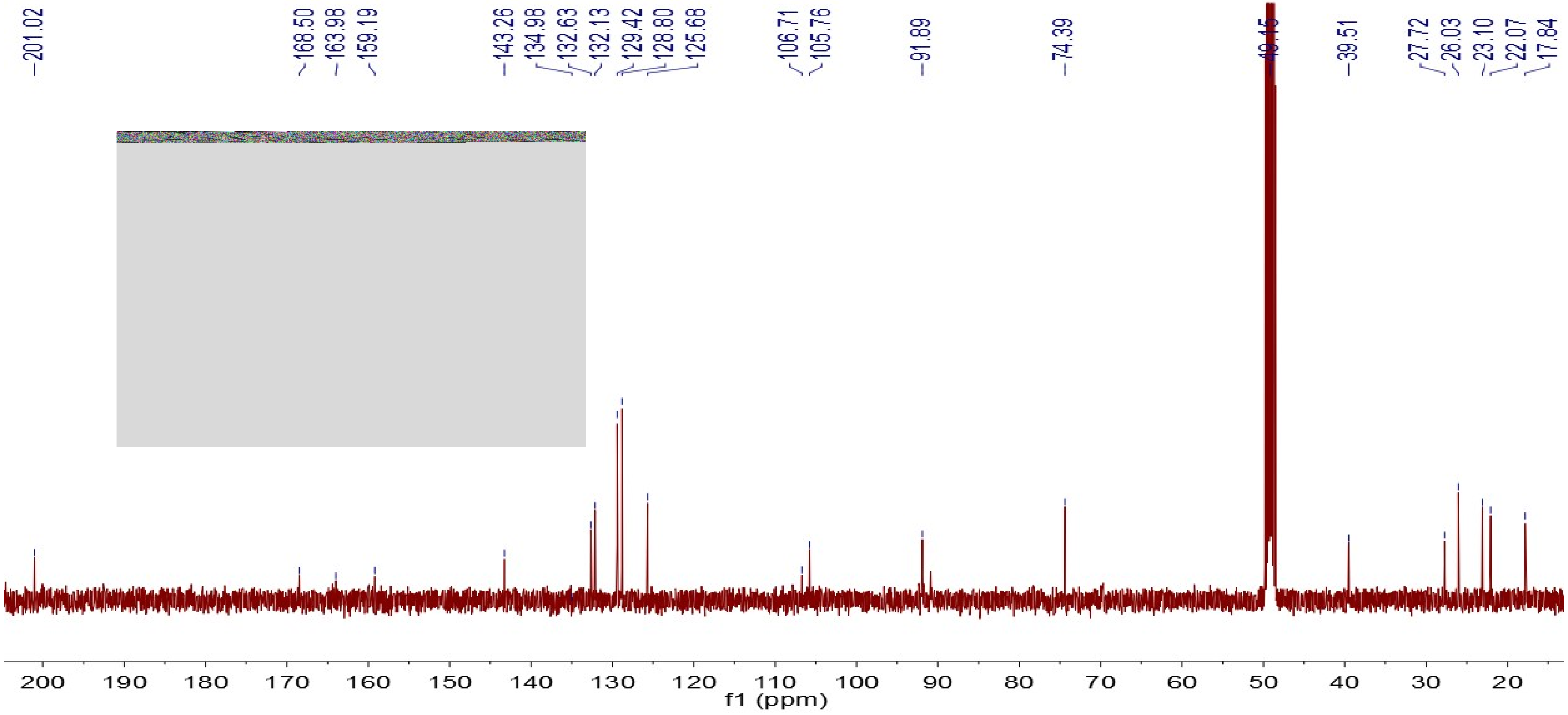

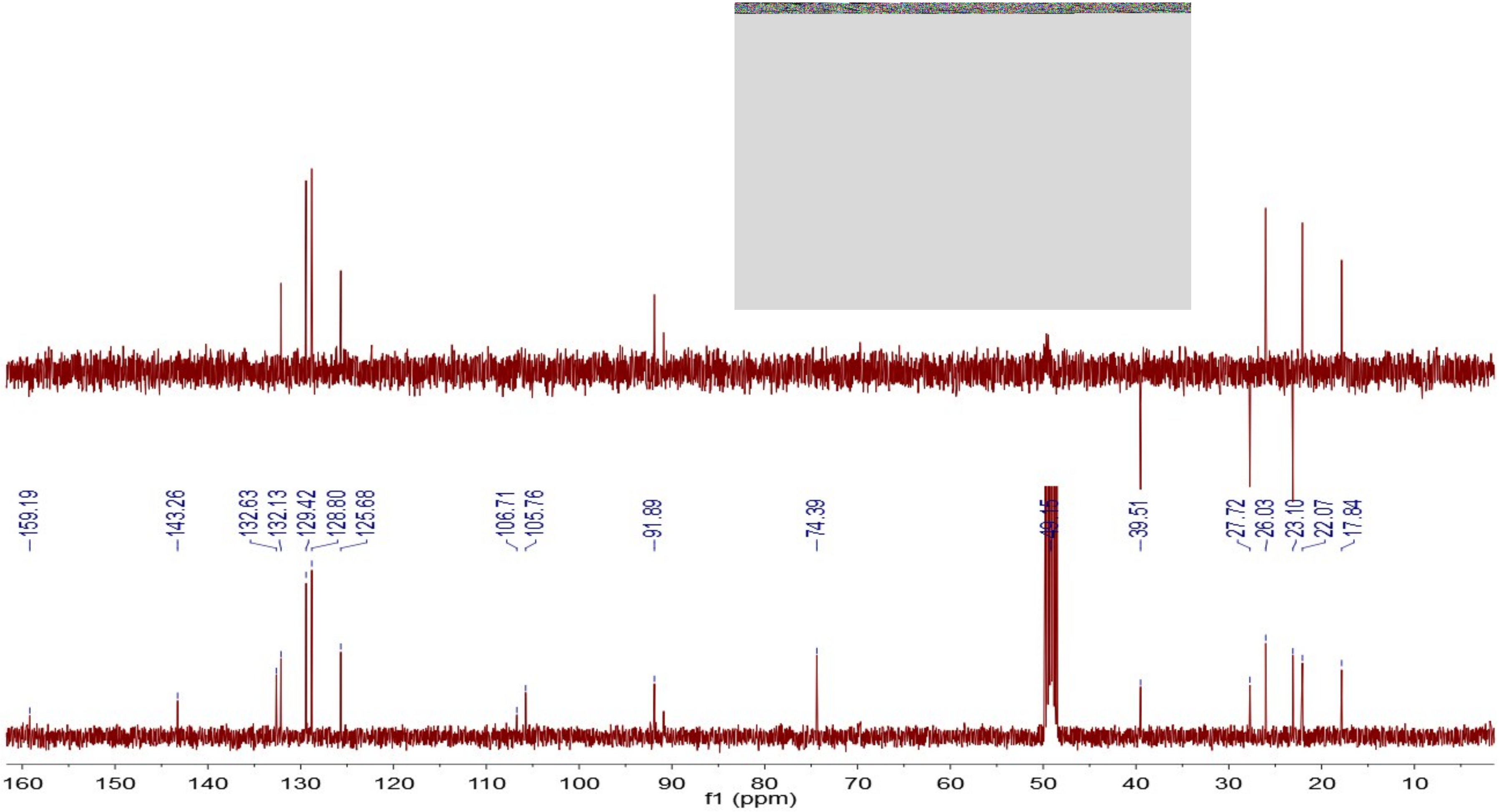
159.19
143.26
132.63
132.13
129.42
128.80
125.68
106.71
105.76
91.89
74.39
49.15
39.51
27.72
26.03
23.10
22.07
17.84
160 150 140 130 120 110 100 90 80 70 60 50 40 30 20 10
f1 (ppm)

HSQC of compound **4** (in methanol-$d_4$)

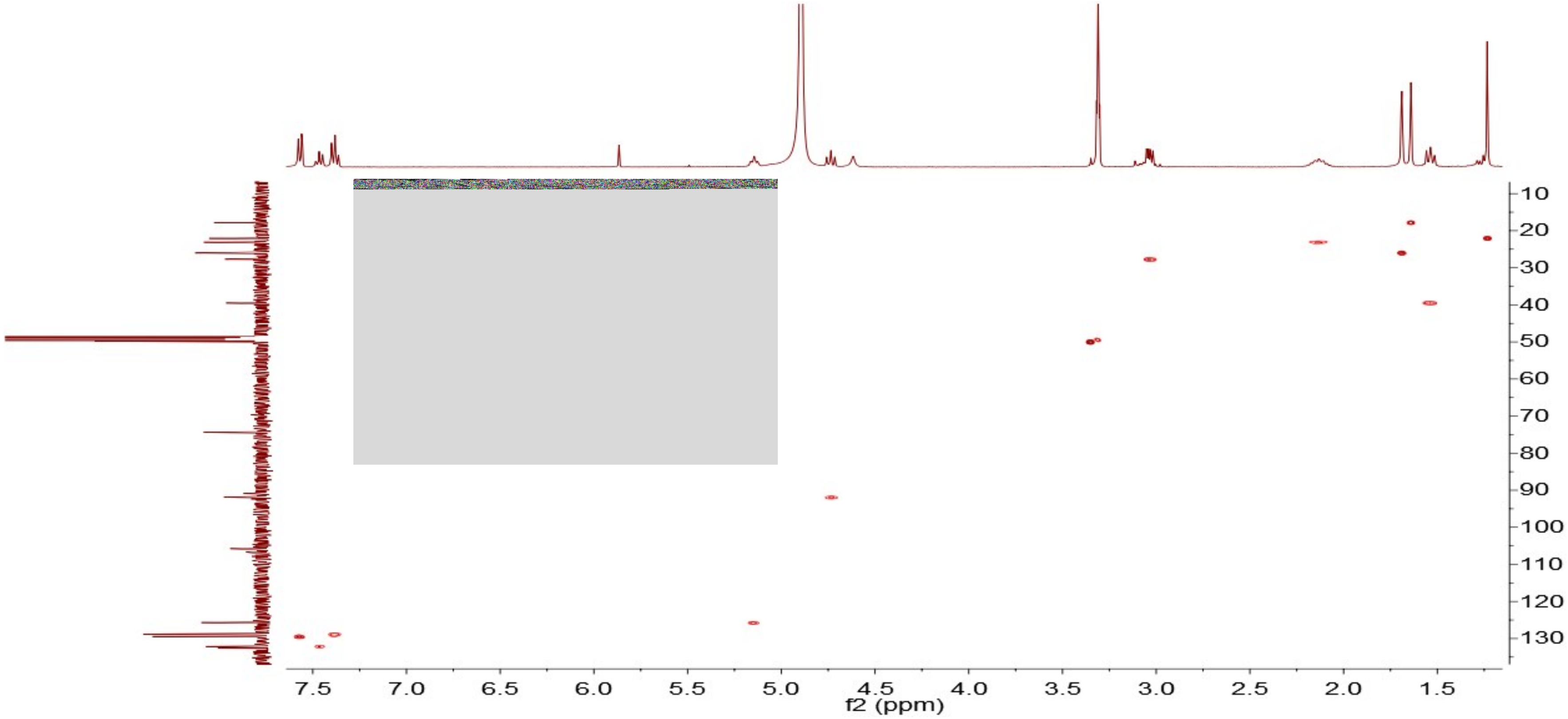

HMBC of compound **4** (in methanol-$d_4$)

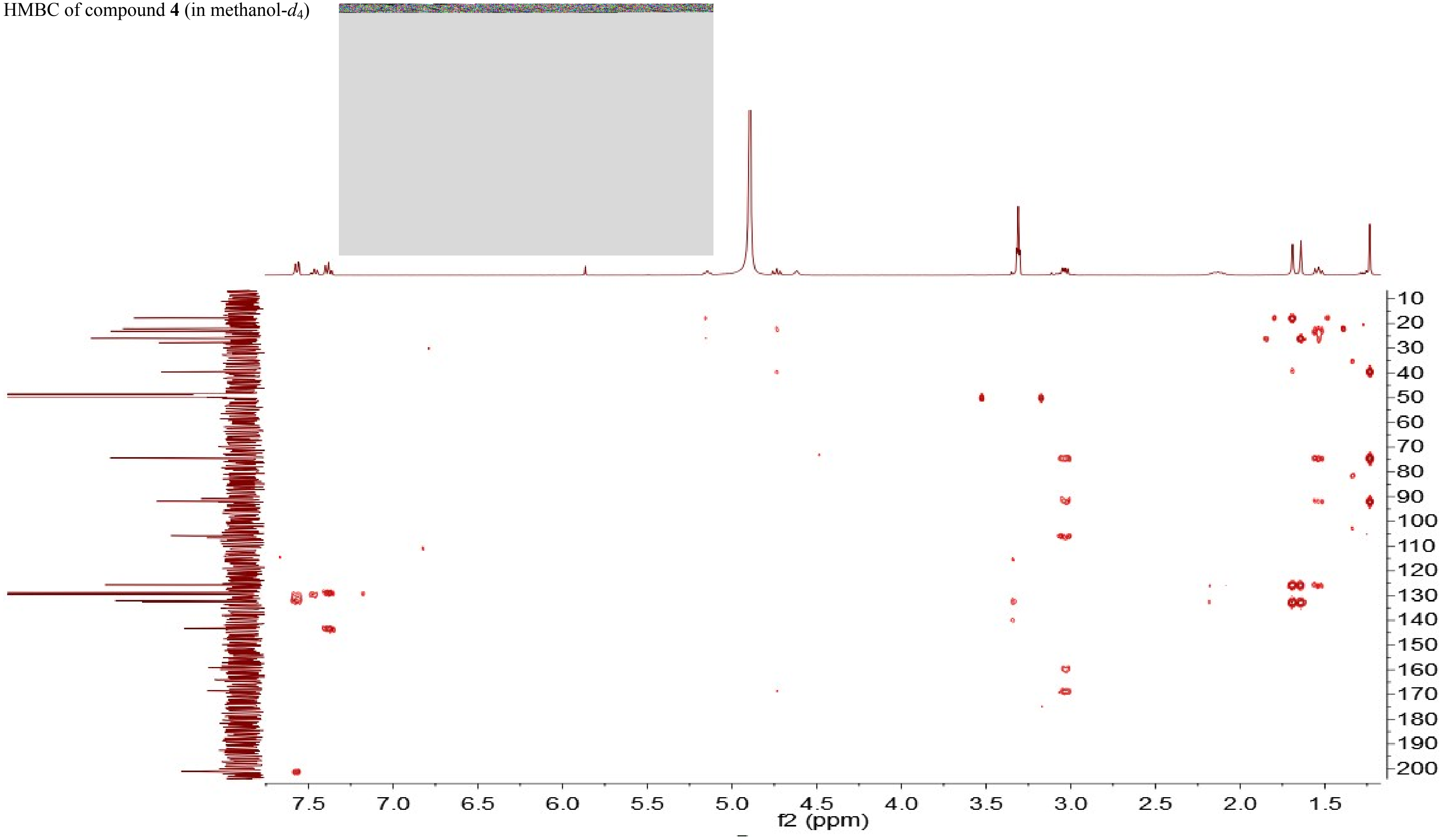

$^{1}$H–$^{1}$H COSY of compound **4** (in methanol-$d_4$)

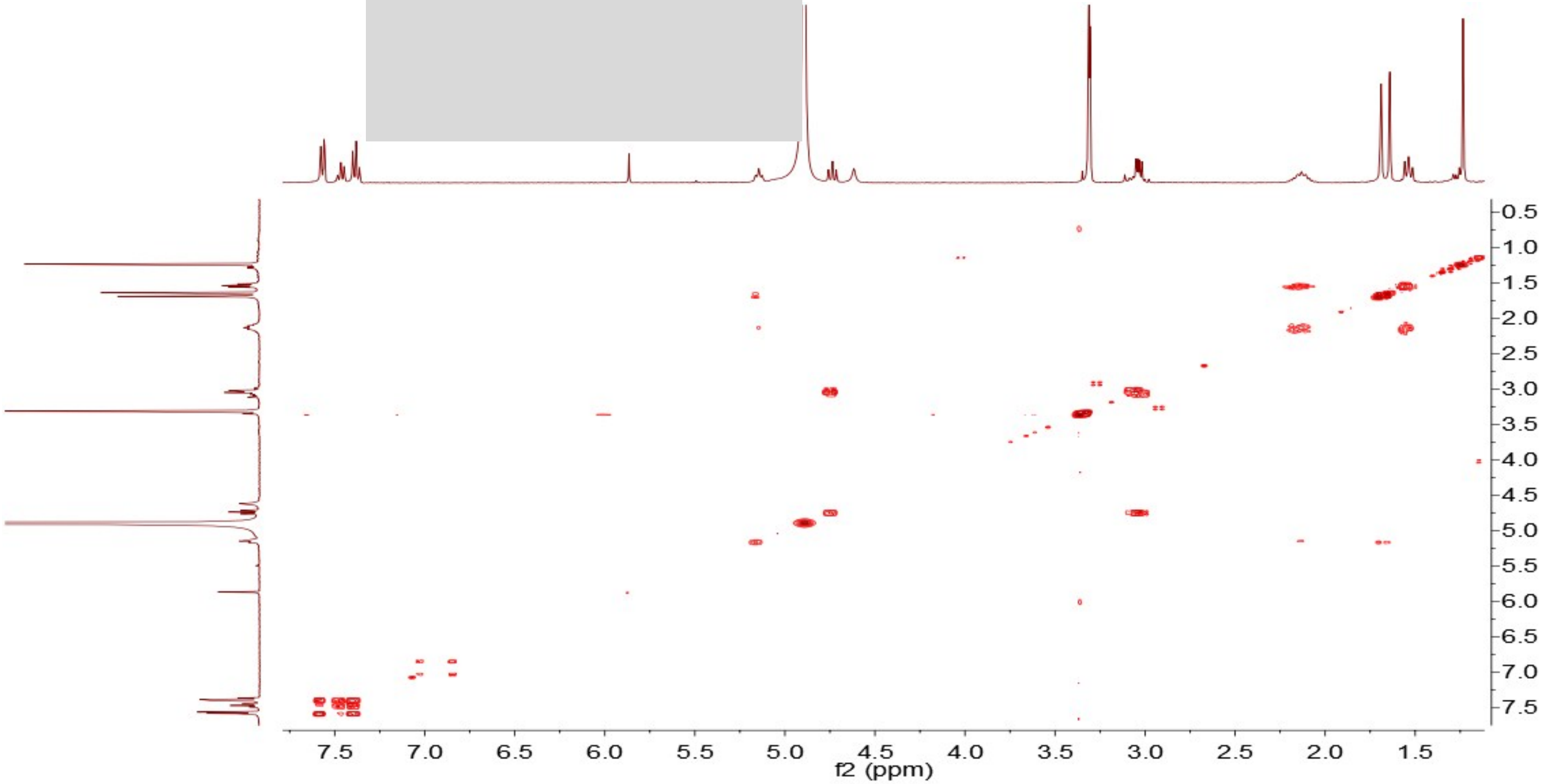

NOESY of compound **4** (in methanol-$d_4$)

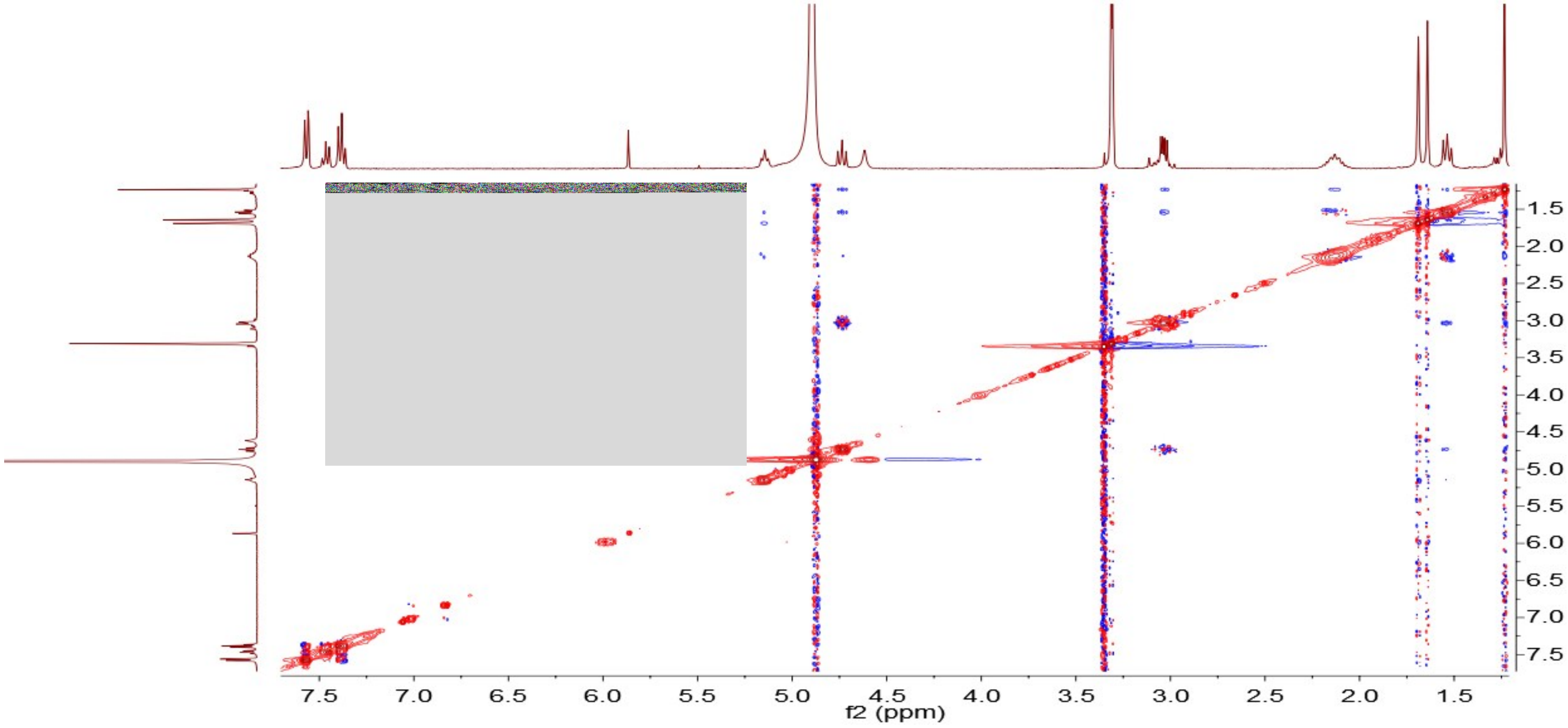

UV of compound **4** (in MeOH)

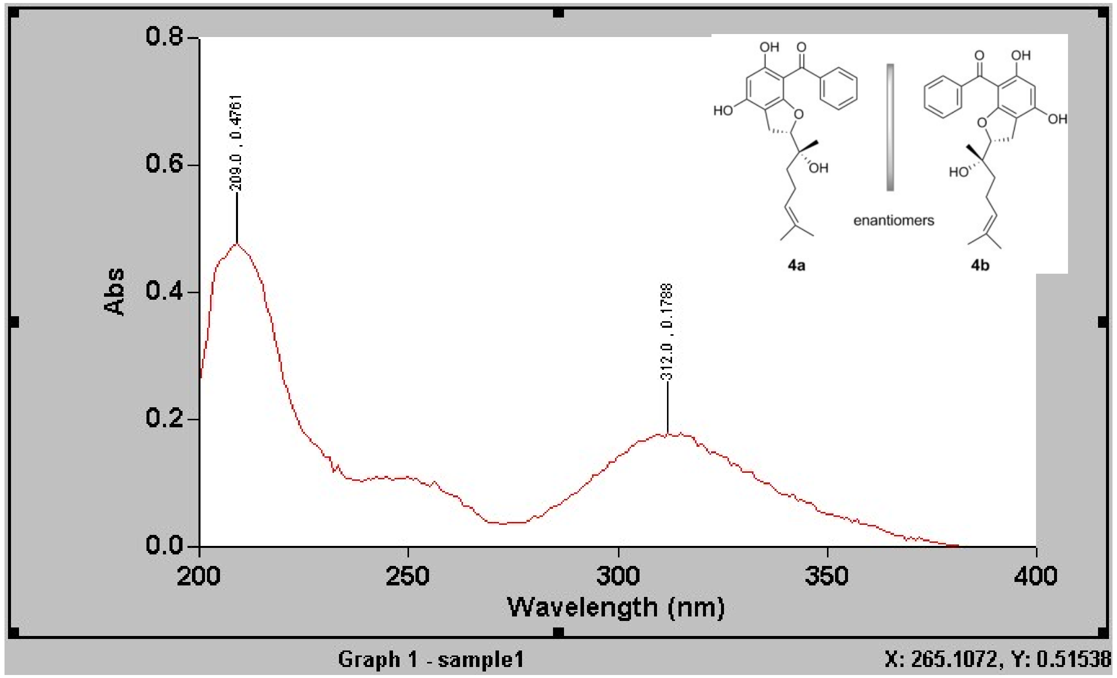

IR of compound **4** (KBr disc)

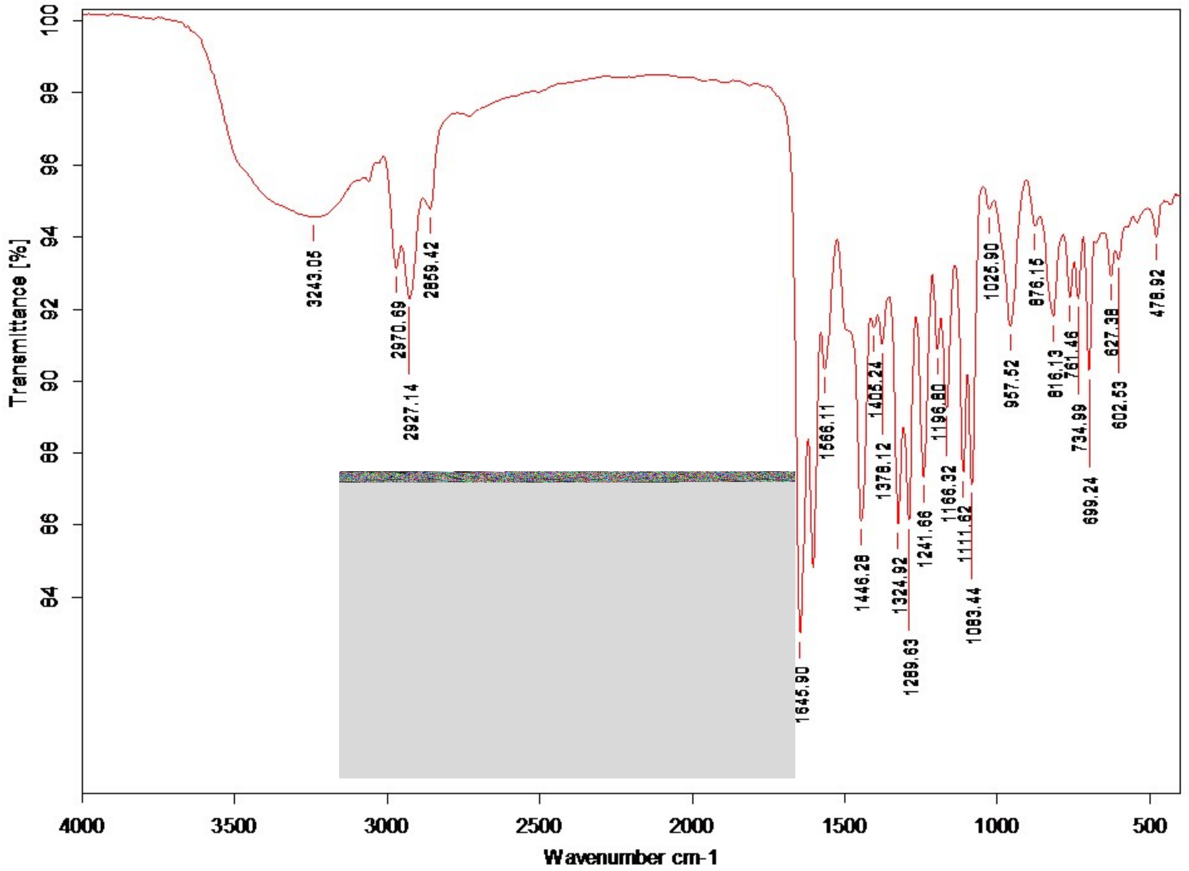

Supplement: RA-008-C8RA04073G-s001 [file RA-008-C8RA04073G-s001.pdf]
